# Chromatin profiling identifies chondrocyte-specific Sox9 enhancers important for skeletal development

**DOI:** 10.1172/jci.insight.175486

**Published:** 2024-06-10

**Authors:** Sachi Ichiyama-Kobayashi, Kenji Hata, Kanta Wakamori, Yoshifumi Takahata, Tomohiko Murakami, Hitomi Yamanaka, Hiroshi Takano, Ryoji Yao, Narikazu Uzawa, Riko Nishimura

**Affiliations:** 1 Department of Molecular and Cellular Biochemistry,; 2 Department of Oral and Maxillofacial Oncology and Surgery, and; 3 Genome Editing Research and Development Unit, Osaka University Graduate School of Dentistry, Suita, Osaka, Japan.; 4 Department of Cell Biology, Cancer Institute, Japanese Foundation for Cancer Research, Koto-ku, Tokyo, Japan.

**Keywords:** Bone biology, Cartilage, Epigenetics

## Abstract

The transcription factor SRY-related HMG box 9 (Sox9) is essential for chondrogenesis. Mutations in and around *SOX9* cause campomelic dysplasia (CD) characterized by skeletal malformations. Although the function of Sox9 in this context is well studied, the mechanisms that regulate Sox9 expression in chondrocytes remain to be elucidated. Here, we have used genome-wide profiling to identify 2 Sox9 enhancers located in a proximal breakpoint cluster responsible for CD. Enhancer activity of E308 (located 308 kb 5′ upstream) and E160 (located 160 kb 5′ upstream) correlated with Sox9 expression levels, and both enhancers showed a synergistic effect in vitro. While single deletions in mice had no apparent effect, simultaneous deletion of both E308 and E160 caused a dwarf phenotype, concomitant with a reduction of Sox9 expression in chondrocytes. Moreover, bone morphogenetic protein 2–dependent chondrocyte differentiation of limb bud mesenchymal cells was severely attenuated in E308/E160 deletion mice. Finally, we found that an open chromatin region upstream of the Sox9 gene was reorganized in the E308/E160 deletion mice to partially compensate for the loss of E308 and E160. In conclusion, our findings reveal a mechanism of Sox9 gene regulation in chondrocytes that might aid in our understanding of the pathophysiology of skeletal disorders.

## Abstract 2

Sox9 gene expression is regulated by two upstream enhancers in chondrocytes, the loss of which promotes a dwarf phenotype in mice.

## Introduction

Cartilage tissue performs a variety of important functions from embryonic to adult life. In the embryonic stage, it forms the cartilage anlagen that serves as a template for the skeleton, and it regulates the longitudinal growth of the bone as growth plate cartilage. This developmental process is called endochondral bone formation, and chondrocytes play essential roles during this process (1). This is exemplified by the fact that genetic dysfunction of chondrocytes causes various types of skeletal dysplasia, including campomeric dysplasia (CD) and achondroplasia.

Chondrocytes originate from skeletal stem cells that arise from mesoderm- and neural crest–derived mesenchymal cells (2). Condensation of these mesenchymal stem cells induces their commitment and differentiation into chondrocytes. It is well established that the transcription factor SRY-related HMG box 9 (Sox9) is a master regulator for chondrocyte differentiation (3). The essential role of Sox9 in skeletal development was first discovered by clinical mutation analysis of patients with CD. The disease is characterized by autosomal sex reversal and skeletal abnormalities, including shortening and bowing of the long bones (4, 5). Genetic studies in mice further revealed that chondrocyte-specific or early-mesenchyme deletion of limb pad–specific Sox9 expression abolishes chondrogenesis and leads to severe defects in skeletal development (6).

The function of Sox9 in chondrocyte differentiation has since been intensively investigated (7). Sox9 controls chondrocyte differentiation and proliferation by directly regulating the transcription of target genes including collagen type II, alpha 1 (*Col2a1*), and aggrecan (*Acan*) through a DNA-binding domain called the HMG box (3, 8, 9). Sox9 does not exert its transcriptional function alone but forms a large complex with several other transcription factors (3, 10). In particular, Sox5 and Sox6, which belong to the same gene family as Sox9, act in concert with Sox9 to promote chondrocyte differentiation (11, 12). Several nuclear proteins including Wwp2 (13), p54nrb (14), and p300 (15) have been proposed to interact with Sox9 during chondrogenesis. These proteins control multiple steps of the gene expression process, including mRNA splicing, chromatin organization, and histone modification. Recent progress of genome-wide analysis uncovered not only 2 distinct modes of action of Sox9 in chondrocytes (16) but also a Sox9-dependent gene regulatory network controlling skeletal development (17).

Despite the progress in understanding Sox9 function during chondrocyte differentiation, the molecular mechanisms underlying Sox9 expression in chondrocytes largely remain unknown. In general, eukaryotic gene expression is regulated by promoters located near the site of transcription initiation and by enhancers that are positioned on average 120 kb away from the transcription start site (TSS) of the target gene (18). Multiple enhancers tend to orchestrate tissue-specific gene expression in a temporospatial manner, and they interact with the promoter by forming a loop-like DNA structure to promote gene transcription (19). Interestingly, recent genome-wide association studies identified mutations in enhancer regions that are associated with human diseases (20). Genetic and epigenetic variation within enhancers affect the binding ability of transcription factors, and chromosomal rearrangements cause the genetic misplacement of enhancers (21), both of which result in the decrease of target gene expression. Indeed, in patients with CD, not only are mutations in the Sox9 coding region observed but also translocations of the noncoding region around the *SOX9* gene locus (22). Previous reports demonstrated that proximal (50~375 kb) and distal (789~932 kb) breakpoint clusters located upstream of the *SOX9* TSS correlate with a skeletal phenotype in patients with CD (23), implying the existence of chondrocyte-specific enhancers in these clusters that are important for Sox9 expression.

Several studies performed genomic conservation analysis and identified chondrocyte-specific Sox9 enhancers at –70 kb and –251 kb upstream of the *Sox9* TSS (24, 25). In addition, Mochizuki et al. identified a single rib cage–specific enhancer located 1 Mb upstream of the *Sox9* TSS using CRISPR/Cas9 technologies (26). Nevertheless, Sox9 gene expression is likely to be regulated by additional enhancers, and the functional cooperation of these multiple enhancers in vivo remains unknown. The identification and characterization of Sox9 enhancers would facilitate our understanding of the mechanism driving Sox9 expression during skeletal development and the pathogenesis of CD. However, this task has been challenging because the regulatory region for Sox9 expression is spread over a 1.5 Mb gene desert, which is expected to include multiple redundant enhancers.

In this study, we performed an unbiased assay for transposase-accessible chromatin sequencing (ATAC-Seq) and ChIP-Seq analysis in mouse primary chondrocytes to screen for Sox9 enhancers in chondrocytes. We identified 2 Sox9 enhancers and demonstrated that synergistic enhancer activity is important for Sox9 expression in vitro and in vivo. Our findings provide insights into Sox9 gene regulation and contribute to a better understanding of the pathophysiology of CD.

## Results

### Genome-wide profiling of open chromatin regions and active enhancer regions in chondrocytes.

**Figure 1:**
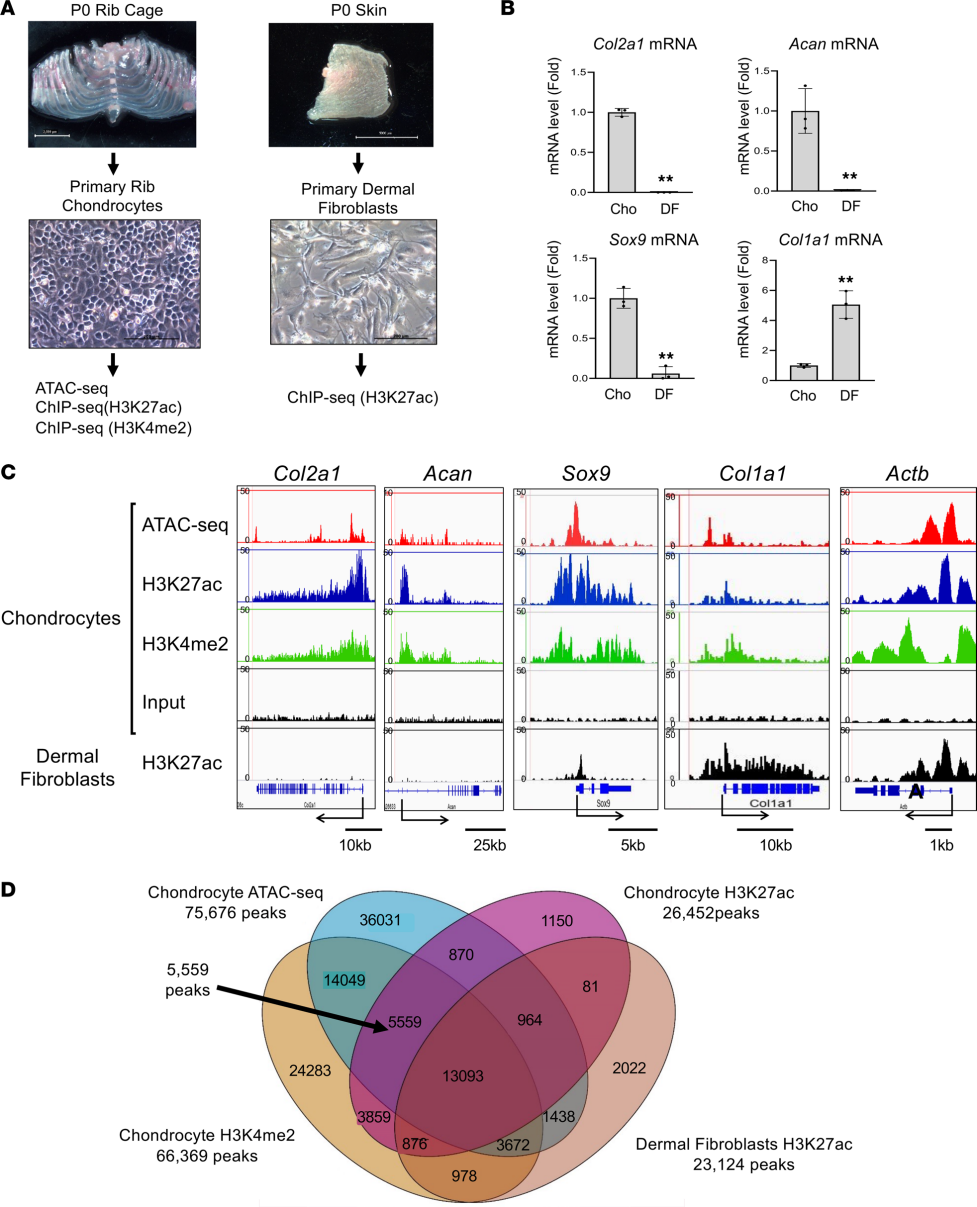
Genome-wide profiling of open chromatin regions and active enhancer in primary chondrocytes. (**A**) Strategy for the genome-wide analysis of primary rib chondrocytes isolated from newborn mice. Primary dermal fibroblasts are used as negative control in the ChIP-Seq analysis. H3K27ac, H3K27 acetylation; H3K4me2, H3K4 dimethylation. (**B**) Total RNA was isolated from primary chondrocytes and dermal fibroblasts and analyzed by RT-qPCR for *Sox9*, *Col2a1*, *Acan*, and *Col1a1*. Data are shown as the mean ± SD (*n* = 3, biologically independent samples). **P < 0.01 (vs. Cho); unpaired Student’s *t* test. Cho, chondrocytes; DF, dermal fibroblasts. (**C**) ATAC-Seq and ChIP-Seq profiles for H3K27ac and H3K4me2 in primary chondrocytes and dermal fibroblasts for *Col2a1*, *Acan*, *Sox9*, *Col1a1*, and *Actb*. (**D**) Venn diagrams showing the numbers and overlap of ATAC-Seq and ChIP-Seq peaks in primary chondrocytes and primary dermal fibroblasts.

To identify enhancers associated with Sox9 gene expression in chondrocytes, we performed genome-wide ATAC-Seq profiling and ChIP-Seq for active enhancer marks in primary rib chondrocytes (Figure 1A). First, we verified high expression of the chondrogenic marker genes *Sox9*, *Col2a1*, and *Acan* in our primary chondrocytes by reverse transcription-quantitative polymerase chain reaction (RT-qPCR) analysis (Figure 1B). Primary dermal fibroblasts that were used as controls showed low expression of these genes but high expression of *Col1a1* (Figure 1B). We then explored open chromatin regions in chondrocytes by ATAC-Seq, and we performed ChIP-Seq analysis using antibodies against H3K27ac (27) and H3K4me2, both active histone marks of promoters and enhancers. Their combination allowed us to reduce false positive predictions based on H3K27ac alone (28). We used H3K27ac ChIP-Seq data from dermal fibroblasts as a negative control.

Initially, we examined the average enrichment of ATAC-Seq and ChIP-Seq signals over genomic regions such as known promoters and enhancers in order to validate our data sets. We found that about 10% (8.2%~13.7%) of ATAC-Seq and ChIP-Seq signals were enriched around promoter regions (≤1,000 bp), whereas the genomic background only showed 1.1% enrichment (Supplemental Figure 1A; supplemental material available online with this article; https://doi.org/10.1172/jci.insight.175486DS1). About 50% of peaks were mapped to ±50–500 kb from the TSS (Supplemental Figure 1B). Enrichment analysis using ngsplot further revealed that ATAC-Seq and ChIP-Seq peaks were enriched at known enhancer regions and at genomic regions around TSSs (Supplemental Figure 2).

We next investigated ATAC-Seq and ChIP-Seq profiles of known chondrocyte genes (Figure 1C). Strong chondrocyte-specific ATAC-Seq and ChIP-Seq peaks were observed in promoter regions and the gene body of chondrogenic genes, including *Col2a1*, *Acan*, and *Sox9* (Figure 1C). ATAC-Seq and ChIP-Seq peaks of these genes were low in dermal fibroblasts, which showed peaks at the fibroblast marker gene *Col1a1* instead (Figure 1C). Venn diagrams of ATAC-Seq peaks and ChIP-Seq peaks identified 5,559 overlapping chondrocyte-specific regions (Figure 1D). GREAT Gene Ontology analysis revealed a correlation of these peaks with chondrocyte function (skeletal system morphogenesis and cartilage development) (Supplemental Figure 2B). Moreover, an investigation with MGI phenotype ontology demonstrated that these peaks were enriched within genes that are associated with abnormal cartilage development and abnormal chondrocyte morphology (Supplemental Figure 3, A and B). These data collectively suggest that our genome-wide profiling data should allow the identification of enhancers associated with chondrocyte-specific gene expression.

### Identification of Sox9 enhancers in chondrocytes.

**Figure 2:**
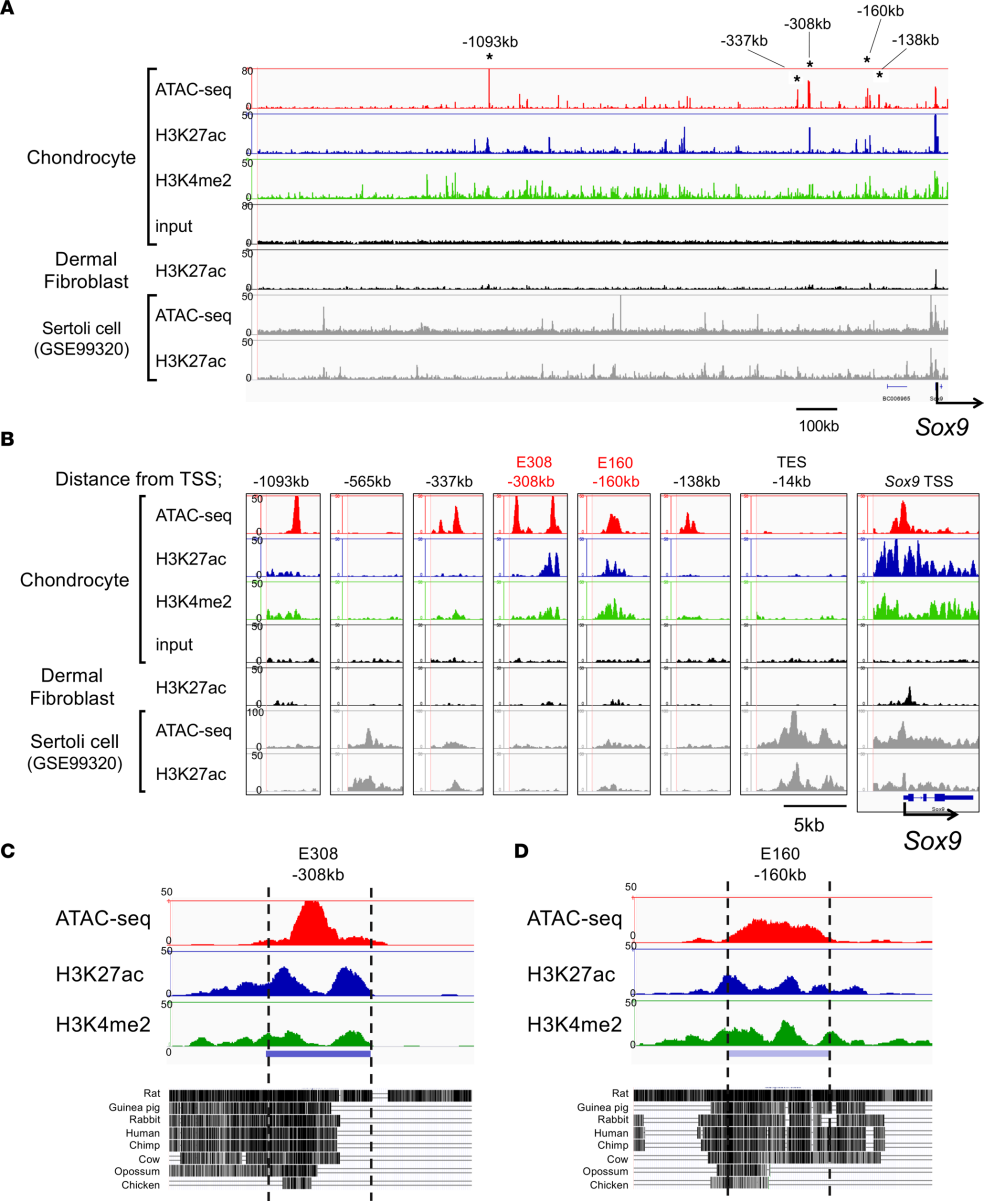
Identification Sox9 enhancers in primary chondrocytes. (**A**) ATAC-Seq and ChIP-Seq profiles of the genomic region 1.5 Mb upstream of mouse Sox9 in chondrocytes, dermal fibroblasts, and Sertoli cells (GSE99320). Asterisks indicate the candidate genomic regions for chondrocyte-specific Sox9 enhancers. (**B**) Profiles of 5 candidate genomic regions of Sox9 enhancer (–1,093 kb, –337 kb, –308 kb, –160 kb, and –138 kb). Previously identified Sox9 enhancers for testis (–565 kb and –14 kb) are also shown. (**C** and **D**) Sequence conservation of –308 kb (**C**) and –160 kb (**D**) in rat, guinea pig, rabbit, human, chimpanzee (Chimp), cow, opossum, and chicken.

We next investigated genomic regions upstream of the Sox9 TSS to search for potential Sox9 enhancers in chondrocytes. We found 5 strong ATAC-Seq peaks (–1,093 kb, –337 kb, –308 kb, –160 kb, and –138 kb) in chondrocytes (Figure 2A). Among these, 2 peaks, one located at –308 kb (referred to as E308, chr11: 112,334,364–112,335,294; 930 bp long) and the other at –160 kb (referred to as E160, chr11: 112,482,190–112,483,273; 1,083 bp long), overlapped with H3K27ac and H3K9me2 peaks. This suggested that E308 and E160 are open and active (Figure 2B). In contrast, these peaks were very weak in dermal fibroblasts (Figure 2B). Replicate ATAC-Seq and ChIP-Seq analysis identified reproducible genomic peaks at the 2 putative enhancer regions in chondrocytes (Supplemental Figure 4, A and B).

To verify that E308 and E160 are specific to chondrocytes, we used public ATAC-Seq and H3K27ac ChIP-Seq data sets (National Center for Biotechnology Gene Expression Omnibus GSE99320) of Sertoli cells. These cells promote testis development and are known to have high levels of Sox9 expression (29). A comparison with our data sets in chondrocytes verified that testis-specific Sox9 enhancers including –565 kb (29) and testis-specific enhancer of Sox9, located at –13 kb (30), are open and active in Sertoli cells but not in chondrocytes. In contrast, peaks of E308 and E160 were very low in Sertoli cells (Figure 2, A and B, lower panel). Moreover, the genomic regions of E308 and E160 showed a high level of sequence conservation among vertebrates including rabbit, human, opossum, and chicken (Figure 2, C and D). The homologous regions of E160 and E308 in the human genome correspond to –327 kb (Chr17: 69,971,433–69,972,581) and –144 kb (Chr17: 69,971,433–69,972,581) from the *SOX9* TSS. Both enhancers are located within a proximal breakpoint cluster (–375~–50 kb), which is associated with severe CD (22). SOX9 promoter-anchored Capture-C assays (31) further revealed that these homologous regions of E160 and E308 contact the *SOX9* promoter in human cranial neural crest cells, which show high expression of *SOX9* (Supplemental Figure 5). Overall, these data suggest that E308 and E160 are potential Sox9 enhancers in chondrocytes.

### Enhancer activity of E308 and E160 in vitro.

**Figure 3:**
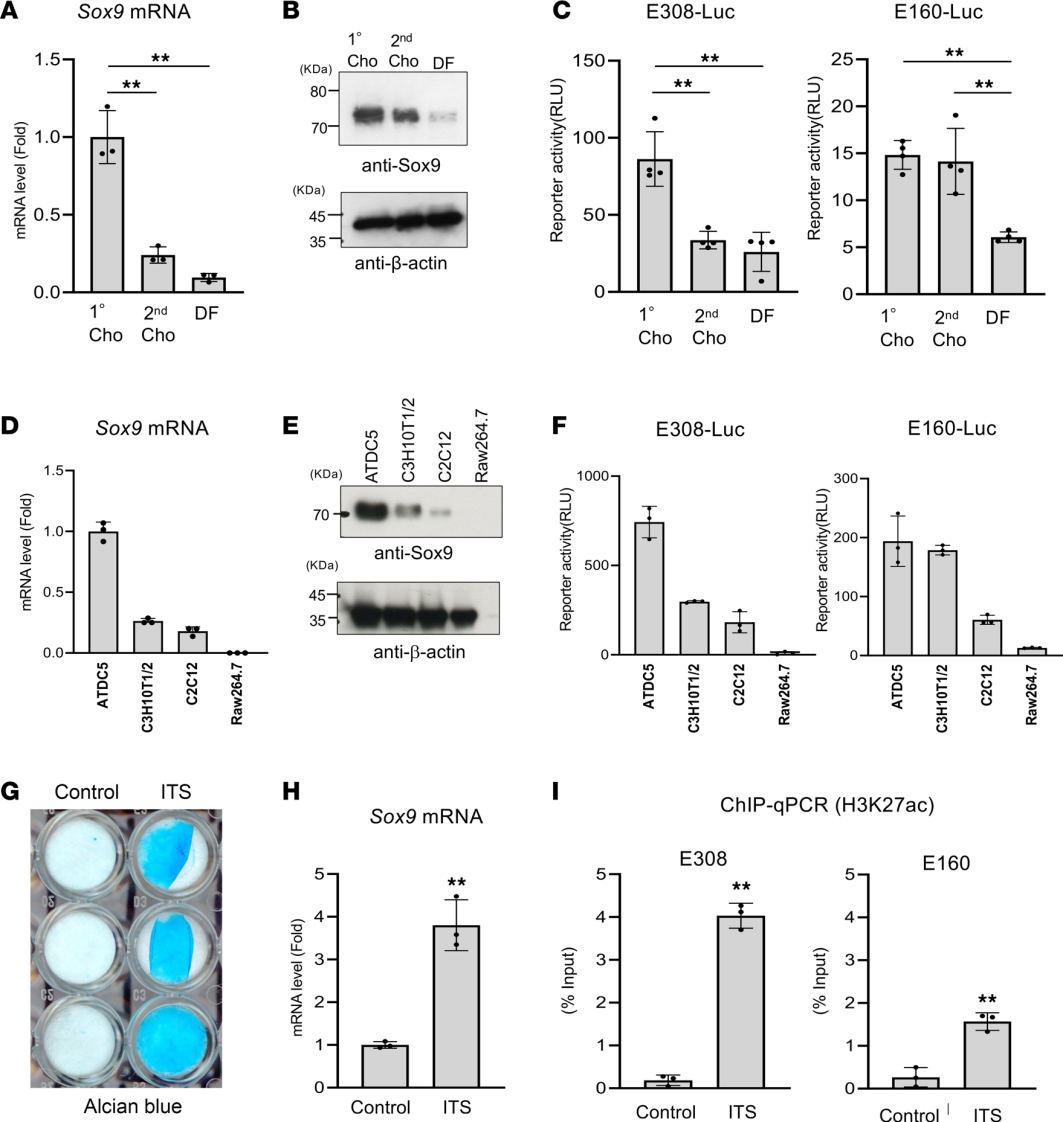
Correlation of identified enhancer activity with *Sox9* expression. (**A**) Comparison of *Sox9* mRNA levels in primary chondrocytes (1°Cho), 2nd passage chondrocytes (2nd Cho), and dermal fibroblasts (DF) using RT-qPCR. Data are shown as the mean ± SD (*n* = 3, biologically independent samples). **P < 0.01; 1-way ANOVA followed by Tukey’s multiple-comparison test. (**B**) Comparison of Sox9 protein between 1°Cho, 2nd Cho, and DF by Western blot. (**C**) Luciferase reporter plasmids that include the E160 or E308 enhancer and a Sox9 minimal promoter were transfected into 1°Cho, 2nd Cho, and DF cells. Luciferase activity was measured 48 hours after transfection. Data are shown as the mean ± SD (*n* = 4, biologically independent samples). **P < 0.01, 1-way ANOVA followed by Tukey’s multiple-comparison test. (**D**) Comparison of *Sox9* mRNA in different cell lines by RT-qPCR. Data are shown as the mean ± SD (*n* = 3, biologically independent samples). (**E**) Comparison of Sox9 protein in different cell lines by Western blot. (**F**) Luciferase reporter plasmids as in **C** were transfected into cell lines. Luciferase activity was measured 48 hours after transfection. Data are shown as the mean ± SD (*n* = 4, biologically independent samples). (**G**) Chondrocyte differentiation of ATDC5 cells. ATDC5 cells were cultured with or without insulin, transferrin, and selenium (ITS) for 21 days and then stained with Alcian blue. (**H**) Total RNA isolated from ATDC5 cells was analyzed by RT-qPCR for Sox9 gene expression. Data are shown as the mean ± SD (*n* = 3, biologically independent samples). **P < 0.01 (vs. Control). (**I**) ChIP-qPCR analysis of E308 and E160 in ATDC5 cells treated with or without ITS. Sonicated chromatin isolated from ATDC5 cells was immunoprecipitated with anti-H3K27ac antibody and quantified using specific primers for E308 and E160. Data are shown as the mean ± SD (*n* = 3, biologically independent samples). **P < 0.01 (vs. Control); unpaired Student’s *t* test.

We next examined whether the enhancer activity of E308 and E160 correlates with *Sox9* gene expression in cell culture. We therefore created reporter constructs in which the potential enhancer region (E308 or E160) was cloned upstream of a Sox9 minimal promoter (–100~+10 bp) and fused to a luciferase reporter gene. We first investigated enhancer activity of E308 and E160 in primary chondrocytes, second passage chondrocytes, and dermal fibroblasts. RT-qPCR and Western blot analysis showed that Sox9 expression was highest in primary chondrocytes and decreased in second passage chondrocytes (Figure 3, A and B). Dermal fibroblasts showed low expression of Sox9 mRNA and protein (Figure 3, A and B). We observed that the activity of E308 based on relative luciferase levels correlated with *Sox9* gene expression (Figure 3C). In contrast, E160 enhancer activity was similar in second passage chondrocytes compared to primary chondrocytes, though Sox9 expression was lower in these cells (Figure 3C).

Next, we compared the enhancer activity in 4 cell lines with varying levels of Sox9 expression (ATDC5, C3H10T1/2, C2C12, and Raw264.7). Mouse chondrogenic ATDC5 cells have high expression of Sox9. Mouse mesenchymal C3H10T1/2 cells, which can be differentiated into chondrocytes and C2C12 myoblasts, weakly expressed Sox9, and Raw264.7 cells did not express Sox9 (Figure 3, D and E). The enhancer activity of E308 strongly correlated with Sox9 expression in all cell lines (Figure 3F). Consistent with the data in primary and second passage chondrocytes, E160 showed –similar activity in chondrogenic ATDC5 and C3H10T1/2 cells (Figure 3F). E308 and E160 activity was very low in Raw264.7 cells (Figure 3F).

We next tested whether E308 and E160 activity increases during chondrocyte differentiation. Therefore, we treated ATDC5 cells with a cocktail of insulin, transferrin, and selenium (ITS), which triggered chondrocyte differentiation as indicated by increased matrix production assayed by Alcian blue staining and increased *Sox9* gene expression (Figure 3, G and H). ChIP-qPCR using the anti-H3K27ac antibody revealed concomitant enrichment of H3K27ac in E308 and E160 (Figure 3I). These data suggest that E308 and E160 enhancer activity correlates with Sox9 expression in chondrocytes.

### Synergistic enhancer activity of E160 and E308 in chondrocytes.

**Figure 4:**
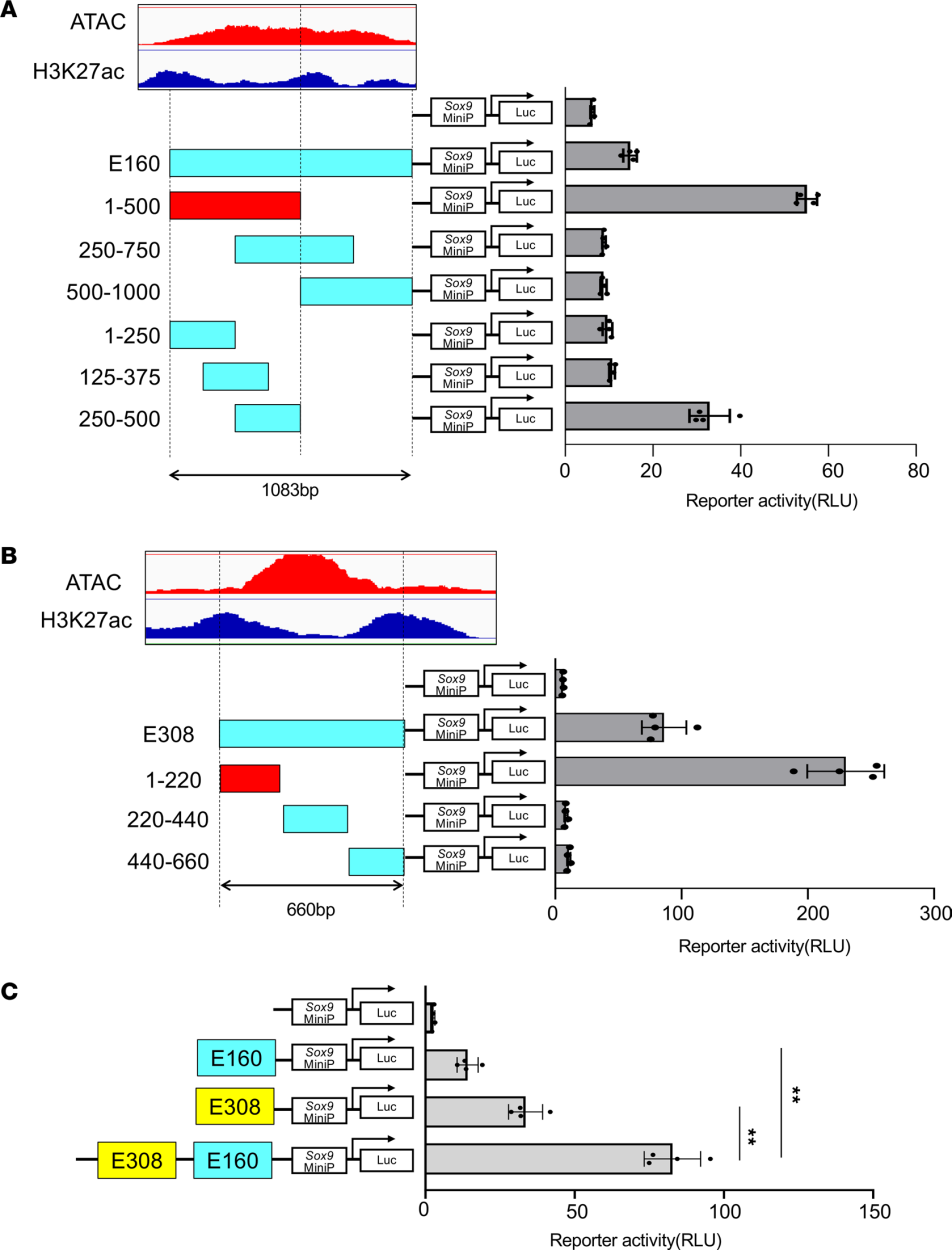
Reporter assay of E160 and E308 enhancer in primary chondrocytes. (**A**–**C**) Luciferase reporter constructs of deletions of E160 (**A**), deletions of E308 (**B**), or tandem repeats of E160 and E308 (**C**) fused to the Sox9 minimal promoter were transfected into primary chondrocytes. Luciferase activities were measured at 48 hours after transfection. Data are shown as the mean ± SD (*n* = 4, biologically independent samples). **P < 0.01, 1-way ANOVA followed by Tukey’s multiple-comparison test.

To further define the regions important for E160 and E308 enhancer activity in chondrocytes, we divided the genomic region covered by the ATAC-Seq peaks and generated various reporter constructs (Figure 4, A and B). We used the full-length region of E160 and a partial region (660 bp in length) of E308, which showed high conservation between species (Figure 2C and Figure 4B). These reporter constructs were transfected into primary chondrocytes and subjected to reporter assays. We found that 500 bp on the 5′ side of E160 and 220 bp on the 5′ side of E308 were important for enhancer activity (Figure 4, A and B).

Because cooperative transcriptional regulation by multiple enhancers has been reported (32), we also generated a construct that combined both E160 and E308 and examined if both enhancers led to synergistic gene activation. We found that the tandem arrangement of E160 and E308 markedly increased luciferase expression compared with E160 and E308 alone (Figure 4C). These results suggest that E160 and E308 act cooperatively to promote *Sox9* gene expression.

### Regulation of Sox9 enhancer activity by paired like homeodomain 1.

**Figure 5:**
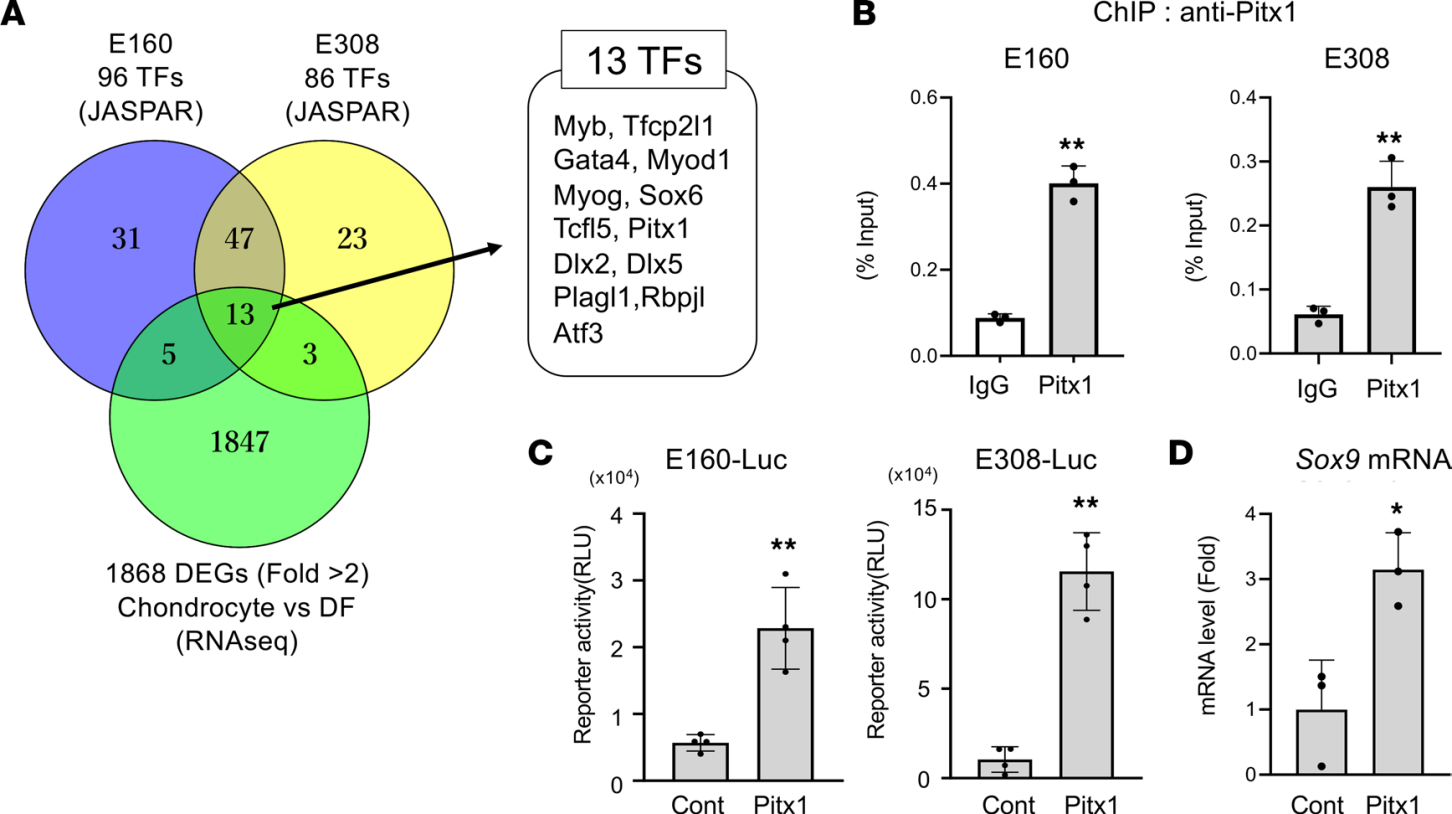
Regulation of Sox9 enhancer activity and Sox9 expression by Pitx1. (**A**) Venn diagram of motif analysis and differentially expressed genes (DEGs) between chondrocytes and dermal fibroblasts (DF) based on RNA-Seq data. TFs, transcription factors. (**B**) ChIP assay using IgG and anti-Pitx1 antibodies. Binding of Pitx1 to E160 and E308 in primary chondrocytes was examined by qPCR using specific primer pairs. Data are shown as the mean ± SD (*n* = 3, technical triplicates). **P < 0.01 (vs. IgG); Student’s *t* test. (**C**) HEK293 cells were transfected with reporter plasmids together with or without Pitx1. Luciferase activities were measured at 48 hours after transfection. Data are shown as the mean ± SD (*n* = 4, biologically independent samples). **P < 0.01 (vs. Control); Student’s *t* test. (**D**) Limb bud mesenchymal cells were infected with the control (Cont) or Flag-Pitx1 adenovirus, and Sox9 expression was analyzed by RT-qPCR. Data are shown as fold-changes normalized to Cont (mean ± SD) (*n* = 3, biologically independent samples). **P < 0.05 (vs. Cont); Student’s *t* test.

In order to identify transcription factors that bind and promote the enhancer activity of E308 and E160, we used JASPAR (33) to perform sequence motif analyses of E308(1–220) and E160(1–500), the 2 enhancer regions that elicit high luciferase activity as discussed above (Figure 4, A and B). We identified 96 motifs in E160 and 86 motifs in E308 (Figure 5A and Supplemental Table 5). To narrow down candidate transcription factors based on their expression, we conducted RNA-Seq analysis in primary chondrocytes and primary dermal fibroblasts. We identified 1,868 genes that showed over 2-fold higher expression in chondrocytes compared with primary dermal fibroblasts (Figure 5A and Supplemental Table 6). There were 13 transcription factors whose binding motif was present in both E308(1–220) and E160(1–500) (Figure 5A).

Among these genes, we decided to focus on paired like homeodomain 1 (Pitx1), because this transcription factor is known to regulate chondrogenesis and limb development (34, 35) and *PITX1* mutations in humans cause lower limb malformation (36). However, the precise role of Pitx1 during chondrogenesis is not still defined. ChIP-qPCR analysis using an antibody against Pitx1 demonstrated that Pitx1 interacted with E160 and E308 (Figure 5B). Moreover, Pitx1 promoted the luciferase activity of E160-Luc and E308-Luc (Figure 5C), and overexpression of Pitx1 increased Sox9 expression in limb bud mesenchymal cells (Figure 5D).

We identified 2 predicted binding motifs of Pitx1 in E160 (P1 and P2 in Supplemental Figure 6A) and 1 motif of Pitx1 in E308 (P3 in Supplemental Figure 7A) in the JASPAR database. Pull-down assays using biotinylated DNA demonstrated that Pitx1 directly bound to P1 and P3. This association was abolished by an excess of an unlabeled competitive probe (Supplemental Figure 6B and Supplemental Figure 7B). Moreover, while Pitx1 promoted the luciferase activity of E160-Luc and E308-Luc (Figure 5C), mutation of the P1 and P3 Pitx1 binding motif abrogated Pitx1-dependent upregulation of E160-Luc and E308-Luc (Supplemental Figure 6D and Supplemental Figure 7C). In contrast, Pitx1 failed to interact with the P2 motif, and mutation of the P2 motif did not alter the upregulation of E160-Luc induced by Pitx1 (Supplemental Figure 6, C and E). These data indicate that Pitx directly binds to E308 and E160 and promotes Sox9 expression.

We next compared the expression pattern of Sox9 and Pitx1 using a single-cell RNA-Seq data set of the E11.5 limb bud (National Center for Biotechnology Gene Expression Omnibus GSE142425) (37). Within this data set, we identified *Sox9*^+^*Col2a1*^+^*Col1a1*^–^ chondrocytes (cluster 0, 1), *Sox9*^+^*Col2a1*^+^*Col1a1*^+^ osteo-chondroprogenitor cells (38) (cluster 3, 4), *Cdh5*^+^ endothelial cells (cluster 2), and *Krt14*^+^ keratinocytes (cluster 5, 6) (Supplemental Figure 8, A and B). Pitx1 was expressed in chondrocytes and osteo-chondroprogenitor cells, which express Sox9, while other candidate transcription factors were not detected in these cell clusters (Supplemental Figure 8C). Of note, the expression pattern of Pitx1 overlapped with that of Sox9 (Supplemental Figure 8D). These data collectively suggest that Pitx1 regulates Sox9 expression through E308 and E160 in chondrocytes and progenitors.

### In vivo relevance of E308 and E160 in Sox9 expression and skeletal development.

To determine the relevance of E308 and E160 in *Sox9* gene regulation and skeletal development in vivo, we generated mice that lacked the enhancer regions using CRISPR/Cas9-based genome editing (Supplemental Figures 9 and 10). However, homozygous deletion of E160 (E160^Δ/Δ^) or of E308 (E308^Δ/Δ^) alone did not cause an obvious skeletal phenotype as assessed by skeletal preparation and histological analysis of the tibia (Supplemental Figure 9, C–E, and Supplemental Figure 10, C–E). In addition, expression levels of *Sox9*, *Col2a1*, *Sox5*, and *Sox6* in forelimbs of E15 embryos were similar to wild-type mice in both mutants (Supplemental Figure 9F and Supplemental Figure 10F). Both E160^Δ/Δ^ and E308^Δ/Δ^ mice grew normally and were fertile.

**Figure 6:**
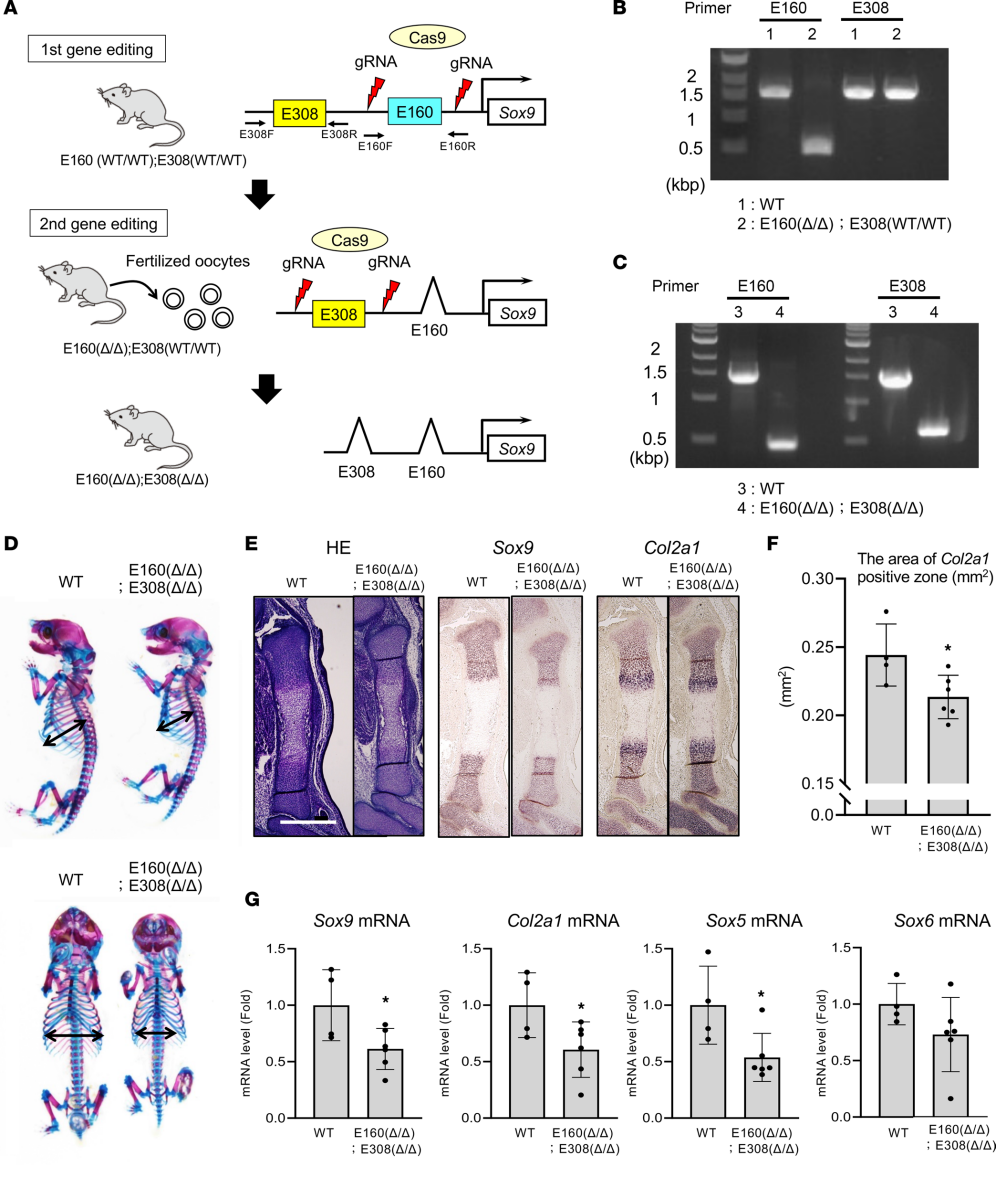
Simultaneous deletion of E160 and E308 in mice causes dwarf phenotype with reduced Sox9 expression. (**A**) Schematic model of our 2-step genome-editing approach to delete E160 and E308. Fertilized oocytes were collected from E160^Δ/Δ^ E308^WT/WT^ mice, and the E308 genomic region was deleted in a second round of genome editing. (**B** and **C**) Genotyping of E160^Δ/Δ^ E308^WT/WT^ (**B**) and E160^Δ/Δ^ E308^Δ/Δ^ (**C**) mice. (**D**) Image of Alcian blue/alizarin red S–stained skeletal preparations of newborn WT and E160^Δ/Δ^ E308^Δ/Δ^ littermate mice. (**E**) Sections of tibiae from WT and E160^Δ/Δ^ E308^Δ/Δ^ littermate E15.0 embryos were stained with hematoxylin and eosin (HE); RNA in situ hybridization analysis using antisense probes against Sox9 and Col2a1. Scale bar: 500 μm. (**F**) Quantitative analysis of Col2a1-positive expression in E15.0 WT and E160^Δ/Δ^ E308^Δ/Δ^ mouse embryos. Data are shown as the mean ± SD. WT: *n* = 4 animals. E160^Δ/Δ^ E308^Δ/Δ^: *n* = 6 animals. *P < 0.05 (vs. WT); Student’s *t* test. (**G**) Total RNA was isolated from forelimbs of E15.0 WT E160^Δ/Δ^ E308^Δ/Δ^ mouse embryos and analyzed by RT-qPCR. Data are shown as fold-changes normalized to WT (mean ± SD). WT: *n* = 4 animals. E160^Δ/Δ^; E308^Δ/Δ^: *n* = 6 animals. *P < 0.05 (vs. WT). Student’s *t* test.

Enhancers are known to show redundancy in the regulation of complex spatiotemporal gene expression (39), and our reporter assays demonstrated that E160 and E308 showed synergistic activity (Figure 4C). Therefore, we hypothesized that simultaneous deletion of E160 and E308 might affect Sox9 expression and skeletal development in vivo. We thus devised a 2-step genome-editing approach to generate mice with a double deletion of E160 and E308 because E160 and E308 are located relatively close together on the same chromosome (Figure 6A). We confirmed the simultaneous deletion of E160 and E308 by genomic PCR (Figure 6, B and C). These double-deletion mice (E160^Δ/Δ^ E308^Δ/Δ^) showed a dwarf phenotype with a smaller rib cage (Figure 6D). Histological analysis demonstrated impaired growth of the tibia (Figure 6E). In addition, the area of *Col2a1*-positive chondrocytes was substantially smaller than that of wild-type mice (Figure 6F). Moreover, mRNA isolated from the forelimbs of E15.0 E160^Δ/Δ^ E308^Δ/Δ^ embryos showed a significant reduction of *Sox9*, *Col2a1*, and *Sox5* expression compared with that of wild-type mice (Figure 6G). These data indicated that E160 and E308 play important roles in skeletal development by regulating Sox9 expression.

**Figure 7:**
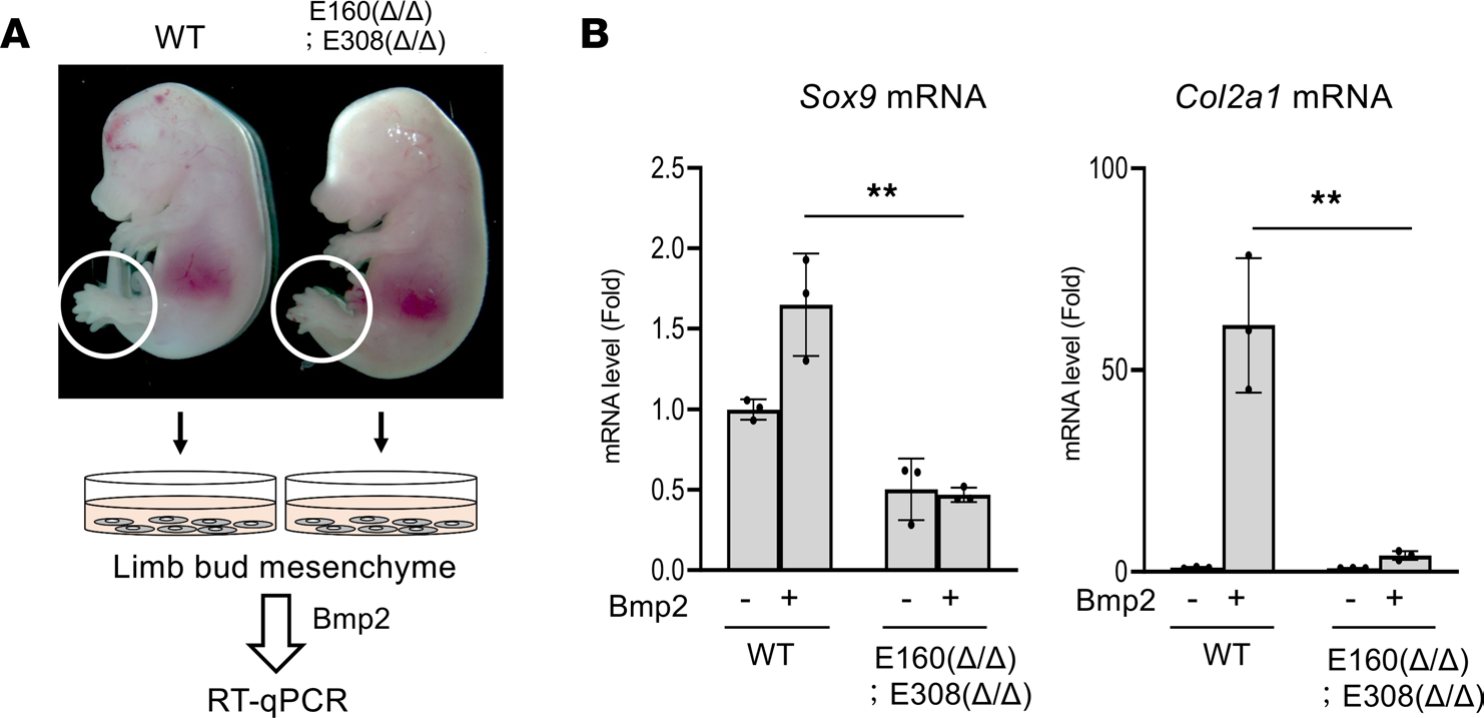
Impaired chondrogenesis in E160 and E308 deletion mice. (**A**) Schematic of the method to examine chondrogenesis of limb bud mesenchymal cells isolated from E12.5 WT and E160^Δ/Δ^ E308^Δ/Δ^ mice. (**B**) Limb bud mesenchymal cells isolated from WT mice and E160^Δ/Δ^ E308^Δ/Δ^ littermates were cultured with or without BMP2 for 7 days. Total RNA was isolated and *Sox9* and *Col2a1* mRNA expression was determined by RT-qPCR. The RNA level is indicated as the fold-increase compared with the WT control (mean ± SD, *n* = 3, biologically independent samples). **P < 0.01; 1-way ANOVA followed by the Tukey test.

We next tested whether chondrocyte differentiation was impaired in E160^Δ/Δ^ E308^Δ/Δ^ mice. We therefore isolated limb bud mesenchymal cells from wild-type and E160^Δ/Δ^ E308^Δ/Δ^ E12.5 embryos and investigated bone morphogenetic protein 2–dependent (BMP2-dependent) chondrocyte differentiation (Figure 7A). We observed that BMP2-dependent *Sox9* induction was not observed in limb bud mesenchymal cells isolated from E160^Δ/Δ^ E308^Δ/Δ^ mice (Figure 7B). In addition, there was a striking reduction of *Col2a1* expression in E160^Δ/Δ^ E308^Δ/Δ^. Overexpression of Pitx1 failed to promote Sox9 expression in primary limb bud cells isolated from E160^Δ/Δ^ E308^Δ/Δ^ embryos (Supplemental Figure 11). These data suggest that the dwarf phenotype of the double-deletion mice is caused at least in part by a decrease of *Sox9* gene expression and impaired chondrogenesis (Figure 7B).

### Chromatin reorganization in double-deletion mice.

**Figure 8:**
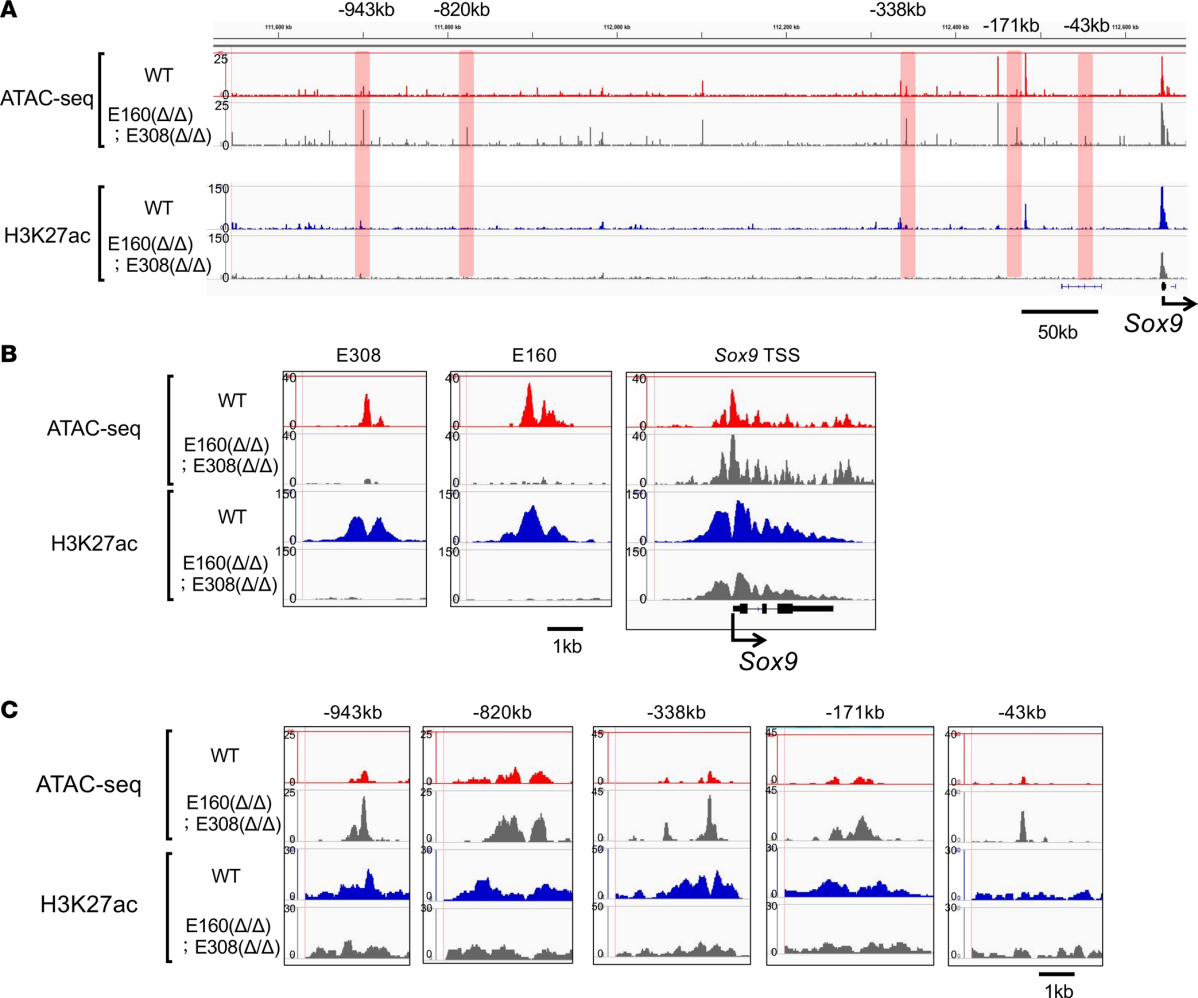
ATAC-Seq and H3K27ac ChIP-Seq signals in WT and E160^Δ/Δ^ E308^Δ/Δ^ chondrocytes. (**A**) ATAC-Seq and ChIP-Seq profiles within a 1 Mb region upstream of the mouse Sox9 gene in primary chondrocytes of WT and E160^Δ/Δ^ E308^Δ/Δ^ littermate mice. Light red shading highlights the strong peaks of E160^Δ/Δ^ E308^Δ/Δ^ mice compared with those of WT mice. (**B**) Specific profiles of E308, E160, and Sox9 TSSs. Note that peaks of E308 and E160 were not detectable in of E160^Δ/Δ^ E308^Δ/Δ^. (**C**) Changes in ATAC-Seq and H3K27 peaks in primary chondrocytes isolated from WT mice and E160^Δ/Δ^ E308^Δ/Δ^ littermates.

Although the double-deletion mice showed impaired chondrogenesis (Figure 7B), the expression level of *Sox9* was reduced to only about 60% compared with wild-type mice, and the observed skeletal phenotype was relatively mild (Figure 6). Therefore, other enhancers are likely to compensate for the loss of E160 and E308 in E160^Δ/Δ^ E308^Δ/Δ^ mice. To test this hypothesis, we isolated primary chondrocytes from WT and E160^Δ/Δ^ E308^Δ/Δ^ mice and performed ATAC-Seq and ChIP-Seq (Figure 8A). We verified that E160 and E308 were active in wild-type mice but not detectable in the genome of E160^Δ/Δ^ E308^Δ/Δ^ mice (Figure 8B). Of note, we identified peaks at several genomic regions (–943 kb, –820 kb, –338 kb, –171 kb, and –43 kb) in E160^Δ/Δ^ E308^Δ/Δ^ mice that were stronger than those of wild-type mice (Figure 8C). These data suggest that the chromatin structure of this region might be reorganized to partially compensate for the loss of E160 and E308 enhancers in double-deletion mice, which helps to maintain a certain level of Sox9 expression in chondrocytes.

## Discussion

In this study, we identified 2 enhancers important for Sox9 expression in chondrocytes, located 160 kb and 308 kb upstream of the *Sox9* TSS. Simultaneous deletion of these enhancers in mice caused a decrease in Sox9 gene expression in chondrocytes and impaired skeletal development. Our findings provide insights into the mechanism of Sox9 gene regulation in chondrocytes and contribute to a better understanding of the molecular pathogenesis underlying CD.

CD is caused not only by loss-of-function mutations in the *SOX9* coding region but also by translocations and deletions in the noncoding region around the *SOX9* gene (22). The clinical phenotype depends on the location of the mutated genomic region. Mutation in a proximal breakpoint cluster (–375~–50 kb) is associated with the most severe skeletal phenotype, while mutations in a distal breakpoint cluster (–932 kb to –350 kb) show mild skeletal symptoms or no limb curvature (22). It should be noted that the regions homologous to E308 and E160 in the human genome lie –322 kb and –144 kb upstream of the SOX9 TSS, respectively, and both are located within the proximal breakpoint cluster. Moreover, SOX9 promoter-anchored Capture-C assays (31) revealed that these regions contact SOX9 promoter in human cranial neural crest cells (Supplemental Figure 5). Although physical interaction between E160, E308, and *Sox9* promoter was not examined in chondrocytes, these data imply that the decreased expression of *Sox9* in double-deletion mice is partially caused by the loss of long-range contacts between E160, E308, and *Sox9* promoter and that these enhancers might be relevant to the abnormal skeletal development observed in CD. To support this notion, mutations in the far-upstream enhancers, which contact the SOX9 promoter, perturb SOX9 expression during craniofacial development and cause Pierre Robin sequence (31). There are multiple SNPs in genomic regions that correspond to E160 and E308 in humans (E160: rs27023009, rs27023010, rs27023011, rs27023012, rs27023013, rs27023014, E308: rs27002140, rs27002141 rs27002141). It will therefore be interesting to examine the association between these SNPs and skeletal disorders.

Conventional methods to identify enhancers include conservation analysis, in vitro reporter assays, and generating and analyzing LacZ-reporter mice. These methods have been widely used, e.g., for the identification and characterization of *Sox9*, *Col2a1*, and *Acan* enhancers (40–42). However, the fact that multiple *Sox9* enhancers are located in a gene desert spread over a 1.5 Mb genomic region (22, 43) makes it challenging to identify all relevant enhancers that regulate Sox9 gene expression in a cell type–specific context via these methods. Therefore, we used an alternative approach based on ATAC-Seq analysis to investigate open chromatin regions, combined with ChIP-Seq for active enhancer marks (H3K27ac, H3K4me2). Focusing on the 1.5 Mb genomic region upstream of the Sox9 TSS, we identified 2 enhancers that are important for Sox9 expression in chondrocytes. We further demonstrated the importance of these enhancers in skeletal development using genome editing (Figures 6 and 7). Although we focused on Sox9 in this study, the same experimental approach can be applied to other chondrocyte-specific genes, such as *Ihh* and *Acan*. Indeed, we have identified over 5,000 chondrocyte-specific enhancers by unbiased genome-wide analysis (Figure 1D). Further studies of these enhancers would deepen our understanding of the molecular mechanisms underlying chondrocyte differentiation.

Transcription factor binding motif analyses of our ATAC-Seq and ChIP-Seq data also enable the identification of transcription factors, whose binding motifs are enriched in chondrocyte enhancers. This approach has been successfully applied in the past. For example, Waki et al. extracted and analyzed adipocyte-specific enhancer regions using a motif database and found Nf1 as a transcription factor that regulates adipocyte differentiation (44). Here, we used a combination of motif analysis and RNA-Seq to identify several transcription factors that might regulate E160 and E308, the 2 chondrocyte-specific enhancers we identified (Figure 5). We focused on Pitx1, a transcription factor, which plays important roles in limb development by regulating chondrogenesis (34, 35). Moreover, in humans, dominant negative mutations of *PITX1* cause lower limb malformations (36). Combined with our finding that PITX binds to and activates E160 and E308 in cell-based assays, these data support the notion that the Pitx1/Sox9 axis plays important roles in chondrocyte differentiation during skeletal development. Interestingly, it has been reported that Pitx1 promotes Sox9 expression during early astrocyte differentiation through a unique Pitx1 binding motif located –200 bp upstream of the Sox9 TSS (45). This suggests that Pitx1 regulates tissue-specific Sox9 expression through different enhancers. In addition to Pitx1, our motif analysis also indicates that Sox6 binds to E160 and E308. Interestingly, Yao et al. reported that Sox9 binds to its own enhancers, dependent on Sox5 and Sox6 (25). While we do not detect a Sox9 binding motif in our computational motif analysis, it will be interesting to examine whether Sox9 can activate E160 and E308, perhaps in conjunction with Sox6. Our genome-wide next-generation sequencing data sets will also be a valuable resource to search for additional transcription factors that regulate chondrocyte differentiation.

We generated mice that carry a simultaneous deletion of E160 and E308 and show that these enhancers are important in skeletal development. Although we observed no significant changes in Sox9 expression upon deletion of either E160 or E308 alone, mice carrying the double deletion show decreased *Sox9* gene expression in chondrocytes and a dwarf phenotype. Moreover, we identified ATAC-Seq peaks at several genomic regions in double-deletion mice that are stronger than those of WT mice (Figure 8). These data suggest redundancy of the 2 enhancers for Sox9 in chondrocytes, and similar redundancies have been reported for other genes, including *Ihh* (46), *Shh* (47), and *Hox* (39). These reports and our findings indicate that enhancer redundancy is the fundamental backup system and necessary to prevent the misregulation of important developmental regulators upon disruptions of single enhancers.

In an earlier study, Yao et al. used conservation analysis to identify 3 Sox9 enhancers located –250 kb, –195 kb, and –84 kb from the TSS that are important during chondrocyte differentiation. In this case, the 3 enhancers played different, nonredundant roles: –250 kb acted during mesenchymal cell aggregation, –195 kb specifically in proliferating chondrocytes, and –84 kb in all differentiation stages of chondrocytes (25). Interestingly, the enhancer activity of E160 is equivalent in primary and second passage chondrocytes, while E308 showed higher activity in primary chondrocytes compared with second passage chondrocytes (Figure 3C). These data suggest that E308 functions to regulate Sox9 expression in early and mature chondrocytes, while E160 plays a role in early chondrogenesis. In addition, limb bud mesenchymal cells derived from mice lacking E160 and E308 showed a defect in BMP2-induced chondrocyte differentiation (Figure 7), implying that E160 and E308 might be BMP2-responsive enhancers in early chondrocytes. Future studies should clarify the timing of E160 and E308 function and the regulatory mechanism of their activation.

In conclusion, we uncover a mechanism of Sox9 gene regulation during skeletal development. Our findings increase our understanding of the molecular mechanisms of endochondral bone formation, which might be relevant for the pathogenesis of inherited skeletal disorders, such as CD.

## Methods

### Sex as a biological variable.

Sex was not considered as a biological variable because male and female mice showed similar results in skeletal development at embryonic stage.

### Cell culture and reagents.

The mouse fibroblast-like cell line C3H10T1/2 (RCB0247), mouse teratocarcinoma cell line ATDC5 (RCB0565), mouse myoblast cell line C2C12 (RCB0987), and human cell line HEK293 (RCS1637) were provided by the RIKEN BRC through the National BioResource Project of the MEXT/AMED, Japan. Mouse macrophage cell line Raw264.7 was purchased from ATCC. These cells were cultured at 37°C in a humidified 5% CO_2_ incubator with DMEM (MilliporeSigma) containing 10% fetal bovine serum (FBS) and a 1:1 mixture of DMEM and Ham’s F12 medium (MilliporeSigma) containing 10% FBS. ITS (Roche) was used to induce chondrocyte differentiation in ATDC5 cells.

Primary chondrocytes were isolated in accordance with a protocol described by Gartland et al. (48). Briefly, rib cartilage was dissected from newborn mice and initially digested with 2.5 mg/mL Collagenase Type 2 (Worthington Biochemical Corporation) for 1 hour at 37°C to remove soft tissues. After washing ribs with PBS, ribs were incubated with 2.5 mg/mL Collagenase Type 2 for an extra 6 hours at 37°C to isolate chondrocytes. Primary chondrocytes were collected by centrifugation at 20°C, for 5 minutes, at 500*g*, and resuspended with DMEM containing 10% FBS and antibiotics. Cells within 2 passages were used for experiments as primary chondrocytes.

Primary dermal fibroblasts were isolated from the dorsal skin of newborn mice. Briefly, dorsal skin was surgically collected using scissors and digested with 0.5 mg/mL collagenase (WAKO) at 4°C for 48 hours. After removing the dermis, the skin was dispersed into single-cell suspension by mechanical pipetting. Cells were collected by centrifugation at 20°C, for 5 minutes, at 500*g*, and resuspended with DMEM containing 10% FBS.

### RT-qPCR.

Cells were washed with cold PBS, and total RNA was isolated using a NucleoSpin RNA Plus kit (TAKARA). cDNA was synthesized using ReverTra Ace qPCR RT Master Mix (TOYOBO). cDNA was amplified with EagleTaq Universal Master Mix (ROX) using a StepOnePlus Real-Time PCR System (Applied Biosystems). Primers and TaqMan probes used for cDNA amplification are listed in Supplemental Table 1. The mRNA expression was normalized to β-actin expression levels.

### ATAC-Seq.

Fragmentation and amplification of ATAC-Seq libraries were constructed according to Buenrostro et al. (49). Primary chondrocytes were collected and resuspended in cold PBS. Approximately 50,000 cells were lysed using lysis buffer (10 mM Tris-HCl at pH 7.4, 10 mM NaCl, 3 mM MgCl_2_, and 0.1% IGEPAL CA-630), and a transposition reaction was performed with the Tn5 Transposase (Illumina catalog FC121-1030) at 37°C for 30 minutes. The reaction liquid was purified with NucleoSpin Gel and PCR Clean-up (MACHEREY-NAGEL). A total of 5 cycles of PCR with PCR primers (Ad1_noMX: 5′-AATGATACGGCGACCACCGAGATCTACACTCGTCGGCAGCGTCAGATGTG-3′ and Ad2.1_TAAGGCGA: 5′-CAAGCAGAAGACGGCATACGAGATTCGCCTTAGTCTCGTGGGCTCGGAGATGT-3′) were conducted using 1× NEBNext PCR Master Mix (New England Biolabs). An additional number of PCR cycles was determined by qPCR of the partly amplified products. The PCR products were purified with NucleoSpin Gel and the PCR Clean-up kit according to the manufacturer’s protocol. Paired-end sequencing (100 bp) was performed on the HiSeq sequencer (Illumina). Quality control and preprocessing of ATAC-Seq reads were performed using fastp (50). ATAC-Seq reads were mapped to the mm9 reference sequence using Bowtie2 (ver. 2.4.4) (51), and duplicate reads were removed with Picard (ver. 2.26.9, https://broadinstitute.github.io/picard/). ATAC-Seq peaks were detected using MACS2 (ver.2.2.4) (52) with the default setting (*q* value, 0.05) and visualized with IGV (ver.2.13.0). Two biological replicates were analyzed for ATAC-Seq.

### ChIP and ChIP-Seq.

Primary chondrocytes and primary dermal fibroblasts were cross-linked with 1% methanol-free formaldehyde for 5 minutes. Cross-linking was quenched with 1 M glycine for 5 minutes, and cells were washed 3 times with cold PBS. Cells were then lysed with lysis buffer, and cross-linked chromatin was sonicated with Covaris M220. Sonicated chromatin was immunoprecipitated with anti-H3K27ac antibody (D5E4, Cell Signaling Technology), H3K4me2 (C64G9, Cell Signaling Technology), Pitx1 (sc-271435X, Santa Cruz Biotechnology), and Normal Rabbit IgG (2729, Cell Signaling Technology) at 4°C overnight. ChIP samples were washed, eluted with elution buffer for 65°C 30 minutes, and reverse cross-linked at 65°C overnight. DNA was purified using DNA Purification Buffers and Spin Columns (Cell Signaling Technology). Quantitative analysis of ChIP assays was performed by real-time PCR using specific primer pairs listed in Supplemental Table 2. For ChIP-Seq, sequencing libraries were prepared using TruSeq ChIP Sample Prep Kit (Illumina) according to the manufacturer’s protocol. Paired-end sequencing (100 bp) was performed on the HiSeq sequencer (Illumina). Two biological replicates were analyzed for H3K27ac ChIP-Seq.

Quality control and preprocessing of ChIP-Seq reads were performed using fastp (50). ChIP-Seq reads were mapped to the mm9 reference sequence using Bowtie2 (ver. 2.4.4) (51). Genomic ChIP-Seq peaks were detected using MACS2 (ver.2.2.4) (53) with the default setting (*q* value, 0.05), and input reads were used as control. ChIP-Seq tracks were visualized with IGV (ver.2.13.0) with input reads as control.

### Data analysis of ATAC-Seq and ChIP-Seq.

The ATAC-Seq and ChIP-Seq data sets of Sertoli cells (GSE99320) (29) were obtained from the Gene Expression Omnibus (GEO) database. Peak distribution and annotation analysis of ATAC-Seq peaks and ChIP-Seq peaks were performed using CEAS (Cis-regulatory Element Annotation System, ver. 1.0.2) (54) and GREAT (Genomic Regions Enrichment of Annotations Tool, ver. 4.0.4) (55). Chondrocyte-specific ATAC-Seq and ChIP-Seq peaks were extracted using BEDtools (ver. 2.31.0) (56), and enriched terms of Gene Ontology were obtained using GREAT. The enrichment ratio of ATAC-Seq and ChIP-Seq peaks in genomic TSSs and known enhancer regions were analyzed and visualized using ngs.plot (ver. 2.08) (57).

### Western blotting.

Cells were rinsed twice with PBS and solubilized in lysis buffer [20 mM HEPES at pH 7.4, 150 mM NaCl, 1 mM EGTA, 1.5 mM MgCl_2_, 10% glycerol, 1% Triton X-100, 10 μg/mL aprotinin, 10 μg/mL leupeptin, 1 mM 4-(2-aminoethyl) benzenesulfonyl fluoride hydrochloride, and 0.2 mM sodium orthovanadate]. The lysates were centrifuged at 4°C for 10 minutes at 15,000*g* and then boiled in sodium dodecyl sulfate (SDS) sample buffer containing 0.5 M β-mercaptoethanol for 5 minutes. The supernatant was separated by SDS-polyacrylamide gel electrophoresis, transferred to a nitrocellulose membrane, immunoblotted with a primary antibody, and then visualized with horseradish peroxidase–coupled anti-mouse or -rabbit IgG using an enhanced chemiluminescence detection kit (Immunostar LD; WAKO). Primary antibodies against Sox9 (AB5535, MilliporeSigma) and β-actin (M177-3 MBL) were used in this study.

### Reporter assay.

The *Sox9* minimal promoter region (–100 to +10) was amplified from the mouse genome by PCR and subcloned into pGL4.1[luc2] vector (Promega). The Sox9 enhancers and sequential deletions were introduced upstream of the Sox9 minimal promoter. Deletion of Pitx1 binding sites in the Sox9 enhancers was performed with inverse PCR using KOD-Plis-Mutagenesis kit (TOYOBO). Reporter genes were cotransfected with the expression vectors and Renilla into cells using the X-treme Gene9 DNA Transfection Reagent (MilliporeSigma). After 48 hours of transfection, the cells were lysed, and luciferase activity was measured using specific substrates in a luminometer (Promega) in accordance with the manufacturer’s protocol. Luciferase activity was normalized to Renilla.

### Biotinylated DNA pull-down assay.

HEK293 cells transfected with Flag-Pitx1 were lysed in RIPA lysis buffer and the lysates preincubated with Dynabeads M-280 streptavidin beads (Thermo Fisher Scientific). The lysates were then incubated with biotinylated double-stranded oligonucleotides containing the Pitx1 binding motifs in E160 (P1: 5′-ACGGTTGCTTTTTTCATCCGTGAGGTCAGAGC-3′, P2: 5′-GCAGCTGCCACCCTCAGCCCCCCACTTCGAGA-3′) and E308 (P3: 5′-GGCCATGCTGTCGGGAATATTTTCTCTCACCC-3′) with 5 μg of poly(dI-dC) for 3 hours at 4°C. DNA-bound proteins were collected with Dynabeads M-280 streptavidin beads, then washed 3 times with lysis buffer. Samples were boiled with 2× Laemmli buffer at 95°C for 5 minutes to elute DNA-bound proteins, separated on an SDS-polyacrylamide gel, and identified by Western blotting.

### Alcian blue staining.

Cells were washed with PBS and fixed with 3.7% formaldehyde in PBS for 10 minutes at room temperature. Then the cells were stained with 1% Alcian blue in 5% acetic acid for 10 minutes.

### RNA-Seq.

Total RNA was extracted as described above. Total RNA libraries were prepared using the TruSeq Stranded mRNA Library Prep kit (Illumina) in accordance with the manufacturer’s protocol. Sequencing was performed on an Illumina HiSeq 2500 platform in 75 bp single-end mode. Illumina Casava 1.8.2 software was used for base-calling. Sequenced reads were mapped to the mouse reference genome sequence (mm10) using TopHat v2.0.13 in combination with Bowtie2 (v2.2.3) and SAMtools (v0.1.19). Differentially expressed genes were identified using the following thresholds: FDR < 0.05 and minimum fold-change > 2.

### Generation of adenovirus.

Adenovirus cDNAs of Flag-tagged mouse Pitx1 were amplified using Pfu DNA polymerase and subcloned into pAXCAwt vectors (TAKARA). Recombinant adenoviruses were generated using the COS-TPC method by transfection of a recombinant cosmid and the DNA-TPC adenovirus genome into HEK293 cells (58). C3H10T1/2 cells, ATDC5 cells, primary chondrocytes, and primary limb bud cells were infected with adenoviruses at a multiplicity of infection of 20 unless indicated otherwise.

### Mice.

We generated single Sox9 enhancer deletion mice referred to as E160^Δ/Δ^ or E308^Δ/Δ^ according to the Technique for Animal Knockout system by Electroporation (TAKE) method (59) based on the CRISPR/Cas9 genome-editing system. Single-guide RNAs (sgRNAs), which flank the enhancer region, were designed using CRISPRdirect (https://crispr.dbcls.jp/) (60). Sequences of sgRNAs used for genome editing are listed in Supplemental Table 3. Mouse pronuclear-stage embryos were collected from C57BL/6J mice, and we introduced the gRNAs and 500 ng/mL Cas9 Nuclease (IDT) by electroporation using a super electroporator NEPA 21 (NEPA GENE Co. Ltd). All embryos were cultured overnight in KSOM mouse embryo medium, and the 2-cell–stage embryos were transferred to the oviducts of pseudopregnant females. We analyzed enhancer deletion by genomic PCR using specific primer pairs listed in Supplemental Table 4.

Because E160 and E308 are both located on Chr11, we performed 2-step genome editing using the TAKE method in order to generate the double enhancer deletion mice. Briefly, we first collected pronuclear-stage embryos of homozygous single E160 enhancer deletion mice (E160^Δ/Δ^ E308^WT/WT^) using an in vitro fertilization technique. Then sgRNAs for E308 and Cas9 Nuclease were injected into embryos, and these were transferred into oviducts of pseudopregnant females to generate E160^Δ/Δ^ E308^Δ/Δ^ mice. We crossed E160^Δ/Δ^ E308^Δ/Δ^ mice with wild-type mice to verify germline transmission and generate heterozygous E160/E308 deletion mice (E160^WT/Δ^ E308^WT/Δ^). For phenotypic analysis, we intercrossed heterozygous E160/E308 deletion mice to obtain wild-type and double-deletion mice (E160^Δ/Δ^ E308^Δ/Δ^). Mice were used regardless of the sex.

### Skeletal preparation.

The skin of the mice was removed, and the mice was fixed in 95% ethanol overnight. Cartilage tissues were stained with 1.5% Alcian blue followed by staining of bone tissues with 0.02% alizarin red S. Skeletal samples were photographed under a stereoscopic microscope (Stemi 2000-C, ZEISS).

### In situ hybridization.

The protocol for in situ hybridization has been described in a previous report (61). Briefly, tissues harvested from WT and enhancer deletion mice were fixed with 4% paraformaldehyde and then embedded in paraffin. The tissue blocks were cut into 4 μm–thick sections. Digoxigenin (DIG)-11-UTP–labeled, single-stranded RNA probes were prepared using a DIG RNA labeling kit (Roche), in accordance with the manufacturer’s instructions. We used a 0.4 kb fragment of the mouse *Col2a1* cDNA and a 0.5 kb fragment of the mouse *Sox9* cDNA to generate antisense and sense probes. Signals were detected with an alkaline phosphatase-conjugated anti-DIG antibody (11093274910, Roche). All probes were provided by Noriyuki Tsumaki (Department of Tissue Biochemistry, Graduate School of Medicine and Frontier Biosciences, Osaka University, Suita, Osaka, Japan).

### Immunohistochemistry.

Samples were fixed with 4% buffered paraformaldehyde, embedded in paraffin, and cut into 6 μm–thick sections. Paraffin-embedded sections were deparaffinized and rehydrated, followed by H&E staining. For immunohistochemical analysis, antigen retrieval was performed by incubation in DAKO REAL target retrieval solution for 10 minutes at 90°C, followed by blocking with 1% BSA in PBS. Immunohistochemistry was performed using the anti-Sox9 (AB5535, MilliporeSigma) antibody at 1:200 (vol/vol) dilution. Immunoreactivity was visualized with Alexa Fluor 555 dye–conjugated anti-rabbit IgG (A-21428, Invitrogen), and counterstaining was performed using 4′,6-diamidino-2-phenylindole, in accordance with the manufacturer’s protocol.

### Isolation of mouse limb bud cells.

The anterior and posterior limb buds of E12.5 wild-type and double enhancer deletion embryos were harvested and digested with DMEM containing 0.1% collagenase type II. The cells were dissociated by pipetting and then centrifuged at 300*g* for 5 minutes at 20°C. The pellet was resuspended and cultured in DMEM with 10% FBS at 37°C in a humidified 5% CO_2_ incubator.

### Statistics.

Randomization and blinding were not performed in the animal studies. Sample sizes were estimated based on previous studies of endochondral bone formation (61, 62). Data were statistically analyzed by unpaired 2-tailed Student’s *t* test for comparison between 2 groups. For more than 2 groups, we used 1-way ANOVA or 2-way ANOVA followed by Tukey’s multiple comparisons test. We performed 2 or 3 independent experiments for in vitro experiments including RT-qPCR and Western blotting unless otherwise stated. At least 5 mice were used for the phenotypic analysis. *P* values of less than 0.05 were considered statistically significant.

### Study approval.

All animal experiments were approved by the Osaka University Institute Animal Experiment Committee and performed in accordance with the Animal Research: Reporting of In Vivo Experiments guidelines.

### Data availability.

ATAC-Seq, ChIP-Seq, and RNA-Seq data that support the findings of this study have been deposited in the National Center for Biotechnology GEO with the accession code GSE237889 (https://www.ncbi.nlm.nih.gov/geo/query/acc.cgi?acc=GSE237889). All raw data values represented in graphs are available in the Supporting Data Values file.

## Author contributions

SIK and KH designed and performed all in vitro and in vivo experiments. SIK, KW, YT, TM, and KH performed molecular and biochemical experiments. HY, HT, and RY generated enhancer deletion mice. NU and RN discussed and assessed the data. KH and RN directed the project and interpreted the data. SIK, KH, KW, YT, TM, HY, HT, RY, NU, and RN participated in writing the paper.

## Supplementary Material

Supplemental data

Unedited blot and gel images

Supplemental tables 1-5

Supplemental table 6

Supporting data values

## Version 1 06/10/2024

Electronic publication
