# Supplementary material for: Chromatin profiling identifies chondrocyte-specific Sox9 enhancers important for skeletal development
Source: JCI Insight. 2024 Jun 10;9(11):e175486. doi: 10.1172/jci.insight.175486 (PMC11382882; doi:10.1172/jci.insight.175486)
Supplement: Supplemental data [file jciinsight-9-175486-s020.pdf]

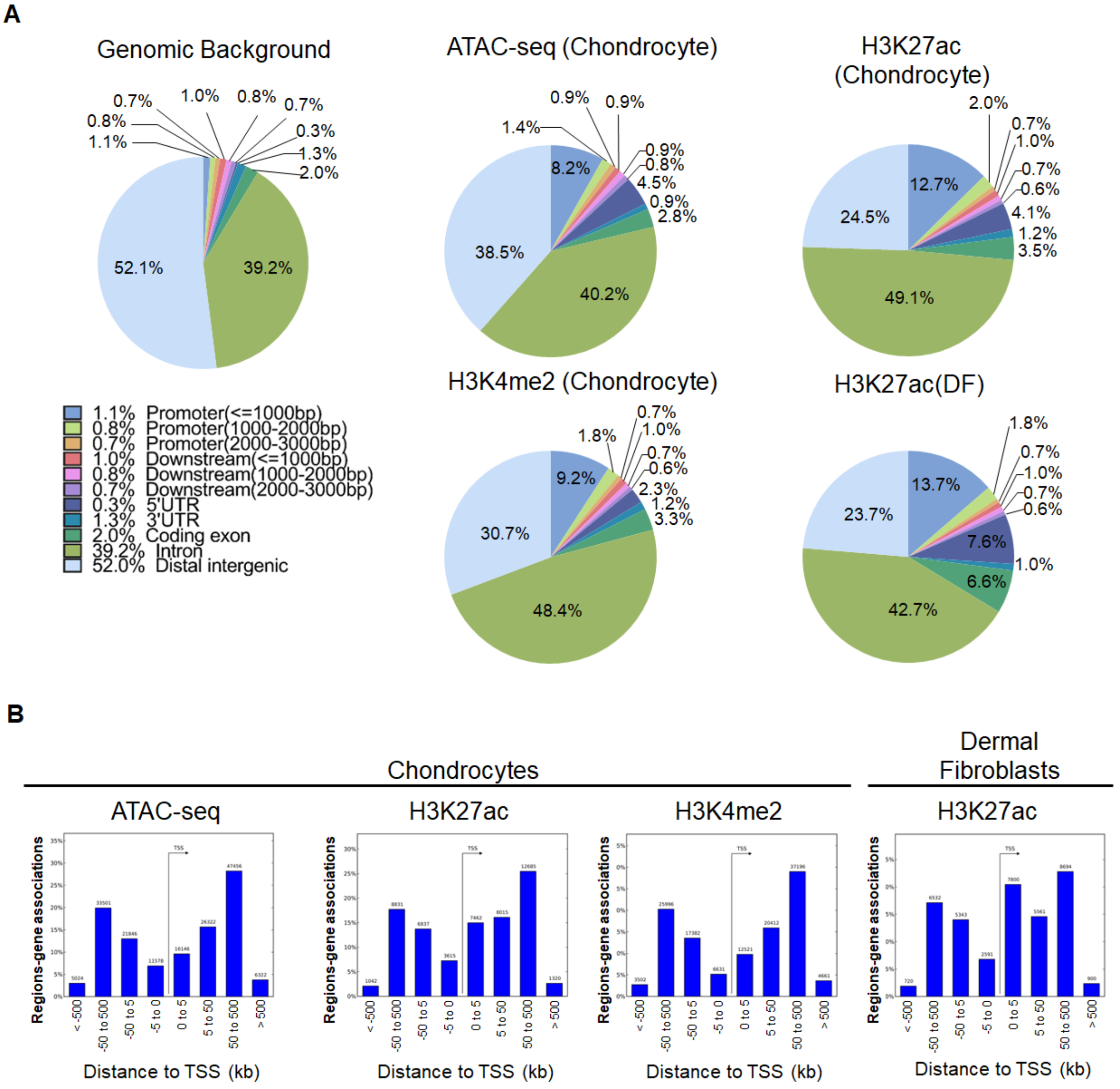

Supplementary Figure S1. Peak annotation of ATAC-seq and ChIP-Seq data sets obtained from primary chondrocytes and dermal fibroblasts.

(A) Distribution of ATAC-seq and ChIP-seq peaks over specific genomic features. **(B)** Distribution of ATAC-seq and ChIP-seq peaks at TSSs relative to the nearest gene, analyzed by GREAT.

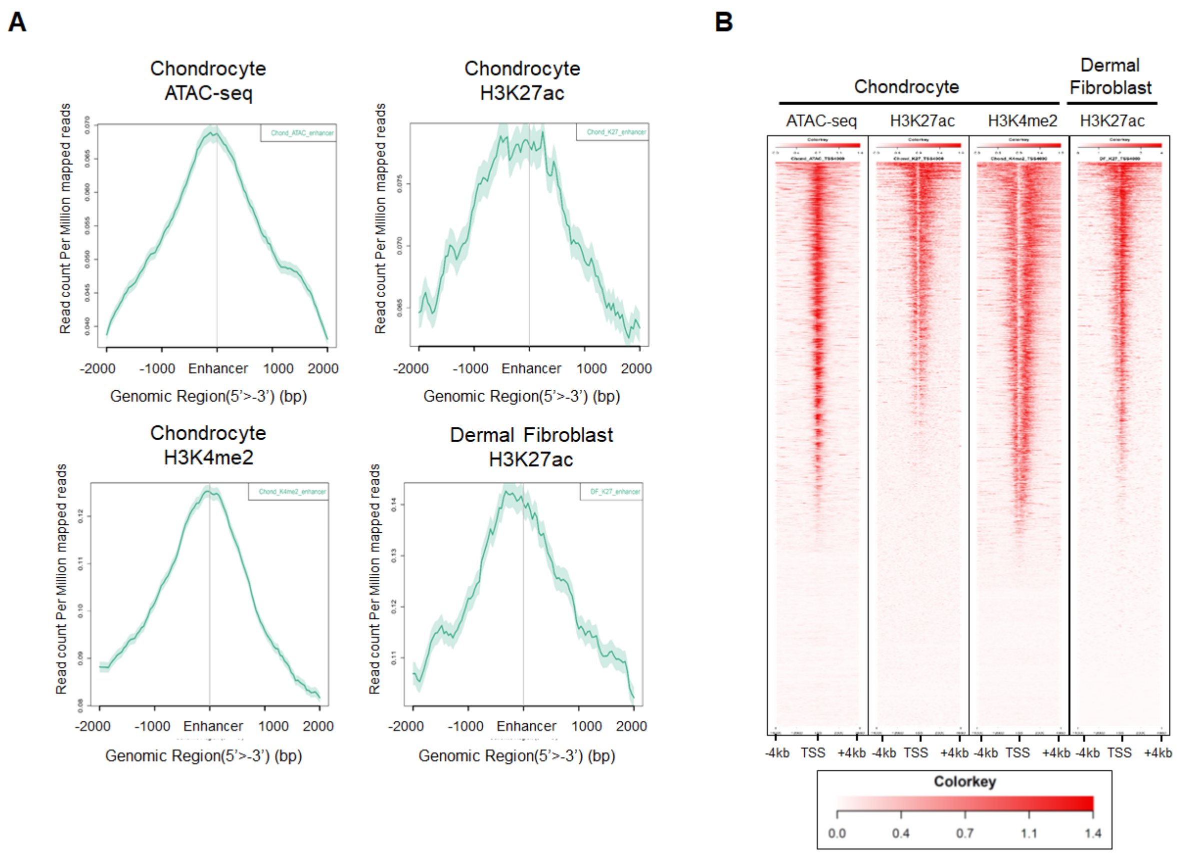

Supplementary Figure S2. Functional annotations of the ChIP-seq and ATAC-seq peaks in primary chondrocytes and dermal fibroblasts.

(A) Average profiles of ATAC-seq and ChIP-seq over enhancers based on ENCODE databases were calculated by ngs.plot. Y axis represents read count per million mapped reads.

(B) The log2 enrichment ratio of ATAC-seq and ChIP-seq data at TSS ± 4 Kb regions were calculated by ngs.plot and visualized as heatmaps.

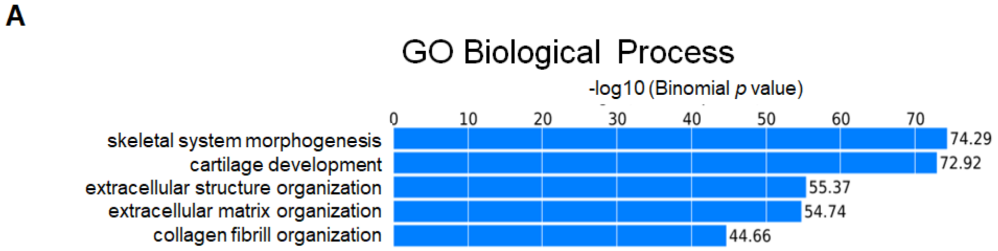

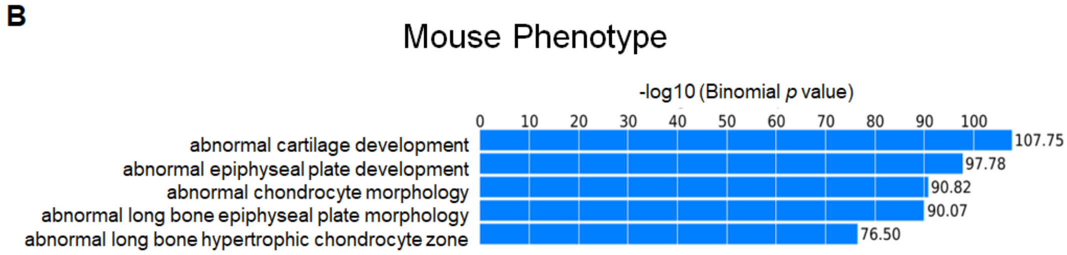

Supplementary Figure S3. Identification and characterization of chondrocyte specific enhancers

(A,B) GREAT GO analysis of chondrocyte specific peaks. The top five enriched terms for GO Biological Process (A) and Mouse Phenotype (B) are shown.

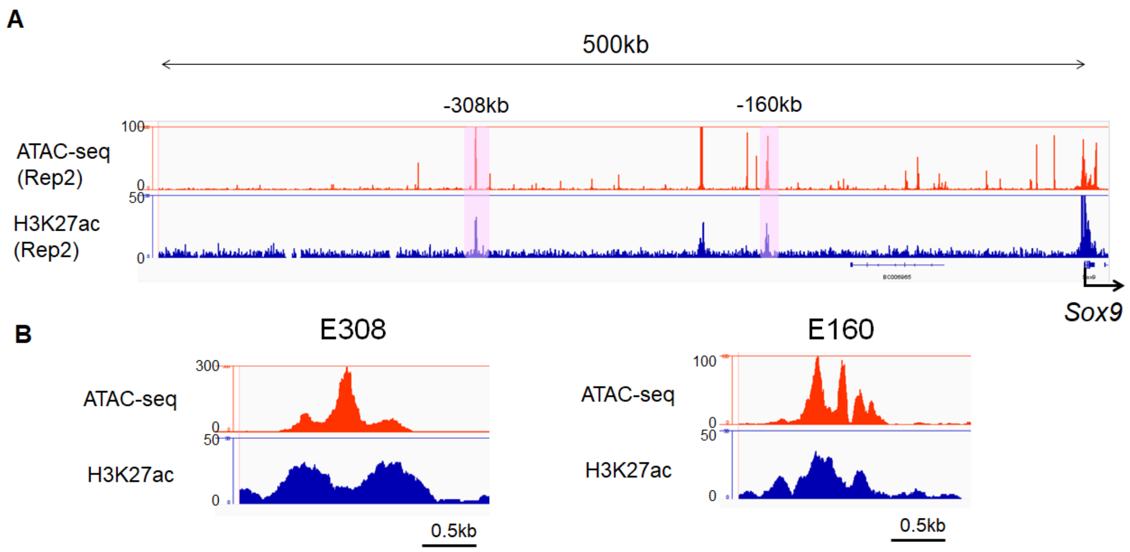

Supplementary Figure S4. Identification of E308 and E160 peaks from replicate ATAC-seq and H3K27 ChIP-seq.

(A) ATAC-seq and ChIP-seq profiles 500kb upstream of mouse Sox9 from replicate analysis. Genomic regions highlighted in pink show E308 and E160. (**B)** ATAC-seq and H3K27ac ChIP-seq profiles of E308 and E160.

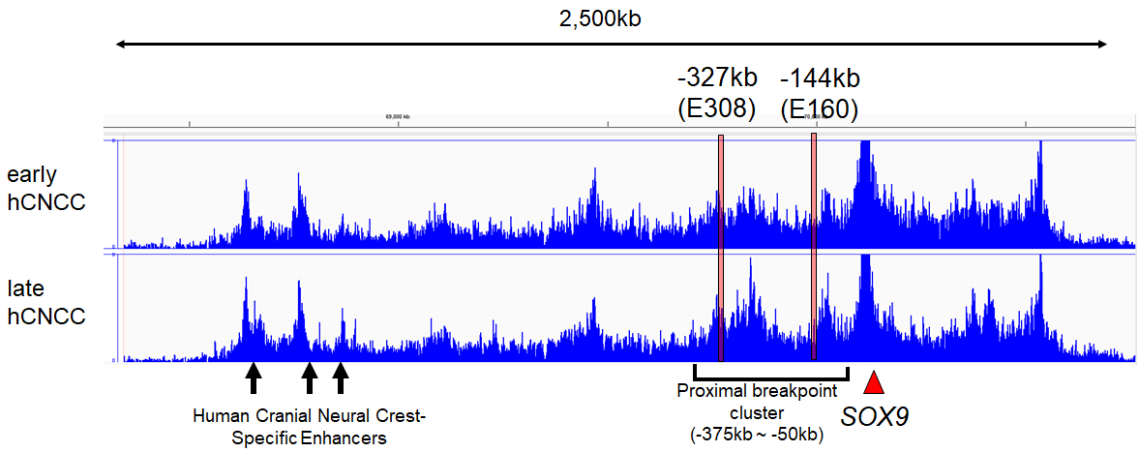

Supplementary Figure S5. SOX9 promoter-anchored Capture-C in early and late human cranial neural crest cells (GSE145327).

Light red shading highlights the homologous regions of E308 (-327kb) and E160 (-144kb) in the human genome. Red arrowhead indicate the *SOX9* promoter and black arrows indicate the human cranial neural crest-specific enhancers reported in ref(31). hCNCCs : human cranial neural crest cells

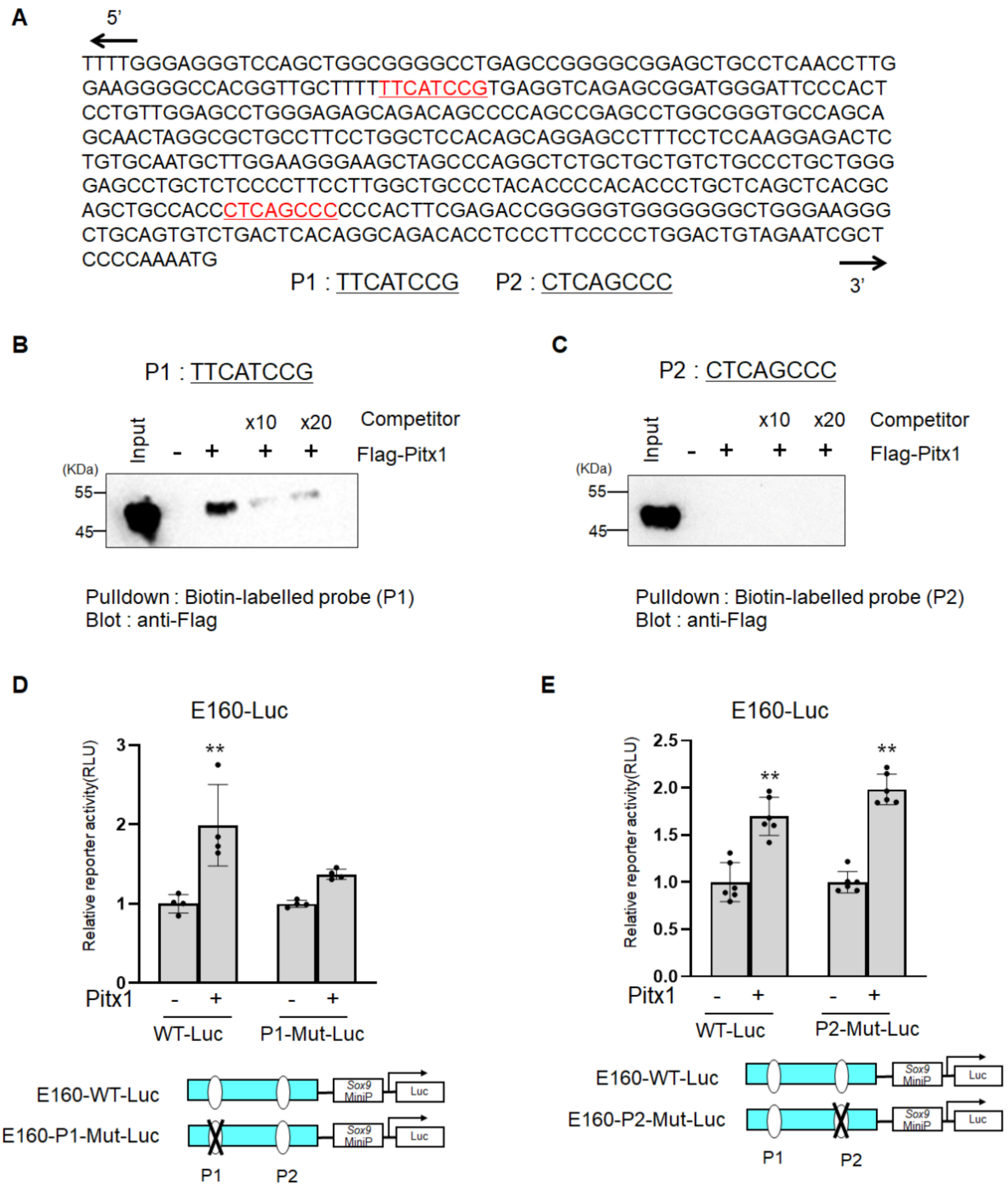

Supplementary Figure S6. Pitx1 promotes E160 enhancer activity.

(A) Candidate Pitx1 binding motifs in E160. Predicted motif sequence is colored in red and underlined. (**BC**) Biotinylated DNA pull-down assays using the Pitx1-binding motif in the E160 enhancer. Lysates of 293 cells transfected with Flag-Pitx1 were precipitated with biotinylated oligonucleotide containing P1 (B) and P2 (C) and DNA-bound Pitx was determined by immunoblotting with an anti-Flag antibody. (**DE**) Luciferase assay to evaluate the functionality of Pitx1 binding motifs P1 (D) and P2 (E) in E160. Luciferase reporter constructs of WT and mutant P1 or P2 were transfected into293 cells. Luciferase activities were measured 48 h after transfection. Data are shown as the mean ± s.d. (n = 4). **p < 0.01, unpaired Student’s t test

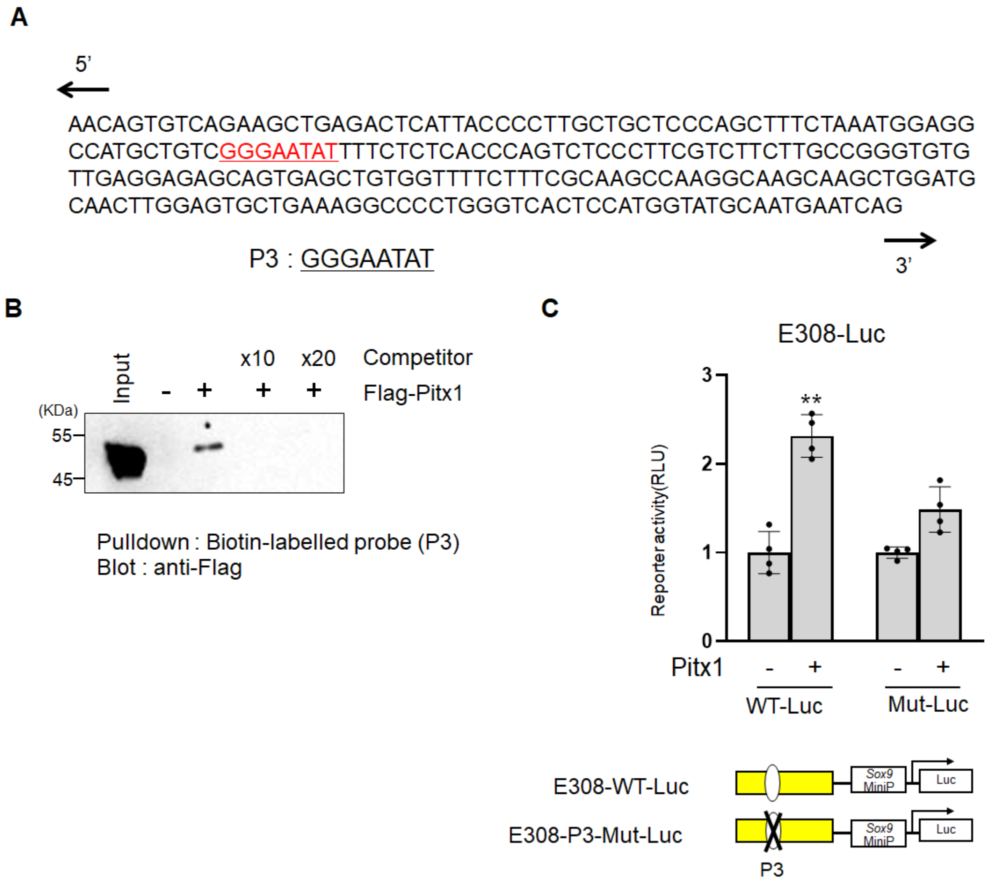

Supplementary Figure S7. Pitx1 promotes E308 enhancer activity.

(A) Candidate Pitx1 binding motifs in E308. The predicted motif sequence is colored in red and underlined. (**B**) Biotinylated DNA pull-down assays using the Pitx1-binding motif in the E308 enhancer. Lysates of 293 cells transfected with Flag-Pitx1 were precipitated with a biotinylated oligonucleotide based on P3 and DNA-bound Pitx was determined by immunoblotting with an anti-Flag antibody. (**C**) Luciferase assay to evaluate the functional Pitx1 binding motif in E308. Luciferase reporter constructs of WT and mutant P3 were transfected into 293 cells. Luciferase activities were measured 48 h after transfection. Data are shown as the mean ± s.d. (n = 4). **$p < 0.01$, unpaired Student's t test

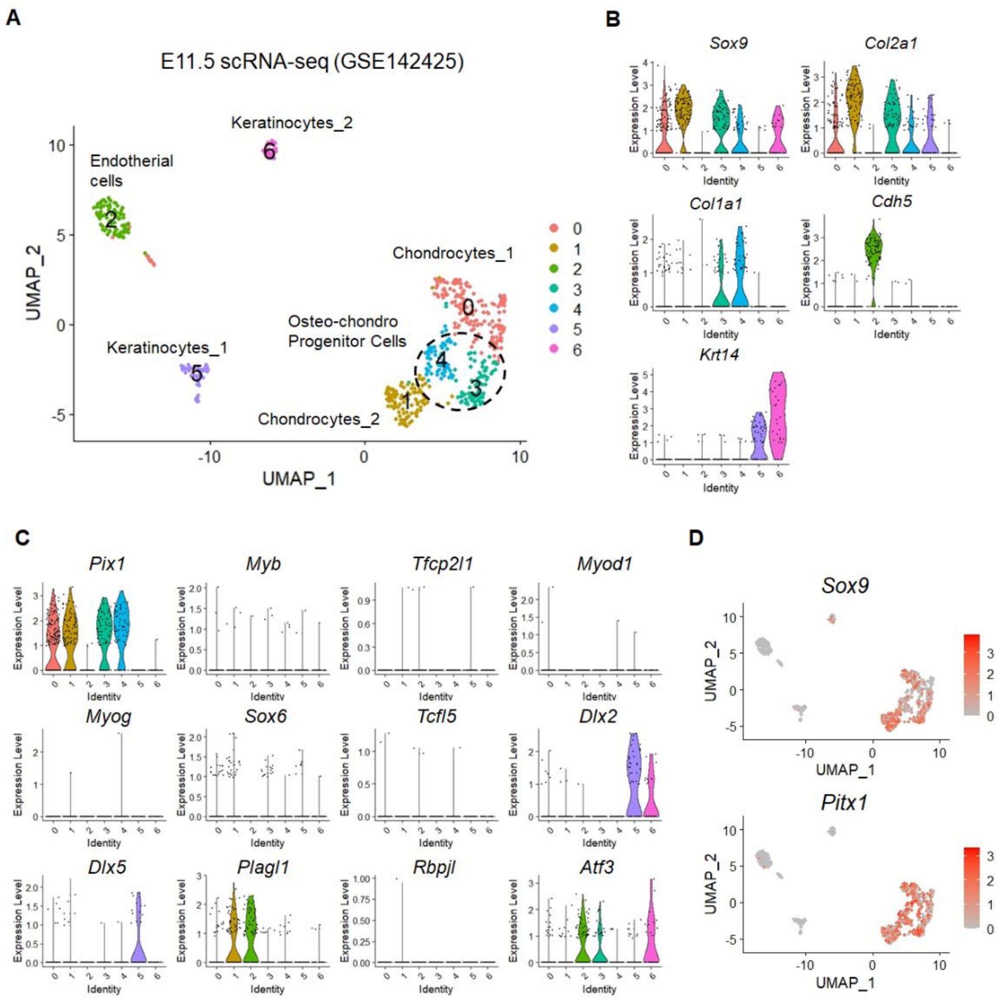

Supplementary Figure S8 The expression patterns of Sox9 and Pitx1 in E11.5 limb bud.
(A) UMAP plots of cells isolated from E11.5 limbs. The scRNA-seq dataset of E11.5 limb bud (GSE142425) was obtained from the GEO database and analyzed using Seurat. **(B)** Violin plots of marker gene expression for *Sox9, Col2a1,Col1a1, Cdh5, Krt14*. **(C)** Violin plots of candidate transcription factors identified in Fig.5a. **(D)** The expression patterns of Sox9 and Pitx1 in UMAP plots. Note that Pitx1 expression overlapped with Sox9.

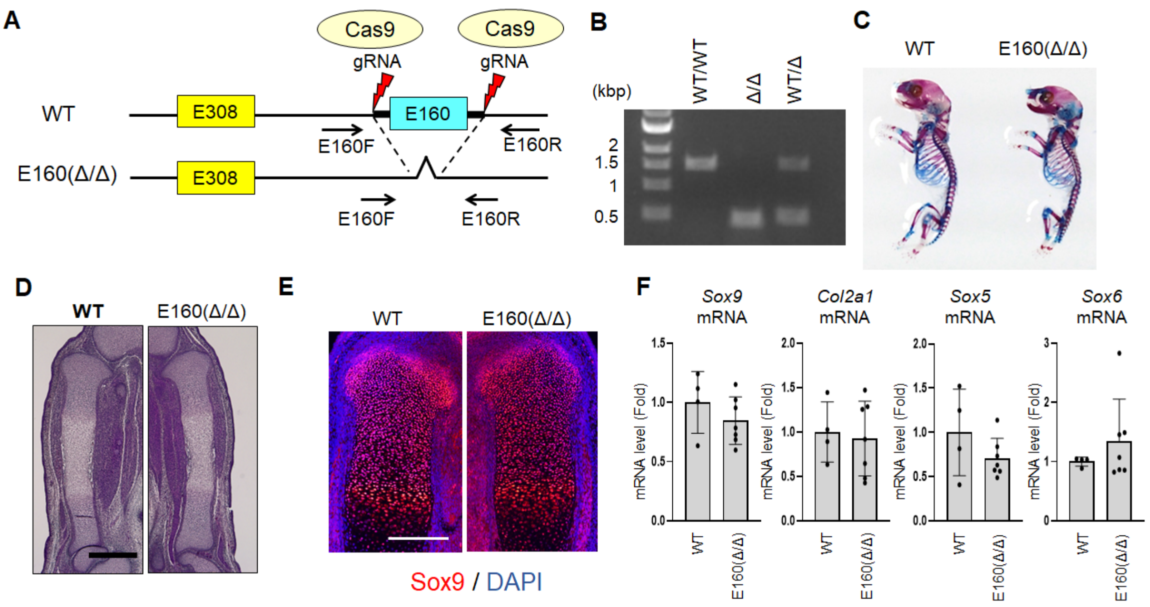

Supplementary Figure S9 Generation of single E160 deletion mice

(A) Schematic model of genome editing to delete E160. **(B)** Genotyping of E160(WT/WT), E160(Δ/Δ) and E160(WT/Δ) mice. PCR using the designed primer pairs to establish the E160 deletion generated a 1500 bp product from wild-type samples or a 480 bp products from E160(Δ/Δ) mice.

(C) Image of Alcian blue/Alizarin red S stained skeletal preparations of a newborn WT mouse and a E160(Δ/Δ) littermate.

(DE) Sections of tibiae of E15.0 WT and E160(Δ/Δ) littermate embryos stained with hematoxylin and eosin (H&E) (d) or by immunohistochemistry using an antibody against Sox9 (e). Scale bar: 500 μm

(F) Total RNA was isolated from forelimbs of E15.0 WT and E160(Δ/Δ) mouse embryos and analyzed by RT-qPCR. Data are shown as fold changes normalized to WT (mean ± s.d., WT : n = 4 animals. E160(Δ/Δ) : n=7 animals)

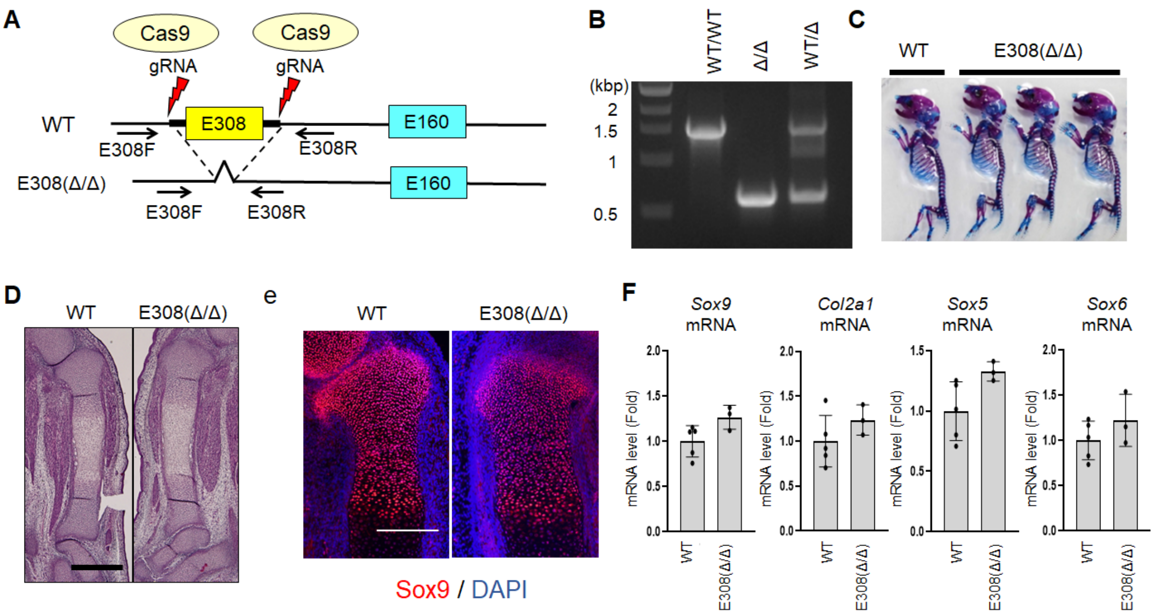

Supplementary Figure S10 Generation of single E308 deletion mice

(A) Schematic model of genome editing to delete E308. **(B)** Genotyping of E308(WT/WT), E308(Δ/Δ) and E308(WT/Δ) mice. PCR using primer pair for E308 deletion generated a 1400 bp product from wild-type samples or a 600 bp products from E308(Δ/Δ) mice.

(D) Image of Alcian blue/Alizarin red S stained skeletal preparations of newborn WT and E308(Δ/Δ) littermate mice.

(DE) Sections of tibiae E15.0 WT and E308(Δ/Δ) littermate embryos were examined by hematoxylin and eosin (H&E) staining and immunohistochemistry using antibody against Sox9. Scale bar: 500 μm

(F) Total RNA was isolated from forelimbs of E15.0 WT and E308(Δ/Δ) mice and analyzed by RT-qPCR. Data are shown as fold changes normalized to WT (mean ± s.d., WT : n = 5 animals. E308(Δ/Δ) : n=3 animals).

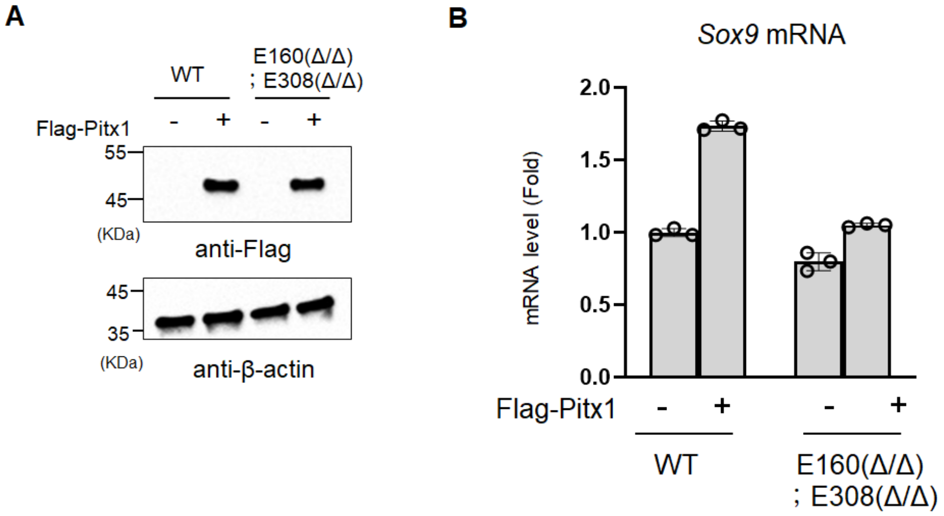

Supplementary Figure S11. Effect of Pitx1 on Sox9 expression in primary limb bud cells from WT and E160(Δ/Δ);E308(Δ/Δ) mice.

(**A**) Primary limb bud cells isolated from WT mice and E160(Δ/Δ);E308(Δ/Δ) littermates were infected with the control (−) or Flag-tagged Pitx1-expressing adenovirus and then cultured for 4 days. Cell lysates were analyzed using immunoblotting with anti-Flag and anti-β-actin antibodies. (**B**)Total RNA was isolated and Sox9 mRNA expression was determined by RT-qPCR. The RNA level is indicated as the fold increase compared to the WT control. The data are expressed as mean±s.d. of a technical triplicate. The data are representative of two independent experiments.
