# Supplementary material for: Chromatin profiling identifies chondrocyte-specific Sox9 enhancers important for skeletal development
Source: JCI Insight. 2024 Jun 10;9(11):e175486. doi: 10.1172/jci.insight.175486 (PMC11382882; doi:10.1172/jci.insight.175486)

Full unedited gel for Figure3B

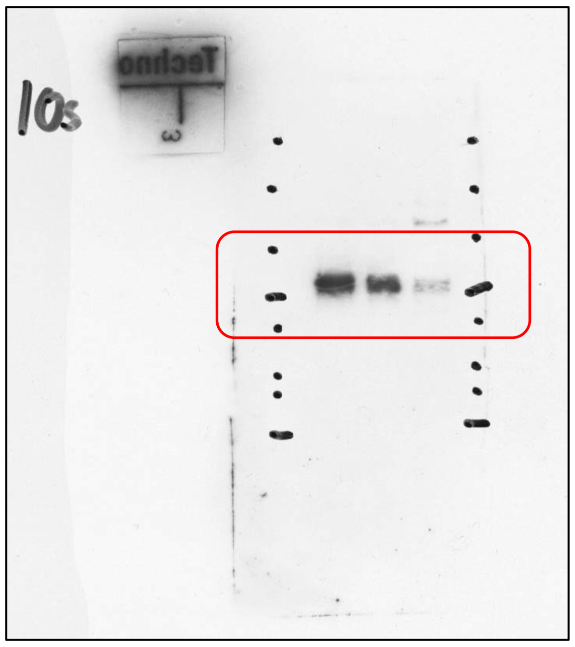

anti-Sox9

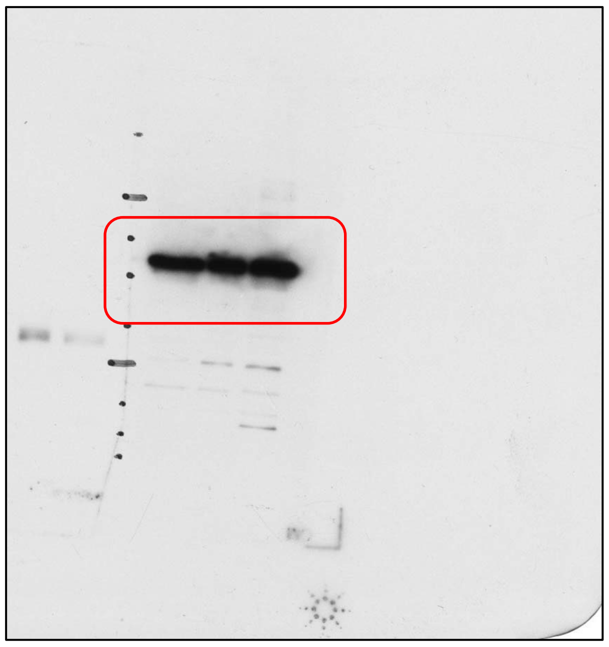

anti-bActin

Full unedited gel for Figure3D

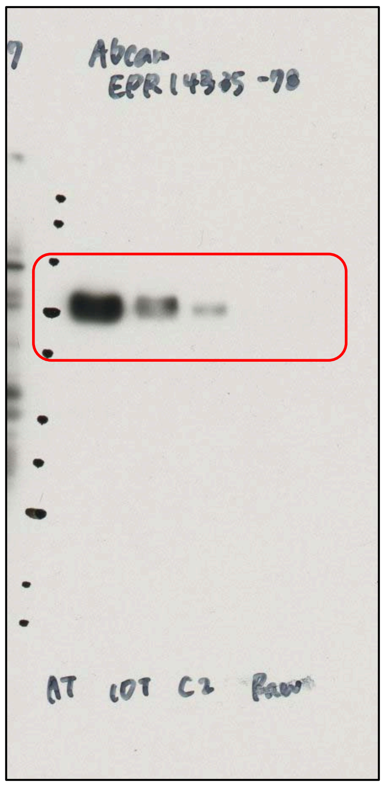

anti-Sox9

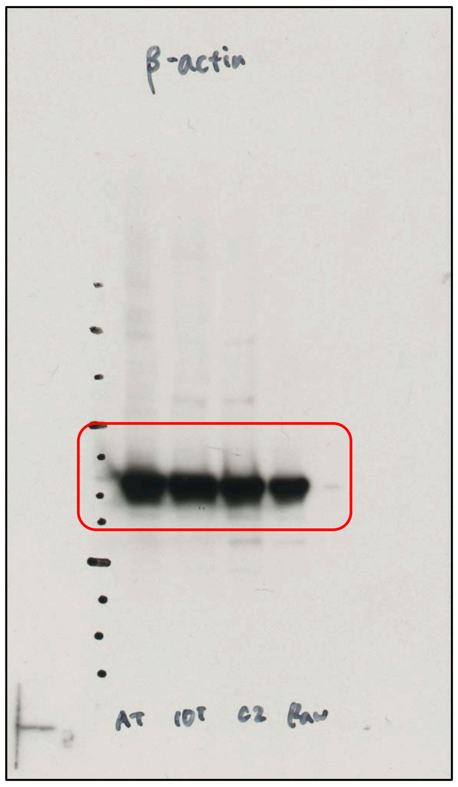

anti-bActin

Full unedited gel for Figure 6B upper panel

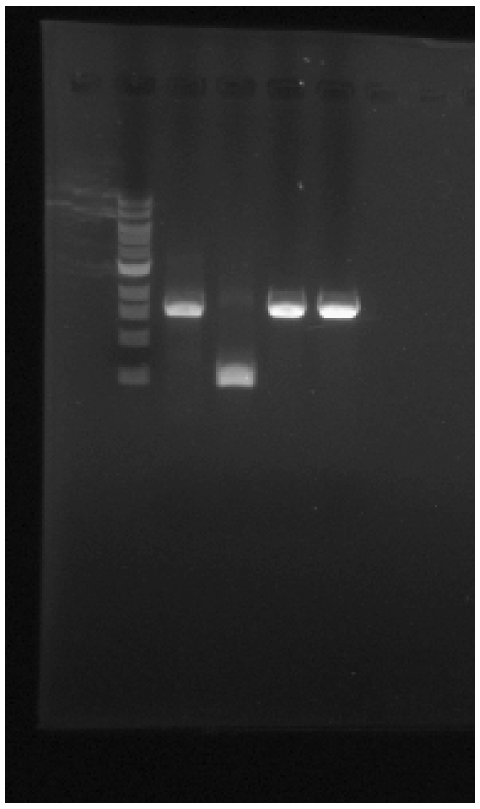

Full unedited gel for Figure 6B lower panel

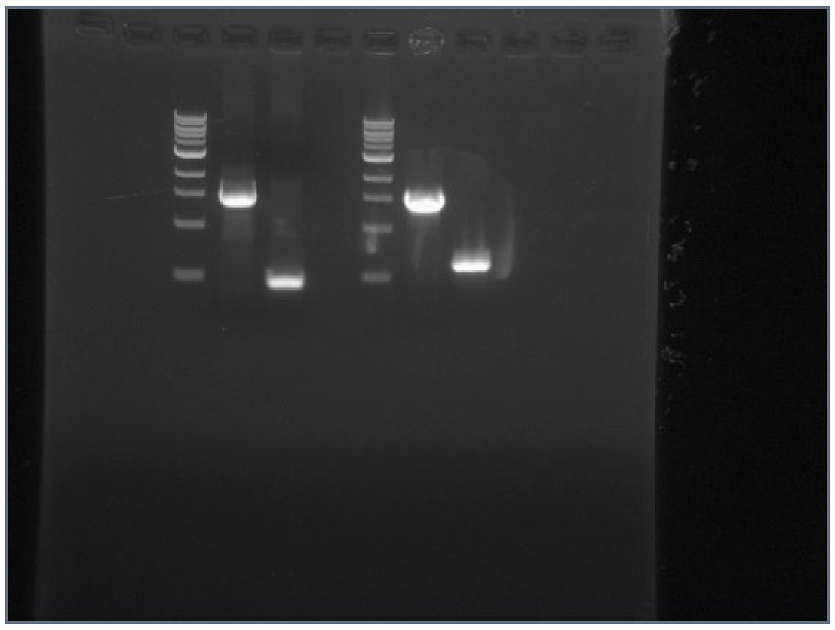

Full unedited gelfor Supplementary Figure 5B

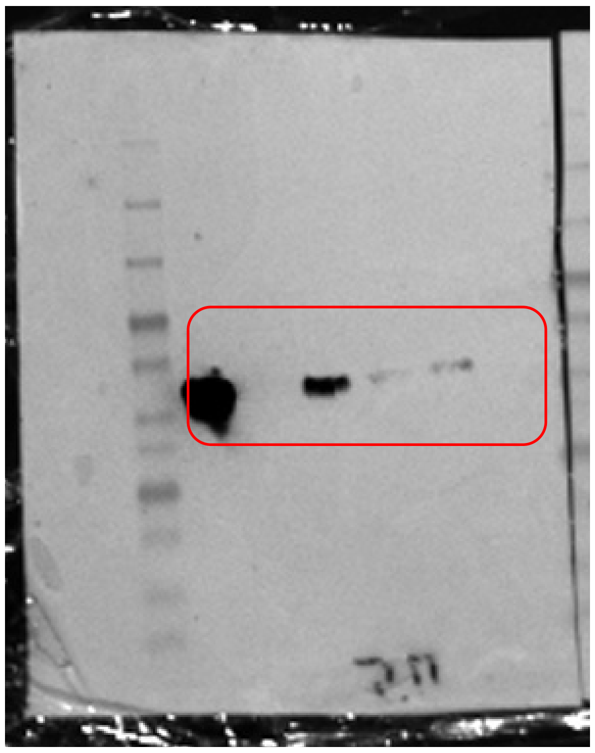

Full unedited gel for Supplementary Figure 5C

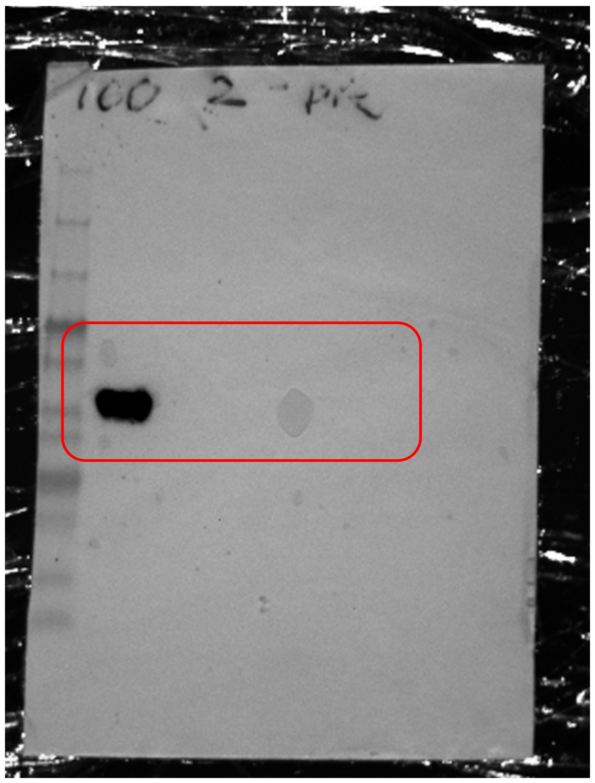

Full unedited gelfor
Supplementary Figure 6B

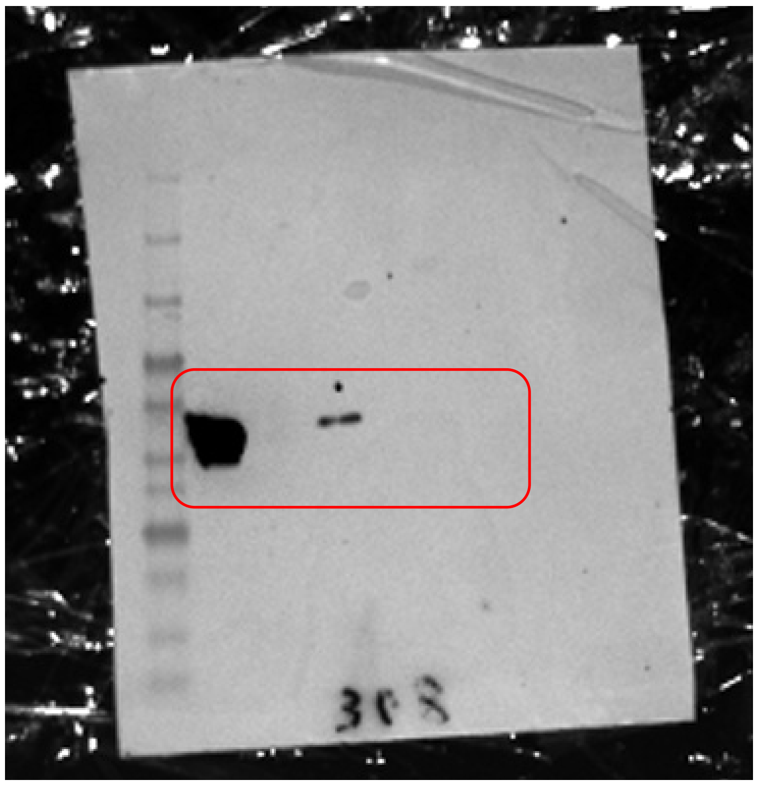

Full unedited gelfor
Supplementary Figure 8B

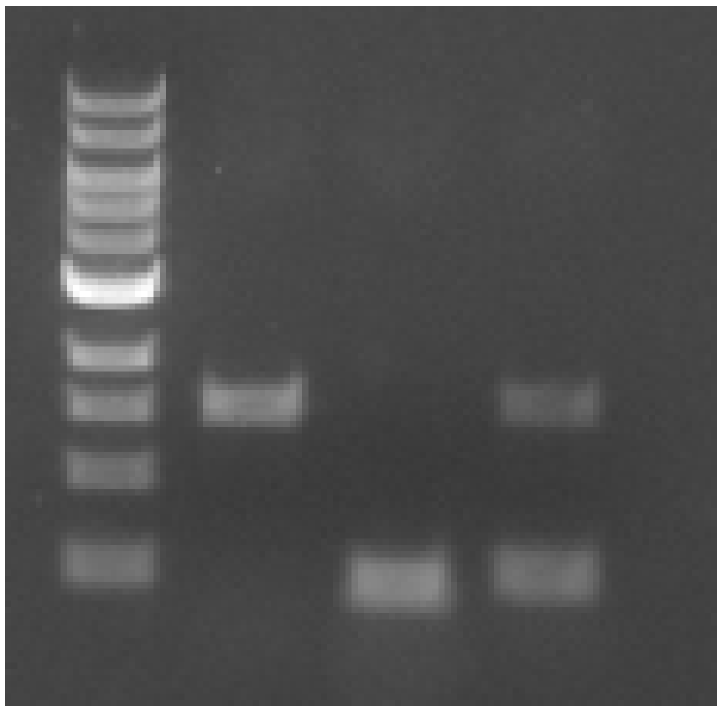

Full unedited gelfor
Supplementary Figure 9B

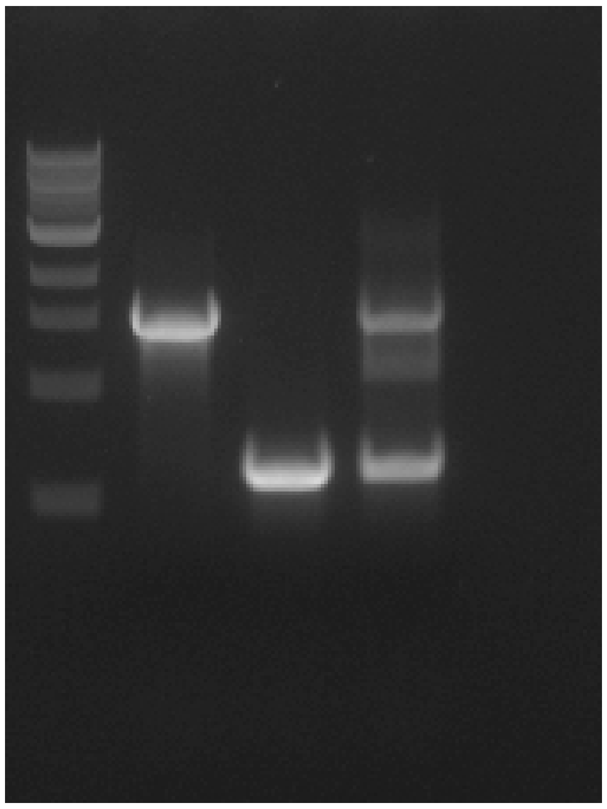

Full unedited gel for
Supplementary Figure 10A
anti-Flag

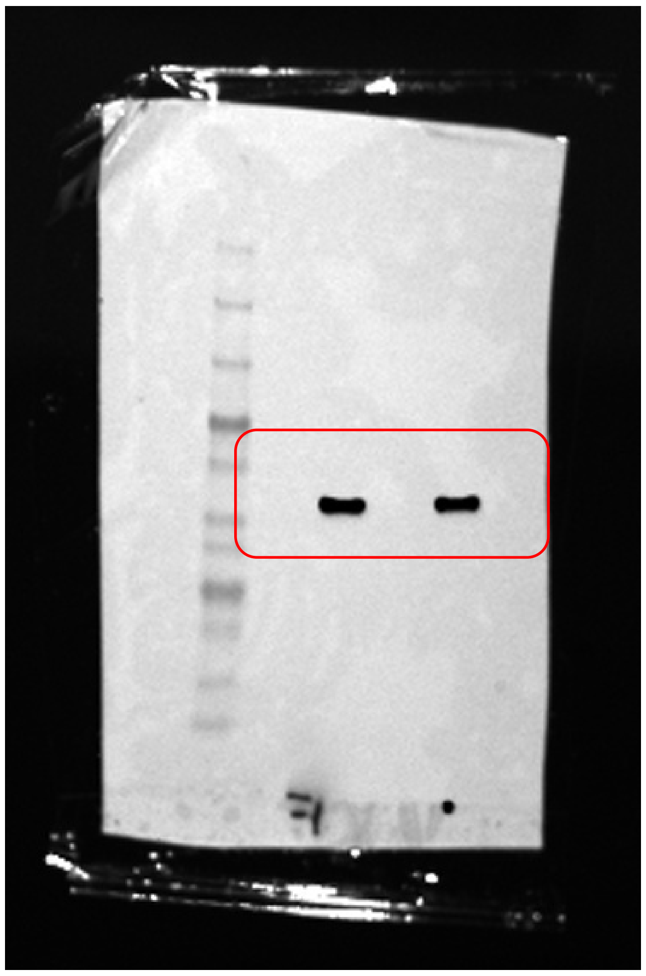

Full unedited gel for
Supplementary Figure 10A
anti-betaActin

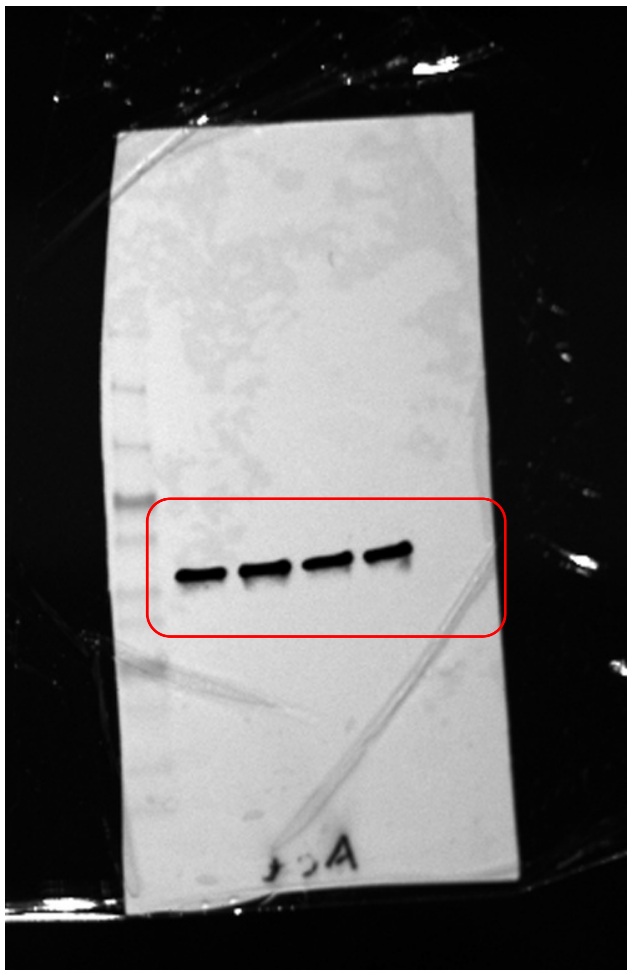

Supplement: Unedited blot and gel images [file jciinsight-9-175486-s021.pdf]
