# Supplementary material for: Chromatin profiling identifies chondrocyte-specific Sox9 enhancers important for skeletal development
Source: JCI Insight. 2024 Jun 10;9(11):e175486. doi: 10.1172/jci.insight.175486 (PMC11382882; doi:10.1172/jci.insight.175486)
Supplement: Supplemental tables 1-5 [file jciinsight-9-175486-s022.pdf]

Supplementary Table S1

Sequences of primer pairs used for RT-qPCR

Sox9	sense primer:	5'-CCTTCAACCTTCCTCACTACAGC-3'
	anti-sense primer:	5'-GGTGGAGTAGAGCCCTGAGC-3'
	probe:	5'-CCGCCCATCACCCGCTCGCAATAC-3'
Col1a1	sense primer:	5'-GCAACAGTCGCTTCACCTACA-3'
	anti-sense primer:	5'-CAATGTCCAAGGGAGCCACAT-3'
	probe:	5'-CCTTGTGGACGGCTGCACGAGTCAC
Col2a1	sense primer:	5'-CCTCCGTCTACTGTCCACTGAG-3'
	anti-sense primer:	5'-TGGAGCCCTGGATGAGCAAG-3'
	probe:	5'-TGAGGTTGCCAGCCGCTTCGTCCA-3'
Acan	sense primer:	5'-TCACTGTTACCGCCACTTTCC-3'
	anti-sense primer:	5'-TGCTGCTCAGATGTGACTGC-3'
	probe:	5'-ACCGTCTCTCCGCATCCACCCAGG-3'
Sox5	sense primer:	5‘-AGGCAGGAAATGCGACAGTAC-3'
	anti-sense primer:	5‘-CTCGGAGGGCAGGTGAGG-3'
	probe:	5‘-ACGTTGGGCAACAAGCACAGATCCCC-3'
Sox6	sense primer:	5‘-TACCCACAGCTCCCCTGAAG-3'
	anti-sense primer:	5'-CTCACCTTCAGTGGCAAGAGC-3'
	probe:	5‘-TCAGCAGCAGCGTTCACGAGCAGC-3'
Actb	sense primer:	5'-TTAATTTCTGAATGGCCCAGGTCT-3'
	anti-sense primer:	5'-ATTGGTCTCAAGTCAGTGTACAGG-3'
	probe:	5'-CCTGGCTGCCTCAACACCTCAACCC-3'

Supplementary Table S2

Sequences of primer pairs used for ChIP-qPCR

E160 Fwd	5’-AGAAGAGTCCTGGGCAGGTT-3’
E160 Rev	5’-AAACCCGGAGTTCAAGTGTG-3’
E308 Fwd	5’-CCCTTAGCTGCCAGTTTCTG-3’
E308 Rev	5’-TGGCGTCTCCACTTTTCTCT-3’

Supplementary TableS3

sgRNA sequences used for CRISPR/Cas9 based genome editing in mice. PAM sequences are underlined.

E160	gRNA #1	5’-TTGAAAAAATGACTGGTCTAAGG-3'
	gRNA #2	5’-GCTCACATCATACCACTGATTGG-3'
E308	gRNA #1	5’-TGCTAGGAGACTCGTCAATGGGG-3'
	gRNA #2	5’-CCTTCTCCAAGGTGGATTTCATG-3'
	gRNA #3	5’-CCTTTGGATTCCCTACCCACATG-3'
	gRNA #4	5’-CCACATGTGATAGATTAGACATA-3'

Supplementary TableS4

Sequences of primer pairs used for mice genotyping.

E160(Δ/Δ)	Fwd	5’-GTCCTAGGTGCCTGAAGCTG-3'
	Rev	5’-ACGTTGCAGTGTGTCCATGT-3'
E308(Δ/Δ)	Fwd	5’-CCCTTAGCTGCCAGTTTCTG-3'
	Rev	5’-GCAACATGGATCGACATCAC-3'

Supplementary TableS5

E308(1-220)

Matrix ID	Name	Score	Relative score	Start	End	Strand	Predicted sequence
MA0442.1	MA0442.1.SOX10	5.5609627	0.85132859	2	7	-	CACTGT
MA0442.1	MA0442.1.SOX10	6.8124285	0.906887448	3	8	+	CAGTGT
MA0067.1	MA0067.1.Pax2	5.4524574	0.858108057	3	10	-	TGACACTG
MA0099.2	MA0099.2.FOS::JUN	5.7234826	0.819663897	4	10	-	TGACACT
MA1684.1	MA1684.1.Foxn1	0.81124043	0.80424705	5	10	-	TGACAC
MA0499.1	MA0499.1.Myod1	0.9613175	0.802569548	7	19	-	CTCAGCTTCTGAC
MA0117.2	MA0117.2.Mafb	5.9185023	0.804522141	9	20	+	CAGAAGCTGAGA
MA0522.1	MA0522.1.Tcf3	3.876056	0.836805559	9	19	-	CTCAGCTTCTG
MA1684.1	MA1684.1.Foxn1	8.8971615	0.975416811	10	15	+	AGAAGC
MA2001.1	MA2001.1.Six4	7.335427	0.815302624	10	20	+	AGAAGCTGAGA
MA0500.1	MA0500.1.Myog	2.9717085	0.833838624	10	20	+	AGAAGCTGAGA
MA0633.2	MA0633.2.Twist2	1.8790631	0.820435717	10	19	-	CTCAGCTTCT
MA0633.2	MA0633.2.Twist2	1.8723779	0.820327366	10	19	+	AGAAGCTGAG
MA1997.1	MA1997.1.Olig2	1.4861706	0.815269346	10	19	+	AGAAGCTGAG
MA1997.1	MA1997.1.Olig2	1.4861706	0.815269346	10	19	-	CTCAGCTTCT
MA1993.1	MA1993.1.Neurod2	0.9751102	0.808406961	10	19	+	AGAAGCTGAG
MA1993.1	MA1993.1.Neurod2	0.9751102	0.808406961	10	19	-	CTCAGCTTCT
MA0164.1	MA0164.1.Nr2e3	6.2397103	0.806196413	12	18	-	TCAGCTT
MA0483.1	MA0483.1.Gfi1B	5.5141234	0.824741495	13	23	-	GAGTCTCAGCT
MA0489.2	MA0489.2.Jun	8.835745	0.867761639	16	27	+	TGAGACTCATTA
MA0912.1	MA0912.1.Hoxd3	8.203893	0.809081895	16	31	+	TGAGACTCATTACCCC
MA1988.1	MA1988.1.Atf3	7.7064753	0.854929877	16	26	+	TGAGACTCATT
MA0912.1	MA0912.1.Hoxd3	10.244001	0.849391362	18	33	-	AAGGGGTAATGAGTCT
MA0904.1	MA0904.1.Hoxb5	9.666488	0.806328561	18	33	+	AGACTCATTACCCCTT
MA0099.2	MA0099.2.FOS::JUN	6.57416	0.850715017	18	24	+	AGACTCA
MA1684.1	MA1684.1.Foxn1	1.0789376	0.809913896	18	23	+	AGACTC
MA0125.1	MA0125.1.Nobox	8.256175	0.897538814	20	27	-	TAATGAGT
MA0879.1	MA0879.1.Dlx1	7.6436896	0.897383995	20	29	+	ACTCATTACC
MA0877.1	MA0877.1.Barhl1	6.587822	0.879100253	20	29	-	GGTAATGAGT
MA1608.1	MA1608.1.Isl1	6.582556	0.834426601	20	30	+	ACTCATTACCC
MA0879.1	MA0879.1.Dlx1	5.108199	0.833220882	20	29	-	GGTAATGAGT
MA0881.1	MA0881.1.Dlx4	7.4450536	0.907981954	21	28	+	CTCATTAC
MA0880.1	MA0880.1.Dlx3	7.275687	0.901085958	21	28	+	CTCATTAC
MA0704.1	MA0704.1.Lhx4	7.247837	0.891052717	21	28	+	CTCATTAC
MA0075.2	MA0075.2.Prrx2	6.6198626	0.871078248	21	28	+	CTCATTAC
MA0720.1	MA0720.1.Shox2	6.161948	0.874511525	21	28	-	GTAATGAG
MA0705.1	MA0705.1.Lhx8	6.0736732	0.891957801	21	28	+	CTCATTAC
MA0720.1	MA0720.1.Shox2	5.8644986	0.867838812	21	28	+	CTCATTAC
MA0880.1	MA0880.1.Dlx3	5.3639965	0.842417599	21	28	-	GTAATGAG
MA0881.1	MA0881.1.Dlx4	5.240753	0.847843133	21	28	-	GTAATGAG
MA0075.2	MA0075.2.Prrx2	5.217405	0.826204841	21	28	-	GTAATGAG
MA0132.1	MA0132.1.Pdx1	5.029398	0.813150442	21	26	+	CTCATT
MA0704.1	MA0704.1.Lhx4	4.8203325	0.835220194	21	28	-	GTAATGAG
MA0709.1	MA0709.1.Msx3	4.785069	0.836701904	21	28	+	CTCATTAC
MA0705.1	MA0705.1.Lhx8	4.7570434	0.867087679	21	28	-	GTAATGAG
MA0709.1	MA0709.1.Msx3	4.46642	0.829576998	21	28	-	GTAATGAG
MA0125.1	MA0125.1.Nobox	5.3676677	0.802277202	22	29	+	TCATTACC
MA0132.1	MA0132.1.Pdx1	7.056204	0.896557716	23	28	-	GTAATG
MA0158.1	MA0158.1.HOXA5	4.698128	0.818340945	23	30	-	GGGTAATG
MA0697.2	MA0697.2.Zic3	8.198041	0.811016724	27	39	-	AGCAGCAAGGGGT
MA0098.2	MA0098.2.Ets1	6.938309	0.814096408	27	41	+	ACCCCTTGCTGCTCC
MA1628.1	MA1628.1.Zic1::Zic2	5.9193983	0.836566359	29	39	-	AGCAGCAAGGG
MA0145.2	MA0145.2.Tfcp2l1	5.6221633	0.813014646	31	44	+	CTTGCTGCTCCCAG
MA0039.2	MA0039.2.Klf4	4.319318	0.809893748	34	43	-	TGGGAGCAGC
MA1684.1	MA1684.1.Foxn1	0.7151981	0.802213943	34	39	-	AGCAGC
MA1684.1	MA1684.1.Foxn1	1.5127487	0.819097184	37	42	-	GGGAGC
MA0739.1	MA0739.1.Hic1	4.2684994	0.802838532	38	46	+	CTCCCAGCT
MA1472.2	MA1472.2.Bhlha15	6.2577596	0.830209447	39	50	+	TCCCAGCTTTCT
MA1472.2	MA1472.2.Bhlha15	6.257743	0.830209148	39	50	-	AGAAAGCTGGGA
MA1619.1	MA1619.1.Ptf1A	5.5668244	0.812418668	39	50	+	TCCCAGCTTTCT
MA1619.1	MA1619.1.Ptf1A	5.551114	0.812112048	39	50	-	AGAAAGCTGGGA
MA0521.2	MA0521.2.Tcf12	4.9394145	0.809755375	39	50	+	TCCCAGCTTTCT
MA0521.2	MA0521.2.Tcf12	4.9163117	0.809274746	39	50	-	AGAAAGCTGGGA
MA0500.1	MA0500.1.Myog	4.790487	0.858205689	39	49	-	GAAAGCTGGGA
MA1993.1	MA1993.1.Neurod2	3.2892256	0.844044487	40	49	+	CCCAGCTTTC

MA1993.1	MA1993.1.Neurod2	3.2892256	0.844044487	40	49	-	GAAAGCTGGG
MA0633.2	MA0633.2.Twist2	3.0698373	0.839735256	40	49	+	CCCAGCTTTC
MA0633.2	MA0633.2.Twist2	3.063152	0.839626905	40	49	-	GAAAGCTGGG
MA1997.1	MA1997.1.Olig2	2.6642222	0.833788408	40	49	+	CCCAGCTTTC
MA1997.1	MA1997.1.Olig2	2.6642222	0.833788408	40	49	-	GAAAGCTGGG
MA0522.1	MA0522.1.Tcf3	2.1883566	0.814356141	40	50	+	CCCAGCTTTCT
MA0499.1	MA0499.1.Myod1	1.7932584	0.813264447	40	52	+	CCCAGCTTTCTAA
MA0164.1	MA0164.1.Nr2e3	7.3300366	0.845144404	41	47	+	CCAGCTT
MA0100.2	MA0100.2.Myb	3.1831584	0.80020886	41	50	+	CCAGCTTTCT
MA1684.1	MA1684.1.Foxn1	1.5127487	0.819097184	44	49	-	GAAAGC
MA0442.1	MA0442.1.SOX10	6.6364155	0.899073346	45	50	+	CTTTCT
MA0102.2	MA0102.2.CEBPA	6.9769645	0.847080965	46	54	+	TTTCTAAAT
MA0095.3	MA0095.3.Yy1	9.024005	0.852592574	47	58	+	TTCTAAATGGAG
MA0877.1	MA0877.1.Barhl1	8.157853	0.920474274	48	57	+	TCTAAATGGA
MA1608.1	MA1608.1.Isl1	5.408308	0.807302208	48	58	-	CTCCATTTAGA
MA0879.1	MA0879.1.Dlx1	4.691869	0.822685228	48	57	-	TCCATTTAGA
MA0881.1	MA0881.1.Dlx4	5.611723	0.857964115	49	56	-	CCATTTAG
MA0880.1	MA0880.1.Dlx3	5.3414764	0.841726475	49	56	-	CCATTTAG
MA0704.1	MA0704.1.Lhx4	5.122345	0.84216647	49	56	-	CCATTTAG
MA0132.1	MA0132.1.Pdx1	5.0746384	0.815012179	49	54	+	CTAAAT
MA0075.2	MA0075.2.Prrx2	4.9172354	0.816600539	49	56	-	CCATTTAG
MA0720.1	MA0720.1.Shox2	4.36351	0.834167006	49	56	-	CCATTTAG
MA0709.1	MA0709.1.Msx3	3.8175366	0.815068126	49	56	-	CCATTTAG
MA0877.1	MA0877.1.Barhl1	3.7466967	0.804229908	49	58	+	CTAAATGGAG
MA0705.1	MA0705.1.Lhx8	2.4165802	0.822878137	49	56	-	CCATTTAG
MA0705.1	MA0705.1.Lhx8	2.3678064	0.821956838	49	56	+	CTAAATGG
MA0704.1	MA0704.1.Lhx4	3.3310347	0.800966392	50	57	-	TCCATTTA
MA0027.1	MA0027.1.En1	6.9634633	0.903088703	51	61	+	AAATGGAGGCC
MA1996.1	MA1996.1.Nr1H2	4.532388	0.802572166	54	64	+	TGGAGGCCATG
MA0643.1	MA0643.1.Esrrg	3.3251343	0.805090878	54	63	+	TGGAGGCCAT
MA0018.2	MA0018.2.CREB1	6.140726	0.801224309	55	62	+	GGAGGCCA
MA1110.2	MA1110.2.Nr1H4	3.9903626	0.820919073	55	64	+	GGAGGCCATG
MA1684.1	MA1684.1.Foxn1	2.7010925	0.844253071	55	60	+	GGAGGC
MA0146.2	MA0146.2.Zfx	6.326198	0.80299209	56	69	-	GACAGCATGGCCTC
MA0095.3	MA0095.3.Yy1	6.5808053	0.804765101	58	69	-	GACAGCATGGCC
MA0067.1	MA0067.1.Pax2	4.648885	0.826552762	58	65	+	GGCCATGC
MA1628.1	MA1628.1.Zic1::Zic2	7.1311007	0.857846482	59	69	-	GACAGCATGGC
MA0623.1	MA0623.1.Neurog1	5.6583557	0.834098256	59	68	-	ACAGCATGGC
MA0469.1	MA0469.1.E2F3	5.2287107	0.803808579	60	74	-	TTCCCGACAGCATGG
MA0092.1	MA0092.1.Hand1::Tcf<br>3	7.2698116	0.837772027	66	75	+	TGTCGGGAAT
MA1153.1	MA1153.1.Smad4	5.662469	0.836136018	66	73	+	TGTCGGGA
MA0682.1	MA0682.1.Pitx1	4.4940104	0.806541555	70	77	-	ATATTCCC
MA0623.1	MA0623.1.Neurog1	5.987901	0.841750289	71	80	+	GGAATATTTT
MA0623.1	MA0623.1.Neurog1	4.9286356	0.817154185	71	80	-	AAAATATTCC
MA0461.2	MA0461.2.Atoh1	4.7808113	0.812029694	71	80	-	AAAATATTCC
MA0879.1	MA0879.1.Dlx1	5.297433	0.838009631	73	82	-	AGAAAATATT
MA0879.1	MA0879.1.Dlx1	3.8523407	0.801440133	73	82	+	AATATTTTCT
MA0482.1	MA0482.1.Gata4	6.520386	0.865535358	77	87	+	TTTTCTCTCAC
MA1567.2	MA1567.2.Tbx6	8.109382	0.831356362	79	90	-	TGGGTGAGAGAA
MA0472.1	MA0472.1.Egr2	5.522738	0.802099912	81	95	+	CTCTCACCCAGTCTC
MA0006.1	MA0006.1.Ahr::Arnt	5.2968884	0.81730469	85	90	-	TGGGTG
MA0079.2	MA0079.2.SP1	8.857075	0.854354495	87	96	+	CCCAGTCTCC
MA0442.1	MA0442.1.SOX10	4.5390506	0.805960776	89	94	+	CAGTCT
MA0515.1	MA0515.1.Sox6	7.972387	0.866468494	96	105	+	CCTTCGTCTT
MA0143.3	MA0143.3.Sox2	5.848521	0.85600148	96	103	+	CCTTCGTC
MA0442.1	MA0442.1.SOX10	4.6051607	0.808895737	97	102	+	CTTCGT
MA0604.1	MA0604.1.Atf1	5.2307816	0.831431211	98	105	-	AAGACGAA
MA1684.1	MA1684.1.Foxn1	1.0789376	0.809913896	99	104	-	AGACGA
MA1684.1	MA1684.1.Foxn1	0.7151981	0.802213943	102	107	-	AGAAGA
MA1621.1	MA1621.1.Rbpjl	6.628391	0.811127526	107	120	-	AACACACCCGGCAA
MA1620.1	MA1620.1.Ptf1A	6.1238537	0.805539775	108	119	-	ACACACCCGGCA
MA1628.1	MA1628.1.Zic1::Zic2	4.4341655	0.810482447	108	118	-	CACACCCGGCA
MA0522.1	MA0522.1.Tcf3	1.4384923	0.804381604	108	118	-	CACACCCGGCA
MA0493.1	MA0493.1.Klf1	6.7618217	0.825922509	111	121	-	CAACACACCCG
MA0039.2	MA0039.2.Klf4	5.9852004	0.838196784	111	120	+	CGGGTGTGTT
MA0006.1	MA0006.1.Ahr::Arnt	4.890665	0.800069129	111	116	+	CGGGTG
MA0068.1	MA0068.1.Pax4	12.283871	0.806475509	121	150	-	GAAAGAAAACCACAGCTCAC<br>TGCTCTCCTC
MA0067.1	MA0067.1.Pax2	4.1654134	0.807567443	129	136	-	GCTCACTG
MA1110.2	MA1110.2.Nr1H4	4.3709736	0.82869036	130	139	-	ACAGCTCACT
MA0500.1	MA0500.1.Myog	1.2755638	0.811114541	130	140	-	CACAGCTCACT
MA0633.2	MA0633.2.Twist2	2.6132214	0.832334628	131	140	-	CACAGCTCAC

MA0633.2	MA0633.2.Twist2	2.6065361	0.832226277	131	140	+	GTGAGCTGTG
MA1997.1	MA1997.1.Olig2	2.373253	0.829214351	131	140	+	GTGAGCTGTG
MA1997.1	MA1997.1.Olig2	2.373253	0.829214351	131	140	-	CACAGCTCAC
MA1993.1	MA1993.1.Neurod2	1.758776	0.820475464	131	140	+	GTGAGCTGTG
MA1993.1	MA1993.1.Neurod2	1.758776	0.820475464	131	140	-	CACAGCTCAC
MA0499.1	MA0499.1.Myod1	1.5449129	0.810071877	131	143	+	GTGAGCTGTGGTT
MA1989.1	MA1989.1.Bcl11B	13.272015	0.911408942	133	146	-	GAAAACCACAGCTC
MA0483.1	MA0483.1.Gfi1B	14.497661	0.959594611	134	144	-	AAACCACAGCT
MA0002.2	MA0002.2.Runx1	13.45155	0.960362937	134	144	+	AGCTGTGGTTT
MA0027.1	MA0027.1.En1	5.5912433	0.84805517	136	146	+	CTGTGGTTTTC
MA0482.1	MA0482.1.Gata4	2.474555	0.807367185	140	150	+	GGTTTTCTTTC
MA0102.2	MA0102.2.CEBPA	6.7858696	0.840197317	146	154	-	TTGCGAAAG
MA0102.2	MA0102.2.CEBPA	9.368581	0.933232126	147	155	+	TTTCGCAAG
MA0632.1	MA0632.1.Tcfl5	4.8607755	0.838701415	148	157	+	TTCGCAAGCC
MA0632.1	MA0632.1.Tcfl5	4.8607755	0.838701415	148	157	-	GGCTTGCGAA
MA1684.1	MA1684.1.Foxn1	1.5127487	0.819097184	151	156	+	GCAAGC
MA0145.2	MA0145.2.Tfcp2l1	6.7642236	0.829635585	152	165	+	CAAGCCAAGGCAAG
MA1684.1	MA1684.1.Foxn1	1.5127487	0.819097184	161	166	+	GCAAGC
MA0006.1	MA0006.1.Ahr::Arnt	6.158047	0.853842612	162	167	-	TGCTTG
MA0095.3	MA0095.3.Yy1	7.2218943	0.8173149	164	175	+	AGCAAGCTGGAT
MA0522.1	MA0522.1.Tcf3	3.38505	0.830274303	165	175	+	GCAAGCTGGAT
MA1993.1	MA1993.1.Neurod2	2.6837294	0.834719807	165	174	+	GCAAGCTGGA
MA1993.1	MA1993.1.Neurod2	2.6837294	0.834719807	165	174	-	TCCAGCTTGC
MA0633.2	MA0633.2.Twist2	2.6456656	0.83286047	165	174	+	GCAAGCTGGA
MA0633.2	MA0633.2.Twist2	2.636164	0.832706471	165	174	-	TCCAGCTTGC
MA1997.1	MA1997.1.Olig2	2.291951	0.827936275	165	174	+	GCAAGCTGGA
MA1997.1	MA1997.1.Olig2	2.291951	0.827936275	165	174	-	TCCAGCTTGC
MA1684.1	MA1684.1.Foxn1	1.5127487	0.819097184	165	170	+	GCAAGC
MA0164.1	MA0164.1.Nr2e3	7.3300366	0.845144404	166	172	+	CAAGCTG
MA0164.1	MA0164.1.Nr2e3	7.3300366	0.845144404	167	173	-	CCAGCTT
MA0145.2	MA0145.2.Tfcp2l1	12.293883	0.91011132	170	183	-	CAAGTTGCATCCAG
MA0145.2	MA0145.2.Tfcp2l1	6.6008334	0.827257691	170	183	+	CTGGATGCAACTTG
MA0027.1	MA0027.1.En1	4.961689	0.822806599	172	182	-	AAGTTGCATCC
MA1684.1	MA1684.1.Foxn1	2.7010925	0.844253071	172	177	+	GGATGC
MA0113.2	MA0113.2.NR3C1	6.036217	0.823689866	176	190	-	AGCACTCCAAGTTGC
MA0124.2	MA0124.2.Nkx3-1	4.6371975	0.807336205	182	190	-	AGCACTCCA
MA0117.2	MA0117.2.Mafb	7.216008	0.827934274	183	194	+	GGAGTGCTGAAA
MA1615.1	MA1615.1.Plagl1	8.055073	0.815626824	192	204	-	CCCAGGGGCCTTT
MA1604.1	MA1604.1.Ebf2	9.619866	0.845951462	194	206	-	GACCCAGGGGCCT
MA0154.1	MA0154.1.EBF1	8.211776	0.8465123	196	205	-	ACCCAGGGGC
MA0489.2	MA0489.2.Jun	5.932454	0.808179826	198	209	-	AGTGACCCAGGG
MA0494.1	MA0494.1.Nr1h3::Rxra	10.935645	0.855404856	199	217	-	TACCATGGAGTGACCCAGG
MA1988.1	MA1988.1.Atf3	6.375773	0.827451522	199	209	-	AGTGACCCAGG
MA1996.1	MA1996.1.Nr1H2	4.791963	0.808373831	199	209	+	CCTGGGTCACT
MA1110.2	MA1110.2.Nr1H4	5.208099	0.845782725	200	209	+	CTGGGTCACT
MA0099.2	MA0099.2.FOS::JUN	6.8362255	0.860280834	201	207	-	TGACCCA
MA0160.1	MA0160.1.NR4A2	6.5734296	0.822025559	201	208	+	TGGGTCAC
MA0604.1	MA0604.1.Atf1	3.9912841	0.804648407	201	208	-	GTGACCCA
MA1684.1	MA1684.1.Foxn1	0.81124043	0.80424705	202	207	-	TGACCC
MA0067.1	MA0067.1.Pax2	7.3921394	0.934276949	203	210	+	GGTCACTC
MA0027.1	MA0027.1.En1	4.949194	0.822305479	203	213	-	ATGGAGTGACC
MA0519.1	MA0519.1.Stat5a::Stat5b	1.2965746	0.809616397	207	217	+	ACTCCATGGTA
MA0514.2	MA0514.2.Sox3	7.623192	0.861479887	208	218	-	ATACCATGGAG
MA0102.2	MA0102.2.CEBPA	5.898856	0.808245186	215	223	+	GTATGCAAT
MA0650.3	MA0650.3.Hoxa13	6.639357	0.819622438	217	228	+	ATGCAATGAATC
MA1476.2	MA1476.2.Dlx5	6.552939	0.81274401	217	228	+	ATGCAATGAATC
MA0885.2	MA0885.2.Dlx2	6.51233	0.809953652	217	228	+	ATGCAATGAATC
MA0485.1	MA0485.1.Hoxc9	11.4973	0.881848191	218	230	+	TGCAATGAATCAG
MA0909.3	MA0909.3.Hoxd13	5.4041343	0.828220796	218	227	+	TGCAATGAAT
MA0594.1	MA0594.1.Hoxa9	12.581364	0.914415791	219	229	+	GCAATGAATCA
MA0880.1	MA0880.1.Dlx3	6.3074703	0.871372105	219	226	+	GCAATGAA
MA0720.1	MA0720.1.Shox2	6.18787	0.875093031	219	226	+	GCAATGAA
MA0881.1	MA0881.1.Dlx4	6.0494757	0.869907103	219	226	+	GCAATGAA
MA0709.1	MA0709.1.Msx3	5.6772356	0.856650523	219	226	+	GCAATGAA
MA0075.2	MA0075.2.Prrx2	5.65727	0.840278881	219	226	+	GCAATGAA
MA0913.1	MA0913.1.Hoxd9	5.485738	0.812061416	219	228	+	GCAATGAATC
MA0704.1	MA0704.1.Lhx4	4.6083446	0.830344477	219	226	-	TTCATTGC
MA0704.1	MA0704.1.Lhx4	3.7770908	0.811225668	219	226	+	GCAATGAA
MA0705.1	MA0705.1.Lhx8	3.4727485	0.842828341	219	226	-	TTCATTGC
MA0705.1	MA0705.1.Lhx8	2.7634928	0.829431049	219	226	+	GCAATGAA
MA0063.1	MA0063.1.Nkx2-5	5.1885333	0.833776699	220	226	-	TTCATTG

MA0067.1	MA0067.1.Pax2	4.9333587	0.837723697	220	227	-	ATTCATTG
MA0099.2	MA0099.2.FOS::JUN	9.556295	0.959567836	223	229	+	TGAATCA
MA0099.2	MA0099.2.FOS::JUN	7.041783	0.867784013	223	229	-	TGATTCA
MA0705.1	MA0705.1.Lhx8	1.5110977	0.805774271	223	230	-	CTGATTCA
MA0705.1	MA0705.1.Lhx8	1.2680073	0.801182481	223	230	+	TGAATCAG
MA0132.1	MA0132.1.Pdx1	5.029398	0.813150442	225	230	-	CTGATT

E160(1-500)

Matrix ID	Name	Score	Relative score	Start	End	Strand	Predicted sequence
MA0039.2	MA0039.2.Klf4	8.993042	0.889299453	4	13	+	TGGGAGGGTC
MA0493.1	MA0493.1.Klf1	9.320177	0.869171181	4	14	-	GGACCCTCCCA
MA0145.2	MA0145.2.Tfcp2l1	6.6187396	0.827518289	7	20	-	CCAGCTGGACCCTC
MA0499.1	MA0499.1.Myod1	2.8931596	0.827404071	9	21	-	GCCAGCTGGACCC
MA0668.2	MA0668.2.Neurod2	9.898574	0.868626079	9	23	+	GGGTCCAGCTGGCGG
MA0697.2	MA0697.2.Zic3	8.767717	0.822498779	9	21	-	GCCAGCTGGACCC
MA1684.1	MA1684.1.Foxn1	1.8764882	0.826797135	9	14	-	GGACCC
MA0668.2	MA0668.2.Neurod2	7.8468375	0.834110952	10	24	-	CCCGCCAGCTGGACC
MA1618.1	MA1618.1.Ptf1a	6.3444195	0.808239848	10	22	-	CGCCAGCTGGACC
MA1621.1	MA1621.1.Rbpjl	6.2022147	0.802364794	10	23	-	CCGCCAGCTGGACC
MA0500.1	MA0500.1.Myog	9.2007265	0.917291835	11	21	-	GCCAGCTGGAC
MA0521.2	MA0521.2.Tcf12	10.530535	0.926072709	11	22	+	GTCCAGCTGGCG
MA0521.2	MA0521.2.Tcf12	10.528949	0.926039715	11	22	-	CGCCAGCTGGAC
MA0522.1	MA0522.1.Tcf3	12.406403	0.950274422	11	21	-	GCCAGCTGGAC
MA1467.2	MA1467.2.Atoh1	6.277742	0.831908071	11	21	-	GCCAGCTGGAC
MA1472.2	MA1472.2.Bhlha15	9.422424	0.887047405	11	22	-	CGCCAGCTGGAC
MA1472.2	MA1472.2.Bhlha15	9.421379	0.887028632	11	22	+	GTCCAGCTGGCG
MA1618.1	MA1618.1.Ptf1a	6.7740426	0.817134494	11	23	+	GTCCAGCTGGCGG
MA1619.1	MA1619.1.Ptf1A	9.493346	0.889052669	11	22	-	CGCCAGCTGGAC
MA1619.1	MA1619.1.Ptf1A	9.487981	0.888947953	11	22	+	GTCCAGCTGGCG
MA1620.1	MA1620.1.Ptf1A	6.1545687	0.806182679	11	22	-	CGCCAGCTGGAC
MA1628.1	MA1628.1.Zic1::Zic2	4.7625604	0.816249774	11	21	-	GCCAGCTGGAC
MA0461.2	MA0461.2.Atoh1	4.713671	0.810752837	12	21	-	GCCAGCTGGA
MA0499.1	MA0499.1.Myod1	8.476437	0.899179098	12	24	+	TCCAGCTGGCGGG
MA0500.1	MA0500.1.Myog	1.3882854	0.812624727	12	22	+	TCCAGCTGGCG
MA0522.1	MA0522.1.Tcf3	10.6117115	0.926401822	12	22	+	TCCAGCTGGCG
MA0623.1	MA0623.1.Neurog1	6.517405	0.854045346	12	21	+	TCCAGCTGGC
MA0623.1	MA0623.1.Neurog1	5.0959215	0.821038557	12	21	-	GCCAGCTGGA
MA0633.2	MA0633.2.Twist2	12.098128	0.986061784	12	21	-	GCCAGCTGGA
MA0633.2	MA0633.2.Twist2	12.088627	0.985907789	12	21	+	TCCAGCTGGC
MA0697.2	MA0697.2.Zic3	8.12137	0.809471397	12	24	+	TCCAGCTGGCGGG
MA0816.1	MA0816.1.Ascl2	6.417454	0.808780992	12	21	-	GCCAGCTGGA
MA0816.1	MA0816.1.Ascl2	6.1528	0.803524538	12	21	+	TCCAGCTGGC
MA1467.2	MA1467.2.Atoh1	5.918209	0.824793065	12	22	+	TCCAGCTGGCG
MA1628.1	MA1628.1.Zic1::Zic2	4.3786287	0.809507101	12	22	+	TCCAGCTGGCG
MA1993.1	MA1993.1.Neurod2	12.231548	0.981756831	12	21	+	TCCAGCTGGC
MA1993.1	MA1993.1.Neurod2	12.231548	0.981756831	12	21	-	GCCAGCTGGA
MA1997.1	MA1997.1.Olig2	12.022919	0.980907835	12	21	+	TCCAGCTGGC
MA1997.1	MA1997.1.Olig2	12.022919	0.980907835	12	21	-	GCCAGCTGGA
MA0607.1	MA0607.1.Bhlha15	7.7400494	0.890507735	13	20	+	CCAGCTGG
MA0607.1	MA0607.1.Bhlha15	7.7400494	0.890507735	13	20	-	CCAGCTGG
MA0146.2	MA0146.2.Zfx	13.711872	0.931547795	16	29	+	GCTGGCGGGGCCTG
MA0146.2	MA0146.2.Zfx	8.391426	0.838939634	22	35	-	CCGGCTCAGGCCCC
MA2002.1	MA2002.1.Zfp335	8.421604	0.859491168	22	32	-	GCTCAGGCCCC
MA0079.2	MA0079.2.SP1	7.02483	0.808210619	28	37	-	CCCCGGCTCA
MA0472.1	MA0472.1.Egr2	6.959086	0.821728988	29	43	-	GCTCCGCCCCGGCTC
MA0162.1	MA0162.1.Egr1	8.855218	0.82987314	30	40	+	AGCCGGGGCGG
MA0079.2	MA0079.2.SP1	9.500776	0.870565694	33	42	-	CTCCGCCCCG
MA0039.2	MA0039.2.Klf4	10.772671	0.919535015	34	43	+	GGGGCGGAGC
MA0493.1	MA0493.1.Klf1	7.1090775	0.831792823	34	44	-	AGCTCCGCCCC
MA0499.1	MA0499.1.Myod1	3.8101122	0.839191826	35	47	-	GGCAGCTCCGCCC
MA0500.1	MA0500.1.Myog	4.918483	0.859920514	37	47	-	GGCAGCTCCGC
MA0499.1	MA0499.1.Myod1	4.4266005	0.847117003	38	50	+	CGGAGCTGCCTCA
MA0500.1	MA0500.1.Myog	2.2211795	0.823783418	38	48	+	CGGAGCTGCCT
MA0522.1	MA0522.1.Tcf3	3.5336177	0.832250519	38	48	+	CGGAGCTGCCT

MA0633.2	MA0633.2.Twist2	4.339508	0.860313515	38	47	+	CGGAGCTGCC
MA0633.2	MA0633.2.Twist2	4.332823	0.860205164	38	47	-	GGCAGCTCCG
MA0816.1	MA0816.1.Ascl2	6.444969	0.809327493	38	47	+	CGGAGCTGCC
MA1993.1	MA1993.1.Neurod2	3.8275526	0.852334757	38	47	+	CGGAGCTGCC
MA1993.1	MA1993.1.Neurod2	3.8275526	0.852334757	38	47	-	GGCAGCTCCG
MA1997.1	MA1997.1.Olig2	4.2673297	0.85898938	38	47	+	CGGAGCTGCC
MA1997.1	MA1997.1.Olig2	4.2673297	0.85898938	38	47	-	GGCAGCTCCG
MA0100.2	MA0100.2.Myb	3.3776133	0.803412019	39	48	+	GGAGCTGCCT
MA1684.1	MA1684.1.Foxn1	1.5127487	0.819097184	42	47	-	GGCAGC
MA1988.1	MA1988.1.Atf3	5.245525	0.804112449	42	52	+	GCTGCCTCAAC
MA2002.1	MA2002.1.Zfp335	7.599086	0.837245448	42	52	-	GTTGAGGCAGC
MA0145.2	MA0145.2.Tfcp2l1	5.6339917	0.81318679	43	56	-	CAAGGTTGAGGCAG
MA0027.1	MA0027.1.En1	5.0265174	0.825406572	44	54	-	AGGTTGAGGCA
MA0099.2	MA0099.2.FOS::JUN	8.185218	0.909521262	44	50	+	TGCCTCA
MA1684.1	MA1684.1.Foxn1	1.6358447	0.821702986	45	50	-	TGAGGC
MA0643.1	MA0643.1.Esrrg	7.9950194	0.87378865	48	57	-	CCAAGGTTGA
MA0164.1	MA0164.1.Nr2e3	8.265961	0.878576929	49	55	+	CAACCTT
MA0164.1	MA0164.1.Nr2e3	7.3300366	0.845144404	50	56	-	CAAGGTT
MA0079.2	MA0079.2.SP1	8.033814	0.833621229	54	63	-	CCCCTTCCAA
MA0039.2	MA0039.2.Klf4	5.4920053	0.829817491	55	64	+	TGGAAGGGGC
MA0493.1	MA0493.1.Klf1	5.250621	0.800375849	55	65	-	GGCCCCTTCCA
MA1615.1	MA1615.1.Plagl1	12.657733	0.9107533	56	68	+	GGAAGGGGCCACG
MA1684.1	MA1684.1.Foxn1	1.5127487	0.819097184	56	61	+	GGAAGG
MA0067.1	MA0067.1.Pax2	5.2185564	0.848923056	62	69	+	GGCCACGG
MA0869.2	MA0869.2.Sox11	8.756031	0.855708748	71	82	-	ATGAAAAAAGCA
MA1684.1	MA1684.1.Foxn1	0.7151981	0.802213943	72	77	-	AAAAGC
MA0442.1	MA0442.1.SOX10	4.8051834	0.81777575	73	78	+	CTTTTT
MA0606.2	MA0606.2.Nfat5	9.6649685	0.849895639	73	84	-	GGATGAAAAAAG
MA0624.2	MA0624.2.Nfatc1	7.6156282	0.84242938	73	84	-	GGATGAAAAAAG
MA0482.1	MA0482.1.Gata4	2.1613567	0.802864235	74	84	+	TTTTTTCATCC
MA0151.1	MA0151.1.Arid3a	6.027986	0.851116504	77	82	-	ATGAAA
MA0467.2	MA0467.2.Crx	4.5427504	0.811903293	77	86	-	ACGGATGAAA
MA0682.1	MA0682.1.Pitx1	6.51403	0.856888948	78	85	+	TTCATCCG
MA0259.1	MA0259.1.ARNT::HIF1A	4.744789	0.80745403	81	88	+	ATCCGTGA
MA0006.1	MA0006.1.Ahr::Arnt	5.2968884	0.81730469	82	87	+	TCCGTG
MA0154.1	MA0154.1.EBF1	6.858846	0.809552433	82	91	-	ACCTCACGGA
MA0067.1	MA0067.1.Pax2	5.3981495	0.855975452	83	90	-	CCTCACGG
MA0840.1	MA0840.1.Creb5	4.3542843	0.811066096	84	95	-	TCTGACCTCACG
MA0643.1	MA0643.1.Esrrg	2.9909115	0.800174191	85	94	+	GTGAGGTCAG
MA0693.3	MA0693.3.Vdr	8.515099	0.873264114	85	95	+	GTGAGGTCAGA
MA1996.1	MA1996.1.Nr1H2	11.997105	0.969413354	85	95	+	GTGAGGTCAGA
MA0018.2	MA0018.2.CREB1	9.771865	0.934202236	86	93	+	TGAGGTCA
MA0018.2	MA0018.2.CREB1	7.861427	0.864239035	86	93	-	TGACCTCA
MA0676.1	MA0676.1.Nr2e1	5.343548	0.803934509	86	94	+	TGAGGTCAG
MA1110.2	MA1110.2.Nr1H4	11.930009	0.983030203	86	95	+	TGAGGTCAGA
MA0160.1	MA0160.1.NR4A2	8.845187	0.901846936	87	94	+	GAGGTCAG
MA0098.2	MA0098.2.Ets1	6.0458035	0.801147759	92	106	-	ATCCCATCCGCTCTG
MA0668.2	MA0668.2.Neurod2	6.5630627	0.812514779	92	106	+	CAGAGCGGATGGGAT
MA0522.1	MA0522.1.Tcf3	1.9024376	0.810552906	94	104	-	CCCATCCGCTC
MA0079.2	MA0079.2.SP1	6.950546	0.806339824	95	104	-	CCCATCCGCT
MA0623.1	MA0623.1.Neurog1	6.107201	0.844520427	95	104	+	AGCGGATGGG
MA0623.1	MA0623.1.Neurog1	4.8197207	0.814625186	95	104	-	CCCATCCGCT
MA1467.2	MA1467.2.Atoh1	7.290375	0.851947664	95	105	+	AGCGGATGGGA
MA0607.1	MA0607.1.Bhlha15	3.2645593	0.806806409	96	103	-	CCATCCGC
MA0739.1	MA0739.1.Hic1	6.356215	0.846086437	98	106	-	ATCCCATCC
MA0105.1	MA0105.1.NFKB1	9.351667	0.862029582	101	110	+	TGGGATTCCC
MA0105.1	MA0105.1.NFKB1	7.0085974	0.805286863	101	110	-	GGGAATCCCA
MA0467.2	MA0467.2.Crx	4.791085	0.81747723	101	110	+	TGGGATTCCC
MA0105.1	MA0105.1.NFKB1	7.61112	0.819878311	102	111	-	TGGGAATCCC

MA0472.1	MA0472.1.Egr2	5.524899	0.802129444	102	116	+	GGGATTCCCACTCCT
MA1567.2	MA1567.2.Tbx6	7.4062123	0.817509081	104	115	-	GGAGTGGGAATC
MA1684.1	MA1684.1.Foxn1	1.5127487	0.819097184	104	109	-	GGAATC
MA0739.1	MA0739.1.Hic1	5.081851	0.819687452	105	113	+	ATTCCCACT
MA1621.1	MA1621.1.Rbpjl	7.7053475	0.833271152	108	121	+	CCCACTCCTGTTGG
MA0500.1	MA0500.1.Myog	1.6601672	0.816267261	109	119	-	AACAGGAGTGG
MA1620.1	MA1620.1.Ptf1A	8.306673	0.851228949	109	120	+	CCACTCCTGTTG
MA0158.1	MA0158.1.HOXA5	4.2783856	0.80355799	110	117	-	CAGGAGTG
MA0499.1	MA0499.1.Myod1	3.3387585	0.833132404	110	122	+	CACTCCTGTTGGA
MA0522.1	MA0522.1.Tcf3	2.1048229	0.813244992	110	120	+	CACTCCTGTTG
MA1628.1	MA1628.1.Zic1::Zic2	6.30449	0.843329407	112	122	-	TCCAACAGGAG
MA0145.2	MA0145.2.Tfcp2l1	12.188683	0.908580284	114	127	+	CCTGTTGGAGCCTG
MA0145.2	MA0145.2.Tfcp2l1	8.325164	0.852352682	114	127	-	CAGGCTCCAACAGG
MA0079.2	MA0079.2.SP1	7.2639956	0.814233852	120	129	-	CCCAGGCTCC
MA1627.1	MA1627.1.Wt1	8.585675	0.815326011	123	136	-	CTGCTCTCCCAGGC
MA0482.1	MA0482.1.Gata4	2.4483035	0.806989758	127	137	-	TCTGCTCTCCC
MA0498.1	MA0498.1.Meis1	8.769908	0.846068372	128	142	-	GGCTGTCTGCTCTCC
MA0113.2	MA0113.2.NR3C1	8.255775	0.851321272	130	144	+	AGAGCAGACAGCCCC
MA0113.2	MA0113.2.NR3C1	5.2160807	0.813479939	130	144	-	GGGGCTGTCTGCTCT
MA2002.1	MA2002.1.Zfp335	6.3759904	0.804165776	131	141	+	GAGCAGACAGC
MA0105.1	MA0105.1.NFKB1	6.990151	0.804840141	135	144	-	GGGGCTGTCT
MA0442.1	MA0442.1.SOX10	4.823613	0.818593939	135	140	-	CTGTCT
MA0002.2	MA0002.2.Runx1	6.745148	0.829612431	138	148	-	GGCTGGGGCTG
MA0146.2	MA0146.2.Zfx	7.6533427	0.826092489	138	151	-	CTCGGCTGGGGCTG
MA0039.2	MA0039.2.Klf4	4.960873	0.820793654	140	149	-	CGGCTGGGGC
MA0146.2	MA0146.2.Zfx	7.9377666	0.831043195	142	155	+	CCCAGCCGAGCCTG
MA0633.2	MA0633.2.Twist2	4.4641194	0.862333161	142	151	-	CTCGGCTGGG
MA0633.2	MA0633.2.Twist2	4.4640307	0.862331724	142	151	+	CCCAGCCGAG
MA1993.1	MA1993.1.Neurod2	3.471677	0.846854252	142	151	+	CCCAGCCGAG
MA1993.1	MA1993.1.Neurod2	3.471677	0.846854252	142	151	-	CTCGGCTGGG
MA1997.1	MA1997.1.Olig2	4.560688	0.863600996	142	151	+	CCCAGCCGAG
MA1997.1	MA1997.1.Olig2	4.560688	0.863600996	142	151	-	CTCGGCTGGG
MA0145.2	MA0145.2.Tfcp2l1	7.6323056	0.842269191	143	156	+	CCAGCCGAGCCTGG
MA0145.2	MA0145.2.Tfcp2l1	6.775448	0.829798937	143	156	-	CCAGGCTCGGCTGG
MA0499.1	MA0499.1.Myod1	1.6540512	0.811474888	152	164	-	GGCACCCGCCAGG
MA0145.2	MA0145.2.Tfcp2l1	7.5727406	0.841402312	153	166	-	CTGGCACCCGCCAG
MA0145.2	MA0145.2.Tfcp2l1	6.3051734	0.822954812	153	166	+	CTGGCGGGTGCCAG
MA0500.1	MA0500.1.Myog	1.8256353	0.818484118	155	165	+	GGCGGGTGCCA
MA0632.1	MA0632.1.Tcfl5	4.726973	0.834463614	155	164	+	GGCGGGTGCC
MA0632.1	MA0632.1.Tcfl5	4.726973	0.834463614	155	164	-	GGCACCCGCC
MA1099.1	MA1099.1.Hes1	6.981864	0.862141153	155	164	-	GGCACCCGCC
MA1615.1	MA1615.1.Plagl1	7.649301	0.807240449	155	167	+	GGCGGGTGCCAGC
MA0006.1	MA0006.1.Ahr::Arnt	4.890665	0.800069129	157	162	+	CGGGTG
MA0499.1	MA0499.1.Myod1	4.485708	0.847876854	159	171	-	TGCTGCTGGCACC
MA2002.1	MA2002.1.Zfp335	6.718944	0.813441255	159	169	-	CTGCTGGCACC
MA0739.1	MA0739.1.Hic1	10.354962	0.928922165	160	168	+	GTGCCAGCA
MA0522.1	MA0522.1.Tcf3	4.4398074	0.844304462	162	172	+	GCCAGCAGCAA
MA0633.2	MA0633.2.Twist2	2.4362235	0.829465925	162	171	+	GCCAGCAGCA
MA0633.2	MA0633.2.Twist2	2.42681	0.829313356	162	171	-	TGCTGCTGGC
MA0697.2	MA0697.2.Zic3	8.6912	0.820956546	162	174	+	GCCAGCAGCAACT
MA1628.1	MA1628.1.Zic1::Zic2	6.8883886	0.853583932	162	172	+	GCCAGCAGCAA
MA1993.1	MA1993.1.Neurod2	2.2669978	0.828302114	162	171	+	GCCAGCAGCA
MA1993.1	MA1993.1.Neurod2	2.2669978	0.828302114	162	171	-	TGCTGCTGGC
MA1997.1	MA1997.1.Olig2	2.2058506	0.826582771	162	171	+	GCCAGCAGCA
MA1997.1	MA1997.1.Olig2	2.2058506	0.826582771	162	171	-	TGCTGCTGGC
MA0145.2	MA0145.2.Tfcp2l1	10.828096	0.888779035	163	176	+	CCAGCAGCAACTAG
MA0145.2	MA0145.2.Tfcp2l1	7.241642	0.83658368	163	176	-	CTAGTTGCTGCTGG
MA0816.1	MA0816.1.Ascl2	6.2930984	0.806311092	165	174	-	AGTTGCTGCT
MA1684.1	MA1684.1.Foxn1	0.7151981	0.802213943	165	170	+	AGCAGC

MA0885.2	MA0885.2.Dlx2	7.5862455	0.831909951	167	178 +	CAGCAACTAGGC
MA1476.2	MA1476.2.Dlx5	7.6943965	0.835873752	167	178 +	CAGCAACTAGGC
MA0125.1	MA0125.1.Nobox	6.1525025	0.828160686	168	175 -	TAGTTGCT
MA0877.1	MA0877.1.Barhl1	4.1064544	0.81371037	168	177 -	CCTAGTTGCT
MA0705.1	MA0705.1.Lhx8	1.4797329	0.805181814	169	176 -	CTAGTTGC
MA0709.1	MA0709.1.Msx3	3.1593862	0.80035205	169	176 +	GCAACTAG
MA0132.1	MA0132.1.Pdx1	5.029398	0.813150442	171	176 -	CTAGTT
MA1684.1	MA1684.1.Foxn1	1.0789376	0.809913896	175	180 +	AGGCGC
MA1684.1	MA1684.1.Foxn1	1.5127487	0.819097184	179	184 -	GGCAGC
MA1992.1	MA1992.1.Ikzf3	10.363318	0.835356316	179	193 -	AGCCAGGAAGGCAGC
MA2002.1	MA2002.1.Zfp335	6.9159203	0.818768646	179	189 -	AGGAAGGCAGC
MA0062.2	MA0062.2.Gabpa	6.1428537	0.814736638	180	190 -	CAGGAAGGCAG
MA0098.2	MA0098.2.Ets1	8.352369	0.834611875	180	194 +	CTGCCTTCCTGGCTC
MA0474.3	MA0474.3.Erg	10.165129	0.853116402	180	193 -	AGCCAGGAAGGCAG
MA0136.3	MA0136.3.Elf5	9.449978	0.864563295	181	192 -	GCCAGGAAGGCA
MA0518.1	MA0518.1.Stat4	5.7410316	0.826808065	181	194 -	GAGCCAGGAAGGCA
MA1684.1	MA1684.1.Foxn1	1.5127487	0.819097184	183	188 -	GGAAGG
MA0144.1	MA0144.1.Stat3	10.781769	0.886253506	184	193 -	AGCCAGGAAG
MA1615.1	MA1615.1.Plagl1	9.116419	0.837562423	187	199 -	CTGTGGAGCCAGG
MA0014.1	MA0014.1.Pax5	8.830086	0.801586502	189	208 -	AGGCTCCTGCTGTGGAGCCA
MA0499.1	MA0499.1.Myod1	2.8971286	0.827455094	192	204 -	TCCTGCTGTGGAG
MA1629.1	MA1629.1.Zic2	15.5707245	0.939337494	193	206 +	TCCACAGCAGGAGC
MA1472.2	MA1472.2.Bhlha15	6.056386	0.826592741	194	205 +	CCACAGCAGGAG
MA1472.2	MA1472.2.Bhlha15	6.056386	0.826592741	194	205 -	CTCCTGCTGTGG
MA0499.1	MA0499.1.Myod1	4.6015644	0.849366227	195	207 -	GGCTCCTGCTGTG
MA0500.1	MA0500.1.Myog	4.1387324	0.849473816	195	205 +	CACAGCAGGAG
MA0522.1	MA0522.1.Tcf3	5.2314754	0.854835061	195	205 +	CACAGCAGGAG
MA0633.2	MA0633.2.Twist2	2.3603272	0.828235832	195	204 +	CACAGCAGGA
MA0633.2	MA0633.2.Twist2	2.3442285	0.827974911	195	204 -	TCCTGCTGTG
MA0697.2	MA0697.2.Zic3	17.02778	0.98898364	195	207 +	CACAGCAGGAGCC
MA1628.1	MA1628.1.Zic1::Zic2	15.035102	0.996657901	195	205 +	CACAGCAGGAG
MA1993.1	MA1993.1.Neurod2	1.810206	0.821267491	195	204 +	CACAGCAGGA
MA1993.1	MA1993.1.Neurod2	1.810206	0.821267491	195	204 -	TCCTGCTGTG
MA1997.1	MA1997.1.Olig2	1.8521099	0.82102194	195	204 +	CACAGCAGGA
MA1997.1	MA1997.1.Olig2	1.8521099	0.82102194	195	204 -	TCCTGCTGTG
MA0522.1	MA0522.1.Tcf3	2.2673478	0.815406864	197	207 -	GGCTCCTGCTG
MA0500.1	MA0500.1.Myog	1.2480391	0.810745778	198	208 +	AGCAGGAGCCT
MA0632.1	MA0632.1.Tcfl5	3.9553363	0.810024273	198	207 +	AGCAGGAGCC
MA0632.1	MA0632.1.Tcfl5	3.9553363	0.810024273	198	207 -	GGCTCCTGCT
MA0816.1	MA0816.1.Ascl2	6.773673	0.815856088	198	207 -	GGCTCCTGCT
MA1684.1	MA1684.1.Foxn1	0.7151981	0.802213943	201	206 +	AGGAGC
MA0518.1	MA0518.1.Stat4	5.5434914	0.824029069	205	218 -	CTTGGAGGAAAGGC
MA0141.2	MA0141.2.Esrrb	6.53907	0.825033812	211	222 +	CCTCCAAGGAGA
MA0154.1	MA0154.1.EBF1	7.859486	0.836888311	211	220 +	CCTCCAAGGA
MA0519.1	MA0519.1.Stat5a::Stat5b	1.0618684	0.806747996	211	221 +	CCTCCAAGGAG
MA0519.1	MA0519.1.Stat5a::Stat5b	4.1635475	0.844654373	212	222 -	TCTCCTTGGAG
MA0643.1	MA0643.1.Esrrg	3.801728	0.812101955	214	223 +	CCAAGGAGAC
MA0113.2	MA0113.2.NR3C1	9.524972	0.867121571	217	231 -	TGCACAGAGTCTCCT
MA0113.2	MA0113.2.NR3C1	4.7263556	0.807383324	219	233 -	ATTGCACAGAGTCTC
MA1684.1	MA1684.1.Foxn1	1.0789376	0.809913896	220	225 +	AGACTC
MA0442.1	MA0442.1.SOX10	5.845525	0.863961733	223	228 +	CTCTGT
MA0102.2	MA0102.2.CEBPA	6.379334	0.82555303	225	233 +	CTGTGCAAT
MA0518.1	MA0518.1.Stat4	4.307087	0.806635331	229	242 -	CTTCCAAGCATTGC
MA1625.1	MA1625.1.Stat5b	8.349484	0.820019704	230	244 -	CCCTTCCAAGCATTG
MA0519.1	MA0519.1.Stat5a::Stat5b	1.6636782	0.81410286	231	241 +	AATGCTTGGAA
MA1624.1	MA1624.1.Stat5a	9.766613	0.862751437	231	242 -	CTTCCAAGCATT
MA0164.1	MA0164.1.Nr2e3	7.3300366	0.845144404	232	238 -	CAAGCAT
MA0463.1	MA0463.1.Bcl6	8.486702	0.817922281	232	245 +	ATGCTTGGAAGGGA
MA0518.1	MA0518.1.Stat4	8.371533	0.863813965	232	245 +	ATGCTTGGAAGGGA

MA0006.1	MA0006.1.Ahr::Arnt	6.158047	0.853842612	233	238	+	TGCTTG
MA0144.1	MA0144.1.Stat3	10.904741	0.88851143	233	242	+	TGCTTGGAAG
MA0519.1	MA0519.1.Stat5a::Stat5b	7.4460697	0.884770877	233	243	-	CCTTCCAAGCA
MA1684.1	MA1684.1.Foxn1	1.5127487	0.819097184	238	243	+	GGAAGG
MA0136.3	MA0136.3.Elf5	6.922813	0.81619373	239	250	+	GAAGGGAAGCTA
MA1684.1	MA1684.1.Foxn1	9.694712	0.992300042	243	248	+	GGAAGC
MA0164.1	MA0164.1.Nr2e3	7.3300366	0.845144404	245	251	-	CTAGCTT
MA0500.1	MA0500.1.Myog	3.1992257	0.836886782	257	267	-	AGCAGCAGAGC
MA1628.1	MA1628.1.Zic1::Zic2	6.151882	0.84064928	257	267	-	AGCAGCAGAGC
MA0499.1	MA0499.1.Myod1	5.1039505	0.85582458	258	270	+	CTCTGCTGCTGTC
MA0522.1	MA0522.1.Tcf3	3.876056	0.836805559	258	268	+	CTCTGCTGCTG
MA0633.2	MA0633.2.Twist2	1.5513427	0.815124171	258	267	+	CTCTGCTGCT
MA0633.2	MA0633.2.Twist2	1.5445691	0.815014387	258	267	-	AGCAGCAGAG
MA0697.2	MA0697.2.Zic3	10.539677	0.858213324	258	270	-	GACAGCAGCAGAG
MA1993.1	MA1993.1.Neurod2	0.6807067	0.803873128	258	267	+	CTCTGCTGCT
MA1993.1	MA1993.1.Neurod2	0.6807067	0.803873128	258	267	-	AGCAGCAGAG
MA1997.1	MA1997.1.Olig2	1.2993437	0.812332412	258	267	+	CTCTGCTGCT
MA1997.1	MA1997.1.Olig2	1.2993437	0.812332412	258	267	-	AGCAGCAGAG
MA0500.1	MA0500.1.Myog	6.251297	0.87777688	260	270	-	GACAGCAGCAG
MA0521.2	MA0521.2.Tcf12	4.512008	0.800863639	260	271	+	CTGCTGCTGTCT
MA0521.2	MA0521.2.Tcf12	4.5116005	0.800855157	260	271	-	AGACAGCAGCAG
MA0522.1	MA0522.1.Tcf3	6.2524934	0.868416426	260	270	-	GACAGCAGCAG
MA1472.2	MA1472.2.Bhlha15	5.4994617	0.816590278	260	271	+	CTGCTGCTGTCT
MA1472.2	MA1472.2.Bhlha15	5.4989285	0.816580703	260	271	-	AGACAGCAGCAG
MA1628.1	MA1628.1.Zic1::Zic2	7.5961747	0.866014192	260	270	-	GACAGCAGCAG
MA0499.1	MA0499.1.Myod1	7.287935	0.883900481	261	273	+	TGCTGCTGTCTGC
MA0633.2	MA0633.2.Twist2	2.3545773	0.82814264	261	270	-	GACAGCAGCA
MA0633.2	MA0633.2.Twist2	2.3451638	0.82799007	261	270	+	TGCTGCTGTC
MA0816.1	MA0816.1.Ascl2	6.4801226	0.810025696	261	270	+	TGCTGCTGTC
MA0816.1	MA0816.1.Ascl2	6.2298536	0.805054947	261	270	-	GACAGCAGCA
MA1993.1	MA1993.1.Neurod2	2.171253	0.826827637	261	270	+	TGCTGCTGTC
MA1993.1	MA1993.1.Neurod2	2.171253	0.826827637	261	270	-	GACAGCAGCA
MA1997.1	MA1997.1.Olig2	2.0958703	0.824853871	261	270	+	TGCTGCTGTC
MA1997.1	MA1997.1.Olig2	2.0958703	0.824853871	261	270	-	GACAGCAGCA
MA1684.1	MA1684.1.Foxn1	0.7151981	0.802213943	262	267	-	AGCAGC
MA0498.1	MA0498.1.Meis1	9.870868	0.862944069	264	278	+	TGCTGTCTGCCCTGC
MA0442.1	MA0442.1.SOX10	4.823613	0.818593939	266	271	+	CTGTCT
MA0092.1	MA0092.1.Hand1::Tcf3	6.0457377	0.801707716	267	276	+	TGTCTGCCCT
MA1153.1	MA1153.1.Smad4	4.824192	0.817927325	267	274	+	TGTCTGCC
MA1110.2	MA1110.2.Nr1H4	3.6249487	0.813458078	269	278	-	GCAGGGCAGA
MA0697.2	MA0697.2.Zic3	13.065724	0.909126821	270	282	-	CCCAGCAGGGCAG
MA1629.1	MA1629.1.Zic2	12.836786	0.892986497	271	284	-	TCCCCAGCAGGGCA
MA1472.2	MA1472.2.Bhlha15	5.2872405	0.812778748	272	283	-	CCCCAGCAGGGC
MA1472.2	MA1472.2.Bhlha15	5.286724	0.812769473	272	283	+	GCCCTGCTGGGG
MA1628.1	MA1628.1.Zic1::Zic2	14.291339	0.983595808	272	282	-	CCCAGCAGGGC
MA0633.2	MA0633.2.Twist2	2.9411945	0.837650271	273	282	+	CCCTGCTGGG
MA0633.2	MA0633.2.Twist2	2.941106	0.837648838	273	282	-	CCCAGCAGGG
MA1993.1	MA1993.1.Neurod2	2.6071918	0.833541123	273	282	+	CCCTGCTGGG
MA1993.1	MA1993.1.Neurod2	2.6071918	0.833541123	273	282	-	CCCAGCAGGG
MA1997.1	MA1997.1.Olig2	2.4443417	0.830331868	273	282	-	CCCAGCAGGG
MA1997.1	MA1997.1.Olig2	2.4443417	0.830331868	273	282	+	CCCTGCTGGG
MA0518.1	MA0518.1.Stat4	4.2042956	0.805189263	275	288	+	CTGCTGGGGAGCCT
MA0519.1	MA0519.1.Stat5a::Stat5b	4.9723744	0.854539244	276	286	-	GCTCCCCAGCA
MA1684.1	MA1684.1.Foxn1	1.5127487	0.819097184	281	286	+	GGGAGC
MA2002.1	MA2002.1.Zfp335	7.129106	0.82453444	282	292	-	GAGCAGGCTCC
MA1628.1	MA1628.1.Zic1::Zic2	6.80075	0.852044804	284	294	-	GAGAGCAGGCT
MA0482.1	MA0482.1.Gata4	2.1035476	0.802033095	286	296	+	CCTGCTCTCCC
MA1992.1	MA1992.1.Ikzf3	9.418776	0.817143201	292	306	-	GCCAAGGAAGGGGAG
MA0098.2	MA0098.2.Ets1	6.7956204	0.812026252	293	307	+	TCCCCTTCCTTGGCT

MA0474.3	MA0474.3.Erg	8.13028	0.816999587	293	306 -	GCCAAGGAAGGGGA
MA0079.2	MA0079.2.SP1	7.5848827	0.822315181	294	303 +	CCCCTTCCTT
MA0136.3	MA0136.3.Elf5	11.236565	0.898758302	294	305 -	CCAAGGAAGGGG
MA1684.1	MA1684.1.Foxn1	1.5127487	0.819097184	296	301 -	GGAAGG
MA1684.1	MA1684.1.Foxn1	1.5127487	0.819097184	305	310 -	GGCAGC
MA0643.1	MA0643.1.Esrrg	3.0555642	0.801125284	306	315 -	TGTAGGGCAG
MA0493.1	MA0493.1.Klf1	7.123721	0.832040372	309	319 +	CCCTACACCCC
MA0039.2	MA0039.2.Klf4	7.5554543	0.864875105	310	319 -	GGGGTGTAGG
MA0002.2	MA0002.2.Runx1	5.717906	0.809584934	315	325 -	GGGTGTGGGGT
MA0039.2	MA0039.2.Klf4	5.9346504	0.837337949	315	324 -	GGTGTGGGGT
MA0472.1	MA0472.1.Egr2	5.9765077	0.808301111	315	329 +	ACCCCACACCCTGCT
MA1567.2	MA1567.2.Tbx6	7.655079	0.822409928	315	326 -	AGGGTGTGGGGT
MA0079.2	MA0079.2.SP1	8.684709	0.850013568	316	325 +	CCCCACACCC
MA0493.1	MA0493.1.Klf1	12.550466	0.923778799	316	326 +	CCCCACACCCT
MA1992.1	MA1992.1.Ikzf3	9.011523	0.809290373	316	330 -	GAGCAGGGTGTGGGG
MA0039.2	MA0039.2.Klf4	11.875321	0.938268845	317	326 -	AGGGTGTGGG
MA0098.2	MA0098.2.Ets1	7.373314	0.820407544	317	331 +	CCCACACCCTGCTCA
MA1629.1	MA1629.1.Zic2	10.152581	0.847478674	321	334 -	AGCTGAGCAGGGTG
MA1628.1	MA1628.1.Zic1::Zic2	6.957925	0.854805139	322	332 -	CTGAGCAGGGT
MA0633.2	MA0633.2.Twist2	1.2869605	0.810839181	328	337 +	CTCAGCTCAC
MA0633.2	MA0633.2.Twist2	1.2802752	0.810730829	328	337 -	GTGAGCTGAG
MA1997.1	MA1997.1.Olig2	0.90058994	0.806063973	328	337 -	GTGAGCTGAG
MA1997.1	MA1997.1.Olig2	0.90058994	0.806063973	328	337 +	CTCAGCTCAC
MA1110.2	MA1110.2.Nr1H4	3.898056	0.819034363	329	338 +	TCAGCTCACG
MA0162.1	MA0162.1.Egr1	8.642466	0.82451717	330	340 -	TGCGTGAGCTG
MA0632.1	MA0632.1.Tcfl5	4.9303856	0.840906113	331	340 +	AGCTCACGCA
MA0632.1	MA0632.1.Tcfl5	4.9303856	0.840906113	331	340 -	TGCGTGAGCT
MA0067.1	MA0067.1.Pax2	5.005399	0.840552639	332	339 +	GCTCACGC
MA2002.1	MA2002.1.Zfp335	8.590959	0.864071505	332	342 +	GCTCACGCAGC
MA0604.1	MA0604.1.Atf1	4.142156	0.807908418	333	340 +	CTCACGCA
MA0632.1	MA0632.1.Tcfl5	4.3686833	0.823115833	333	342 +	CTCACGCAGC
MA0632.1	MA0632.1.Tcfl5	4.3686833	0.823115833	333	342 -	GCTGCGTGAG
MA0259.1	MA0259.1.ARNT::HIF1A	7.2823167	0.88315468	334	341 -	CTGCGTGA
MA0499.1	MA0499.1.Myod1	10.233001	0.92176036	334	346 -	GGCAGCTGCGTGA
MA1684.1	MA1684.1.Foxn1	0.81124043	0.80424705	334	339 +	TCACGC
MA0006.1	MA0006.1.Ahr::Arnt	9.602817	0.999999989	335	340 -	TGCGTG
MA1618.1	MA1618.1.Ptf1a	6.4984226	0.811428232	335	347 -	TGGCAGCTGCGTG
MA1621.1	MA1621.1.Rbpjl	7.968302	0.838677832	335	348 +	CACGCAGCTGCCAC
MA1621.1	MA1621.1.Rbpjl	6.3059325	0.804497367	335	348 -	GTGGCAGCTGCGTG
MA0500.1	MA0500.1.Myog	13.37749	0.973249986	336	346 -	GGCAGCTGCGT
MA0521.2	MA0521.2.Tcf12	11.545384	0.947185581	336	347 -	TGGCAGCTGCGT
MA0521.2	MA0521.2.Tcf12	11.544841	0.947174272	336	347 +	ACGCAGCTGCCA
MA0522.1	MA0522.1.Tcf3	6.682881	0.874141349	336	346 -	GGCAGCTGCGT
MA1472.2	MA1472.2.Bhlha15	9.80218	0.893867892	336	347 -	TGGCAGCTGCGT
MA1472.2	MA1472.2.Bhlha15	9.8011055	0.893848588	336	347 +	ACGCAGCTGCCA
MA1618.1	MA1618.1.Ptf1a	6.757907	0.816800431	336	348 +	ACGCAGCTGCCAC
MA1619.1	MA1619.1.Ptf1A	10.809121	0.914732674	336	347 -	TGGCAGCTGCGT
MA1619.1	MA1619.1.Ptf1A	10.808135	0.914713428	336	347 +	ACGCAGCTGCCA
MA1620.1	MA1620.1.Ptf1A	7.513071	0.834617854	336	347 +	ACGCAGCTGCCA
MA1620.1	MA1620.1.Ptf1A	7.2882133	0.829911296	336	347 -	TGGCAGCTGCGT
MA0499.1	MA0499.1.Myod1	14.79875	0.980454702	337	349 +	CGCAGCTGCCACC
MA0500.1	MA0500.1.Myog	11.780329	0.951852033	337	347 +	CGCAGCTGCCA
MA0522.1	MA0522.1.Tcf3	11.755898	0.941621554	337	347 +	CGCAGCTGCCA
MA0633.2	MA0633.2.Twist2	12.840679	0.998096719	337	346 +	CGCAGCTGCC
MA0633.2	MA0633.2.Twist2	12.833995	0.997988382	337	346 -	GGCAGCTGCG
MA0816.1	MA0816.1.Ascl2	12.521624	0.930019764	337	346 +	CGCAGCTGCC
MA0816.1	MA0816.1.Ascl2	11.560052	0.910921382	337	346 -	GGCAGCTGCG
MA1993.1	MA1993.1.Neurod2	12.688133	0.988788269	337	346 +	CGCAGCTGCC
MA1993.1	MA1993.1.Neurod2	12.688133	0.988788269	337	346 -	GGCAGCTGCG

MA1997.1	MA1997.1.Olig2	12.706623	0.991655721	337	346	+	CGCAGCTGCC
MA1997.1	MA1997.1.Olig2	12.706623	0.991655721	337	346	-	GGCAGCTGCG
MA0100.2	MA0100.2.Myb	10.784608	0.925423774	338	347	+	GCAGCTGCCA
MA0607.1	MA0607.1.Bhlha15	7.323363	0.882714798	338	345	+	GCAGCTGC
MA0607.1	MA0607.1.Bhlha15	7.323363	0.882714798	338	345	-	GCAGCTGC
MA0498.1	MA0498.1.Meis1	9.867152	0.862887117	340	354	+	AGCTGCCACCCTCAG
MA0739.1	MA0739.1.Hic1	4.8700395	0.815299687	340	348	-	GTGGCAGCT
MA0111.1	MA0111.1.Spz1	10.104356	0.879981497	341	351	-	AGGGTGGCAGC
MA1684.1	MA1684.1.Foxn1	1.5127487	0.819097184	341	346	-	GGCAGC
MA0472.1	MA0472.1.Egr2	6.253155	0.812081766	342	356	+	CTGCCACCCTCAGCC
MA0739.1	MA0739.1.Hic1	9.078984	0.902489744	342	350	+	CTGCCACCC
MA0002.2	MA0002.2.Runx1	6.37024	0.822303086	346	356	-	GGCTGAGGGTG
MA0483.1	MA0483.1.Gfi1B	5.1412354	0.81914402	346	356	+	CACCCTCAGCC
MA0079.2	MA0079.2.SP1	9.739942	0.876588914	350	359	+	CTCAGCCCCC
MA0682.1	MA0682.1.Pitx1	4.694077	0.811528056	350	357	+	CTCAGCCC
MA0124.2	MA0124.2.Nkx3-1	7.9048266	0.869939878	358	366	+	CCCACTTCG
MA0092.1	MA0092.1.Hand1::Tcf3	6.014808	0.800796453	362	371	-	GGTCTCGAAG
MA1995.1	MA1995.1.Npas4	6.316601	0.806903582	362	372	+	CTTCGAGACCG
MA1998.1	MA1998.1.Prdm14	7.703416	0.813407212	362	373	-	CCGGTCTCGAAG
MA1153.1	MA1153.1.Smad4	4.222686	0.804861663	364	371	-	GGTCTCGA
MA0472.1	MA0472.1.Egr2	12.509005	0.897573965	368	382	-	CCCCCACCCCCGGTC
MA1627.1	MA1627.1.Wt1	9.01375	0.822321219	368	381	-	CCCCACCCCCGGTC
MA0162.1	MA0162.1.Egr1	8.712146	0.826271347	369	379	+	ACCGGGGGTGG
MA0079.2	MA0079.2.SP1	8.298484	0.840286753	371	380	-	CCCACCCCCG
MA0079.2	MA0079.2.SP1	12.396375	0.943489426	372	381	-	CCCCACCCCC
MA0039.2	MA0039.2.Klf4	11.45279	0.931090119	373	382	+	GGGGTGGGGG
MA0079.2	MA0079.2.SP1	8.976494	0.857361987	373	382	-	CCCCCACCCC
MA0493.1	MA0493.1.Klf1	8.411387	0.853808208	373	383	-	CCCCCCACCCC
MA0079.2	MA0079.2.SP1	9.581803	0.872606306	374	383	-	CCCCCCACCC
MA1627.1	MA1627.1.Wt1	9.887895	0.836605693	374	387	-	CAGCCCCCCCACCC
MA0079.2	MA0079.2.SP1	9.186873	0.862660258	375	384	-	CCCCCCCACC
MA0734.3	MA0734.3.Gli2	9.268459	0.843636174	375	387	-	CAGCCCCCCCACC
MA1630.1	MA1630.1.Znf281	7.3238654	0.816613747	375	385	+	GGTGGGGGGGC
MA1990.1	MA1990.1.Gli1	9.437069	0.827598042	375	388	-	CCAGCCCCCCCACC
MA0079.2	MA0079.2.SP1	7.714125	0.825570066	376	385	-	GCCCCCCCAC
MA0079.2	MA0079.2.SP1	12.262342	0.940113912	380	389	-	CCCAGCCCCC
MA0039.2	MA0039.2.Klf4	4.758022	0.817347248	381	390	+	GGGGCTGGGA
MA1567.2	MA1567.2.Tbx6	7.300827	0.815433764	382	393	+	GGGCTGGGAAGG
MA0144.1	MA0144.1.Stat3	7.006118	0.816927962	383	392	+	GGCTGGGAAG
MA0739.1	MA0739.1.Hic1	4.9967604	0.817924764	383	391	-	TTCCCAGCC
MA0079.2	MA0079.2.SP1	7.634672	0.823569095	385	394	-	CCCTTCCCAG
MA0145.2	MA0145.2.Tfcp2l1	6.872129	0.831205983	385	398	-	GCAGCCCTTCCCAG
MA0039.2	MA0039.2.Klf4	5.8310504	0.835577804	386	395	+	TGGGAAGGGC
MA1684.1	MA1684.1.Foxn1	1.5127487	0.819097184	388	393	+	GGAAGG
MA0515.1	MA0515.1.Sox6	5.4928484	0.819211902	397	406	+	GCAGTGTCTG
MA0067.1	MA0067.1.Pax2	5.585006	0.863313083	398	405	-	AGACACTG
MA0442.1	MA0442.1.SOX10	6.8124285	0.906887448	398	403	+	CAGTGT
MA2002.1	MA2002.1.Zfp335	8.050335	0.849449871	399	409	-	AGTCAGACACT
MA1684.1	MA1684.1.Foxn1	1.0789376	0.809913896	400	405	-	AGACAC
MA1153.1	MA1153.1.Smad4	4.824192	0.817927325	401	408	+	TGTCTGAC
MA0489.2	MA0489.2.Jun	10.507993	0.902079786	402	413	-	TGTGAGTCAGAC
MA0498.1	MA0498.1.Meis1	7.2907987	0.823396337	402	416	-	GCCTGTGAGTCAGAC
MA0489.2	MA0489.2.Jun	12.44356	0.94180181	403	414	+	TCTGACTCACAG
MA1988.1	MA1988.1.Atf3	13.037022	0.965003066	403	413	+	TCTGACTCACA
MA1988.1	MA1988.1.Atf3	10.28149	0.908102685	403	413	-	TGTGAGTCAGA
MA0099.2	MA0099.2.FOS::JUN	10.663977	1.000000015	405	411	+	TGACTCA
MA0099.2	MA0099.2.FOS::JUN	7.999927	0.902757844	405	411	-	TGAGTCA
MA1684.1	MA1684.1.Foxn1	0.81124043	0.80424705	405	410	+	TGACTC
MA0464.1	MA0464.1.Bhlhe40	4.2936287	0.841140237	408	418	+	CTCACAGGCAG

MA2002.1	MA2002.1.Zfp335	9.1853485	0.880147294	409	419	+	TCACAGGCAGA
MA0006.1	MA0006.1.Ahr::Arnt	5.2968884	0.81730469	412	417	-	TGCCTG
MA0092.1	MA0092.1.Hand1::Tcf3	7.422879	0.842281785	412	421	-	TGTCTGCCTG
MA1153.1	MA1153.1.Smad4	4.824192	0.817927325	414	421	-	TGTCTGCC
MA1621.1	MA1621.1.Rbpjl	6.658376	0.811744065	416	429	+	CAGACACCTCCCTT
MA1620.1	MA1620.1.Ptf1A	6.0497026	0.8039877	417	428	+	AGACACCTCCCT
MA1684.1	MA1684.1.Foxn1	1.0789376	0.809913896	417	422	+	AGACAC
MA0464.1	MA0464.1.Bhlhe40	1.656134	0.805552308	418	428	+	GACACCTCCCT
MA0493.1	MA0493.1.Klf1	5.9009504	0.811369588	418	428	+	GACACCTCCCT
MA0499.1	MA0499.1.Myod1	1.4655964	0.809052235	418	430	+	GACACCTCCCTTC
MA0522.1	MA0522.1.Tcf3	3.277586	0.828844838	418	428	+	GACACCTCCCT
MA0039.2	MA0039.2.Klf4	5.977541	0.838066651	419	428	-	AGGGAGGTGT
MA0079.2	MA0079.2.SP1	9.208314	0.863200223	424	433	+	TCCCTTCCCC
MA0136.3	MA0136.3.Elf5	7.3079085	0.823564403	424	435	-	AGGGGGAAGGGA
MA0079.2	MA0079.2.SP1	12.075676	0.93541284	425	434	+	CCCTTCCCCC
MA1684.1	MA1684.1.Foxn1	1.5127487	0.819097184	426	431	-	GGAAGG
MA1684.1	MA1684.1.Foxn1	0.7151981	0.802213943	443	448	+	AGAATC
MA0067.1	MA0067.1.Pax2	4.169103	0.807712336	445	452	+	AATCGCTC
MA0519.1	MA0519.1.Stat5a::Stat5b	2.861979	0.828747586	449	459	+	GCTCCCCAAAA
MA1684.1	MA1684.1.Foxn1	1.5127487	0.819097184	449	454	-	GGGAGC
MA0442.1	MA0442.1.SOX10	4.5206213	0.805142608	456	461	-	CATTTT
